# Ferroptosis in health and disease

**DOI:** 10.1016/j.redox.2024.103211

**Published:** 2024-05-30

**Authors:** Carsten Berndt, Hamed Alborzinia, Vera Skafar Amen, Scott Ayton, Uladzimir Barayeu, Alexander Bartelt, Hülya Bayir, Christina M. Bebber, Kivanc Birsoy, Jan P. Böttcher, Simone Brabletz, Thomas Brabletz, Ashley R. Brown, Bernhard Brüne, Giorgia Bulli, Alix Bruneau, Quan Chen, Gina M. DeNicola, Tobias P. Dick, Ayelén Distéfano, Scott J. Dixon, Jan B. Engler, Julia Esser-von Bieren, Maria Fedorova, José Pedro Friedmann Angeli, Manuel A. Friese, Dominic C. Fuhrmann, Ana J. García-Sáez, Karolina Garbowicz, Magdalena Götz, Wei Gu, Linda Hammerich, Behrouz Hassannia, Xuejun Jiang, Aicha Jeridi, Yun Pyo Kang, Valerian E. Kagan, David B. Konrad, Stefan Kotschi, Peng Lei, Marlène Le Tertre, Sima Lev, Deguang Liang, Andreas Linkermann, Carolin Lohr, Svenja Lorenz, Tom Luedde, Axel Methner, Bernhard Michalke, Anna V. Milton, Junxia Min, Eikan Mishima, Sebastian Müller, Hozumi Motohashi, Martina U. Muckenthaler, Shohei Murakami, James A. Olzmann, Gabriela Pagnussat, Zijan Pan, Thales Papagiannakopoulos, Lohans Pedrera Puentes, Derek A. Pratt, Bettina Proneth, Lukas Ramsauer, Raphael Rodriguez, Yoshiro Saito, Felix Schmidt, Carina Schmitt, Almut Schulze, Annemarie Schwab, Anna Schwantes, Mariluz Soula, Benedikt Spitzlberger, Brent R. Stockwell, Leonie Thewes, Oliver Thorn-Seshold, Shinya Toyokuni, Wulf Tonnus, Andreas Trumpp, Peter Vandenabeele, Tom Vanden Berghe, Vivek Venkataramani, Felix C.E. Vogel, Silvia von Karstedt, Fudi Wang, Frank Westermann, Chantal Wientjens, Christoph Wilhelm, Michele Wölk, Katherine Wu, Xin Yang, Fan Yu, Yilong Zou, Marcus Conrad

**Affiliations:** a Department of Neurology, Medical Faculty, Heinrich-Heine University, Düsseldorf, Germany; b Heidelberg Institute for Stem Cell Technology and Experimental Medicine (HI-STEM GGmbH), Heidelberg, Germany; c Division of Stem Cells and Cancer, German Cancer Research Center (DKFZ) and DKFZ-ZMBH Alliance, Heidelberg, Germany; d Rudolf Virchow Zentrum, Center for Integrative and Translational Bioimaging - University of Würzburg, Germany; e Florey Institute of Neuroscience and Mental Health, University of Melbourne, Australia; f Division of Redox Regulation, DKFZ-ZMBH Alliance, German Cancer Research Center (DKFZ) Heidelberg, Germany; g Faculty of Biosciences, Heidelberg University, 69120, Heidelberg, Germany; h Department of Environmental Medicine and Molecular Toxicology, Tohoku University Graduate School of Medicine, Sendai, Japan; i Institute for Cardiovascular Prevention (IPEK), Faculty of Medicine, Ludwig-Maximilians-Universität München, Munich, Germany; j Institute for Diabetes and Cancer (IDC), Helmholtz Center Munich, Neuherberg, Germany; k German Center for Cardiovascular Research, Partner Site Munich Heart Alliance, Munich, Germany; l Department of Pediatrics, Columbia University, New York City, NY, USA; m University of Cologne, Faculty of Medicine and University Hospital Cologne, Department of Translational Genomics, Cologne, Germany; n CECAD Cluster of Excellence, University of Cologne, Cologne, Germany; o Laboratory of Metabolic Regulation and Genetics, Rockefeller University, New York City, NY, USA; p Institute of Molecular Immunology, School of Medicine, Technical University of Munich (TUM), Germany; q Department of Experimental Medicine 1, Nikolaus-Fiebiger Center for Molecular Medicine, Friedrich-Alexander University of Erlangen-Nürnberg, Germany; r Department of Biological Sciences, Columbia University, New York City, NY, USA; s Institute of Biochemistry1-Pathobiochemistry, Goethe-Universität, Frankfurt Am Main, Germany; t Department of Physiological Genomics, Ludwig-Maximilians-University, Munich, Germany; u Department of Hepatology and Gastroenterology, Charité - Universitätsmedizin Berlin, Campus Virchow-Klinikum (CVK) and Campus Charité Mitte (CCM), Berlin, Germany; v College of Life Sciences, Nankai University, Tianjin, China; w Department of Metabolism and Physiology, Moffitt Cancer Center, Tampa, FL, USA; x Instituto de Investigaciones Biológicas, CONICET, National University of Mar Del Plata, Argentina; y Department of Biology, Stanford University, Stanford, CA, USA; z Institute of Neuroimmunology and Multiple Sclerosis, University Medical Center Hamburg-Eppendorf, Germany; aa Department of Immunobiology, Université de Lausanne, Switzerland; ab Center of Membrane Biochemistry and Lipid Research, University Hospital Carl Gustav Carus and Faculty of Medicine of TU Dresden, Germany; ac Institute for Genetics, CECAD, University of Cologne, Germany; ad Max Planck Institute of Biophysics, Frankfurt/Main, Germany; ae Division of Neuroblastoma Genomics, DKFZ, Heidelberg, Germany; af Institute of Stem Cell Research, Helmholtz Center Munich, Germany; ag Institute for Cancer Genetics, And Herbert Irving Comprehensive Cancer Center, Vagelos College of Physicians & Surgeons, Columbia University, New York, NY, USA; ah Department of Pathology and Cell Biology, Vagelos College of Physicians & Surgeons, Columbia University, New York, NY, USA; ai Department of Biomedical Sciences, University of Antwerp, Belgium; aj Sloan Kettering Institute, Memorial Sloan Kettering Cancer Center, New York City, NY, USA; ak Institute of Lung Health and Immunity (LHI), Helmholtz Munich, Comprehensive Pneumology Center (CPC-M), Germany, Member of the German Center for Lung Research (DZL); al College of Pharmacy and Research Institute of Pharmaceutical Science, Seoul National University, Republic of Korea; am School of Public Health, University of Pittsburgh, PA, USA; an Department of Pharmacy, Ludwig-Maximilians-University, Munich, Germany; ao State Key Laboratory of Biotherapy, West China Hospital, Sichuan University, Chengdu, China; ap Center for Translational Biomedical Iron Research, Heidelberg University, Germany; aq Department of Molecular Cell Biology, Weizmann Institute of Science, Rehovot, Israel; ar Division of Nephrology, Department of Internal Medicine III, University Hospital Carl Gustav Carus at the Technische Universität Dresden, Germany; as Division of Nephrology, Department of Medicine, Albert Einstein College of Medicine, New York, NY, USA; at Department of Gastroenterology, Hepatology and Infectious Diseases, Medical Faculty, Heinrich-Heine University, Düsseldorf, Germany; au Institute of Metabolism and Cell Death, Helmholtz Center Munich, Germany; av Institute of Molecular Medicine, Johannes Gutenberg-Universität Mainz, Germany; aw Research Unit Analytical Biogeochemistry, Helmholtz Center Munich, Germany; ax School of Medicine, Zhejiang University, Hangzhou, China; ay Cellular and Chemical Biology, Institut Curie, Paris, France; az Department of Gene Expression Regulation, Tohoku University, Sendai, Japan; ba Department of Molecular and Cell Biology, University of California, Berkeley, CA, USA; bb Department of Nutritional Sciences and Toxicology, University of California, Berkeley, CA, USA; bc Chan Zuckerberg Biohub, San Francisco, CA, USA; bd School of Life Sciences, Westlake University, Hangzhou, China; be Department of Pathology, Grossman School of Medicine, New York University, NY, USA; bf Department of Chemistry and Biomolecular Sciences, University of Ottawa, Canada; bg Graduate School of Pharmaceutical Sciences, Tohoku University, Sendai, Japan; bh Division of Tumour Metabolism and Microenvironment, DKFZ Heidelberg and DKFZ-ZMBH Alliance, Heidelberg, Germany; bi Center of Allergy and Environment (ZAUM), Technical University of Munich and Helmholtz Center Munich, Munich, Germany; bj Department of Chemistry, Columbia University, New York, NY, USA; bk Department of Pathology and Biological Responses, Nagoya University Graduate School of Medicine, Nagoya, Japan; bl Center for Low-temperature Plasma Sciences, Nagoya University, Nagoya, Japan; bm Center for Integrated Sciences of Low-temperature Plasma Core Research (iPlasma Core), Tokai National Higher Education and Research System, Nagoya, Japan; bn German Cancer Consortium (DKTK), Heidelberg, Germany; bo VIB-UGent Center for Inflammation Research, Ghent, Belgium; bp Department of Biomedical Molecular Biology, Ghent University, Ghent, Belgium; bq Comprehensive Cancer Center Mainfranken, University Hospital Würzburg, Germany; br University of Cologne, Faculty of Medicine and University Hospital Cologne, Center for Molecular Medicine Cologne, Germany; bs Immunopathology Unit, Institute of Clinical Chemistry and Clinical Pharmacology, Medical Faculty, University Hospital Bonn, University of Bonn, Germany; bt Westlake Four-Dimensional Dynamic Metabolomics (Meta4D) Laboratory, Westlake Laboratory of Life Sciences and Biomedicine, Hangzhou, Zhejiang, China

**Keywords:** Cell death, Lipid peroxidation, Iron, Ischemia/reperfusion, Cancer, Neurodegeneration

## Abstract

Ferroptosis is a pervasive non-apoptotic form of cell death highly relevant in various degenerative diseases and malignancies. The hallmark of ferroptosis is uncontrolled and overwhelming peroxidation of polyunsaturated fatty acids contained in membrane phospholipids, which eventually leads to rupture of the plasma membrane. Ferroptosis is unique in that it is essentially a spontaneous, uncatalyzed chemical process based on perturbed iron and redox homeostasis contributing to the cell death process, but that it is nonetheless modulated by many metabolic nodes that impinge on the cells’ susceptibility to ferroptosis. Among the various nodes affecting ferroptosis sensitivity, several have emerged as promising candidates for pharmacological intervention, rendering ferroptosis-related proteins attractive targets for the treatment of numerous currently incurable diseases. Herein, the current members of a Germany-wide research consortium focusing on ferroptosis research, as well as key external experts in ferroptosis who have made seminal contributions to this rapidly growing and exciting field of research, have gathered to provide a comprehensive, state-of-the-art review on ferroptosis. Specific topics include: basic mechanisms, *in vivo* relevance, specialized methodologies, chemical and pharmacological tools, and the potential contribution of ferroptosis to disease etiopathology and progression. We hope that this article will not only provide established scientists and newcomers to the field with an overview of the multiple facets of ferroptosis, but also encourage additional efforts to characterize further molecular pathways modulating ferroptosis, with the ultimate goal to develop novel pharmacotherapies to tackle the various diseases associated with – or caused by – ferroptosis.

## Highlights

• Various disparate metabolic pathways contribute to ferroptosis sensitivity.
• Ferroptosis is regulated at the transcriptional and (post)translational levels.
• Small molecules readily available to modulate ferroptosis *in vitro* and in vivo
• Ferroptotic cell death plays a major pathological role in multiple diseases.
• Ferroptosis modulation is a promising therapeutic strategy for clinical applications.

## Graphical abstract

**Figure:**
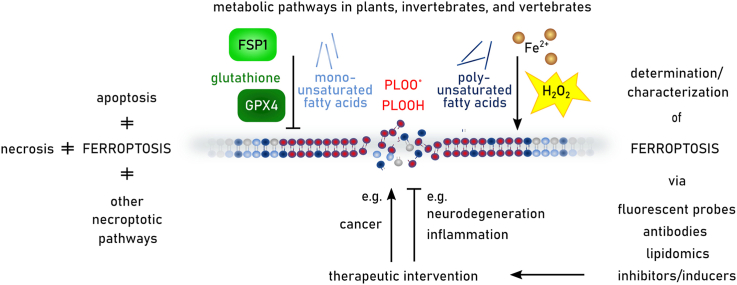

## List of abbreviations

ACSL: acyl-CoA synthetase long chain family member
AIFM: apoptosis inducing factor mitochondria associated
AKT: serine/threonine kinase
ALP: autophagy-lysosomal pathway
AML: acute myeloid leukemia
AMPK: AMP-activated protein kinase
ATF4: activating transcription factor 4
APOE: apolipoprotein
APOER2: apolipoprotein E receptor 2
Aβ: β-amyloid
BAP1: BRCA1-associated protein 1
BH2: oxidized BH4 (dihydrobiopterin)
BH4: tetrahydrobiopterin
CBS: cystathionine β-synthase
CD: cluster of differentiation
CDI: cysteine-deprivation-induced
CDKN2A: cyclin dependent kinase inhibitor 2A
CE-ICP-MS: capillary electrophoresis-inductively coupled plasma mass spectrometry
CHAC1: ChaC glutathione specific gamma-glutamylcyclotransferase 1
CHMP: charged multivesicular body protein
CISD: CDGSH iron sulfur domain
CNS: central nervous system
CoA: co-enzyme A
CoQ_10_: co-enzyme Q_10_
CSE: cystathionine γ-lyase
DAMP: damage-associated molecular pattern
DC: dendritic cell
DDI2: DNA damage inducible 1 homolog 2
DECR1: 2,4-Dienoyl-CoA reductase 1
DHFR: dihydrofolate reductase
DHODH: dihydroorotate dehydrogenase
DMT1: divalent metal transporter 1
EAE: experimental autoimmune encephalomyelitis
ELOVL: elongation of very long chain fatty acids protein
ER: endoplasmic reticulum
ESCRT-III: endosomal sorting complex required for transport III
ETC: electron transport chain
FADS: fatty acid desaturase
FIN: ferroptosis inducer
FPN1: ferroportin 1
FSP1: ferroptosis suppressor protein 1
FTH: ferritin heavy chain
FTL: ferritin light chain
FUNDC2: FUN14 domain containing 2
GCH1: GTP cyclohydrolase 1
GCIB: gas cluster ion beams
GFRP: GCH1 feedback regulator
GPX: glutathione peroxidase
GRFS1: guanine-rich sequence-binding factor 1
GSH: glutathione
GSS: glutathione synthase
GSSG: oxidized glutathione
HAT: H-atom transfer
HCC: hepatocellular carcinoma
HMGB1: high mobility group box 1
HNSCC: head and neck squamous cell carcinoma
HO-1: heme oxygenase 1
HOIP: HOIL-interacting protein
HPLC: high-performance liquid chromatography
HS: heat shock
ICP-MS: coupled plasma mass spectrometry
IDH: isocitrate dehydrogenase
IEC: intestinal epithelial cell
IFN: interferon
IKE: imidazole ketone erastin
IL: interleukin
ILC2: group 2 innate lymphoid cells
IMS: ion mass spectrometry
I/R: ischemia/reperfusion
IRI: ischemia/reperfusion injury
IRP: iron regulatory protein
KEAP1: Kelch-like ECH-associated protein 1
KRAS: KRAS proto-oncogene, GTPase
LDH: lactate dehydrogenase
LRP: LDL receptor related protein
LIP: labile iron pool
LPLAT: lysophospholipid acyltransferases
LPCAT: lysophosphatidylcholine acyltransferase
LTP: low-temperature plasma
MALDI: matrix-assisted laser desorption/ionization
MBOAT: membrane bound O-acyl transferase
MCAO: middle cerebral artery occlusion
MS: mass specrometry
mTORC1: rapamycin kinase complex 1
MUFA: monounsaturated fatty acid
NCOA4: nuclear receptor coactivator 4
NET: neutrophil extracellular trap
NFE2L1: NFE2 like BZIP transcription factor 1
NK: natural killer
NOS: nitric oxide synthase
NOX: NADPH oxidase
NP-LC: normal-phase liquid chromatography
NRF2: Keap1/nuclear factor-erythroid 2-related factor 2
NSCLC: non-small-cell lung cancer
NTBI: non-transferrin-bound iron
Orai: calcium release-activated calcium channel protein
OsFER2: rice ferritin 2
OTUB1: OTU deubiquitinase ubiquitin aldehyde binding 1
OXPHOS: oxidative phosphorylation
PAL: plasma-activated lactate
PC: phosphatidylcholines
PCBP: poly rC binding protein
PE: phosphatidylethanolamine
PEG: polyethyleneglycol
PGE_2_: prostaglandin E2
PKCβII: protein kinase CβII
PL: phospholipid
PUFA: polyunsaturated fatty acid
PUFA-PL: polyunsaturated glycerophospholipid
PPAR: peroxisome proliferator-activated receptor
PPP: pentose phosphate pathway
PTGS2: prostaglandin-endoperoxide synthase 2
RA: rheumatoid arthritis
RAS: rat sarcome
RCS: reactive carbonyl species
RIPK: receptor-interacting protein kinase
ROS: reactive oxygen species
RP-LC: reverse-phase liquid chromatography
RSL3: RAS selective lethal 3
RTA: radical trapping antioxidant
SBP2: SECIS-binding protein 2
SCD: stearoyl-CoA desaturase
SCLC: small cell lung cancer
SECIS: selenocysteine-insertion sequence
SEC24B: SEC24 homolog B coat protein complex II component
SELENOP: selenoprotein P
SEPHS2: selenophosphate synthetase 2
SIMS: secondary ion mass spectrometry
SLC: solute carrier family
SLE: systemic lupus erythematosus
SOD: superoxide dismutase
STARD7: StAR related lipid transfer domain containing 7
SREBP1: sterol regulatory element binding protein 1
SUMO: small ubiquitin-like modifier
TBI: traumatic brain injury
TCA: tricarboxylic acid
TfR1: transferrin receptor 1
TMEM: transmembrane protein
TNF: tumor necrosis factor
TRSP: selenocysteine tRNA^[Ser]Sec^
Trx: thioredoxin
TrxR: thioredoxin reductase
TUNEL: terminal deoxynucleotidyl transferase-mediated dUTP nick-end labeling
UBIAD1: UbiA prenyltransferase domain containing 1
UC: ulcerative colitis
UPS: ubiquitin-proteasome system
VDAC: voltage-dependent anion-selective channel protein
YAP: yes-associated transcriptional regulator
ZEB1: zinc finger E-box binding homeobox
αESA: α-eleostearic acid
γ-GCL: γ-glutamyl cysteine ligase
4-HNE: 4-hydroxy-2-nonenal

## 1 Introduction

### 1.1 Historic overview

**Fig. 1:**
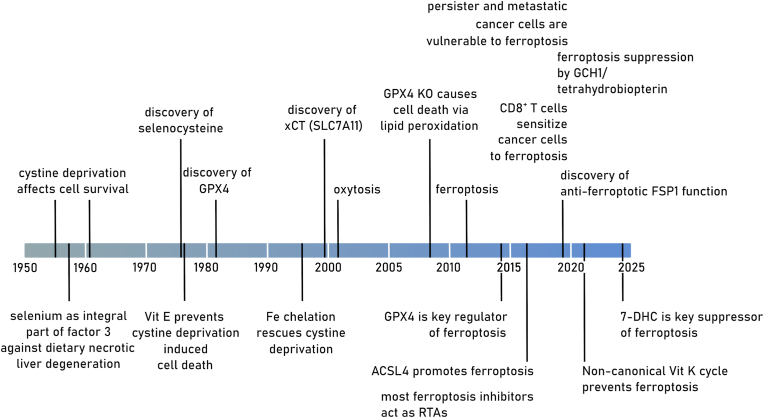
Timeline with milestones in ferroptosis research. The presented timeline summarizes milestones in selenium and (seleno-)cyteine research as prerequisites for the understanding of ferroptosis and more recent research directly connected to ferroptosis. ACSL4: acyl-CoA synthetase long chain family member 4, FSP1: ferroptosis suppressor protein 1, GCH1: GTP cyclohydrolase 1, GPX4: glutathione peroxidase 4, RTA: radical trapping antioxidant, SLC7A11: solute carrier family 7 member 11, 7-DHC: 7-dehydrocholesterin.

Ferroptosis is a non-apoptotic mechanism of cell death that emerges from the interaction between iron, oxygen, and oxidizable phospholipids (PLs). Compared to other forms of cell death, ferroptosis is uniquely distinguished by the accumulation of (phospho)lipid hydroperoxides [1]. Lipid peroxidation is a chemical chain reaction that involves the formation of carbon-centered radicals on polyunsaturated lipid molecules, their combination with molecular oxygen and the reaction of the resultant peroxyl radical with adjacent lipids to propagate the process [2]. In the cellular context, lipid peroxidation eventually leads to plasma membrane rupture, the hallmark of all forms of cell death [3]. For these reasons, ferroptosis can be usefully (operationally) defined as cell death that is best prevented by small molecule inhibitors of lipid peroxidation. For example, among the most potent and specific inhibitors of lipid peroxidation and ferroptosis are the synthetic lipophilic radical trapping antioxidants (RTAs) ferrostatin-1 and liproxstatin-1 [[4], [5], [6]]. How lipid peroxidation is initiated and inhibited, how lipid peroxidation causes ferroptosis, and how sensitivity to this form of cell death is governed by the interaction of different signaling and metabolic pathways is still a matter of intensive investigation. Specific examples will be presented throughout this comprehensive review. Below, we briefly touch on the diverse strands of biochemical, genetic, and chemical research, some initiated decades ago, that ultimately gave rise to our current understanding of ferroptosis (Fig. 1).

Chemical reactions between iron, oxygen, and polyunsaturated lipids are potentially deadly for any cell. Even simple organisms appear to be susceptible to ferroptosis and employ a variety of strategies to defend against lethal amounts of (phospho)lipid hydroperoxides [[7], [8], [9], [10]]. Thus, defense mechanisms to minimize lipid peroxidation are needed by any cell containing oxidizable lipids. These mechanisms generally rely on redox active molecules that can donate electrons and, directly or indirectly, prevent the initiation or mitigate the spread of lipid peroxidation. Thiol (-SH)-containing metabolites, most importantly the amino acid l-cysteine and the cysteine-containing tripeptide glutathione (γ-L-glutamyl-l-cysteinylglycine, GSH), are important in this connection. Landmark studies conducted in the 1950's and the 1960's established that cystine (the disulfide of cysteine) was required for the survival and proliferation of many mammalian cells grown in culture [11,12]. Studies later in the 20th century also showed that the death of mammalian cells deprived of extracellular cystine coincided with the loss of intracellular GSH and unrestrained lipid peroxidation that could be prevented by iron chelators or the addition of the natural RTA α-tocopherol (vitamin E) [[13], [14], [15], [16]]. In retrospect, it seems clear that these early studies were investigating ferroptosis. Indeed, at different times, cystine-deprivation-induced cell death was described by specific names, such as oxidative glutamate toxicity or oxytosis [[17], [18], [19], [20]]. However, during this period it was unclear whether this cystine deprivation phenotype was relevant beyond that cellular condition, exactly how cells were dying, and whether this mechanism was truly distinguishable from apoptosis or not.

While the above-mentioned studies showed a key role for cystine and GSH in preventing cell death, they did not directly establish how these metabolites were required to prevent lipid peroxidation and cell death. This understanding evolved from parallel lines of investigation focused on the biochemical purification and genetic dissection of enzymes that can directly detoxify phospholipid hydroperoxides. Biochemical purification in the 1980's resulted in the isolation of a selenoenzyme that could efficiently reduce lipid hydroperoxides to lipid alcohols in lipid bilayers [21,22]. This enzyme was originally named phospholipid hydroperoxide GSH peroxidase and, eventually, GSH peroxidase 4 (GPX4). Genetic studies undertaken in mice in the early 2000's found that *Gpx4* deletion was to be embryonic lethal, consistent with an important function for this enzyme *in vivo* [23]. In 2008, inducible disruption of *Gpx4* in cells isolated from mutant mice was found to cause a novel form of oxidative cell death, distinct from apoptosis, and preventable by vitamin E [24]. This form of cell death was also evident in neurons lacking *Gpx4* demonstrating that GPX4-dependent cell death is operating *in vivo*. Later on, it was clearly demonstrated that *Gpx4* disruption in adult mice caused a fatal destruction of kidney tissues that could be prevented by ferroptosis-specific inhibitors, firmly establishing the important role of GPX4 in preventing ferroptosis [6]. Studies of GPX4 (and subsequently other enzymes, see below) unified and extended the mechanism of ferroptosis beyond the cystine deprivation paradigm that drove earlier work in this area.

Ultimately, the recognition (and naming) of ferroptosis as a unique mechanism of cell death emerged from chemical-biology-driven studies undertaken in the 2000's and 2010's. The availability of chemical libraries and high-throughput phenotypic screening platforms enabled the discovery of a series of small molecules that triggered an iron-dependent, oxidative, non-apoptotic form of cell death by preventing the uptake of cystine by the system xCT cystine/glutamate antiporter (i.e., erastin) or by covalently inhibiting GPX4 (i.e., rat sarcome (RAS) selective lethal 3 (RSL3)) [[25], [26], [27], [28], [29]]. Related screens also identified the aforementioned synthetic RTAs, ferrostatin-1 and liproxstatin-1, which specifically prevent ferroptosis. The inherent portability of these tools enabled ferroptosis to be interrogated in an ever-growing number of cells and organisms. These small molecules also served as prototypes for the development of newer agents that may be useful clinically and provide the ultimate validation of the importance of the ferroptosis mechanism in humans [[30], [31], [32], [33], [34], [35]].

It now seems clear that ferroptosis is a mechanism that was being studied under different guises for decades. One might ask: why did the concept of ferroptosis only take firm root after 2012? One can only speculate. But a confluence of events seems to have contributed to the rapid acceptance and expansion of this concept in recent times [36]. First, the discovery that non-apoptotic cell death could be a regulated process in the early 2000's stimulated great interest in discovering and clearly demarcating different forms of non-apoptotic cell death from one another [37,38]. Second, the recognition that ferroptosis is not ‘merely’ a chemical process, but also highly regulated by enzymes and transporters including GPX4, system xCT, ferroptosis suppressor protein 1 (FSP1), acyl-co-enzyme A (CoA) synthetase long chain family member (ACSL) 4, and many others, provided key mechanistic insight as well as actionable drug targets [39]. Third, the identification of ferroptosis-specific small molecule inducers and inhibitors enabled this mechanism to be isolated from other forms of cell death with high confidence, surely contributing to the rapid growth of this field. Fourth, the connections between ferroptosis and disease – already latent in early studies of lipid peroxidation – were clarified and revisited with new and more chemical tools and concepts at hand. Collectively, this process of (re-)discovery and refinement has allowed the field of ferroptosis research to flourish and expand in many directions, as described next.

### 1.2 Redox metabolism at the crossroads of regulated necrosis

Cell death can be classified as either regulated or spontaneous. Regulated cell death involves a genetically encoded molecular machinery and a precise sequence of events starting from an extracellular or intracellular inducing signal [40]. A dividing line in the field was the identification of genes required for apoptosis, the prototype form of programmed cell death. Besides apoptosis, three decades of cell death research revealed that regulated necrosis is also molecularly orchestrated and crucial in various physiological and pathological contexts. To date, different types of regulated necrosis have been characterized such as necroptosis, parthanatos, mitochondrial permeability transition-driven necrosis, pyroptosis, NETosis, and ferroptosis [41,42]. Ferroptosis constitutes a necrotic cell death pathway resulting from insufficient cellular reduction capacity [43]. The lack of a proactive signaling complex, such as the apoptosome, necrosome, or inflammasome which initiates apoptosis, necroptosis or pyroptosis, respectively, leaves ferroptosis an outsider in the family of regulated necrosis pathways. Alongside Fenton-type chemistry, some enzymes (e.g. lipoxygenases and P450 oxidoreductase) can promote lipid peroxidation within cellular membranes, making ferroptosis a rather primitive mode of regulated necrosis, which seems phylogenetically conserved across all kingdoms of life [44,45]. Apart from these clear differences, each form of regulated necrosis is defined by a 3-step cell death process including initiating, propagating and executioning steps resulting in plasma membrane permeabilization [42] in which the dysregulation of the cellular redox metabolome acts as a common denominator.

**Fig. 2:**
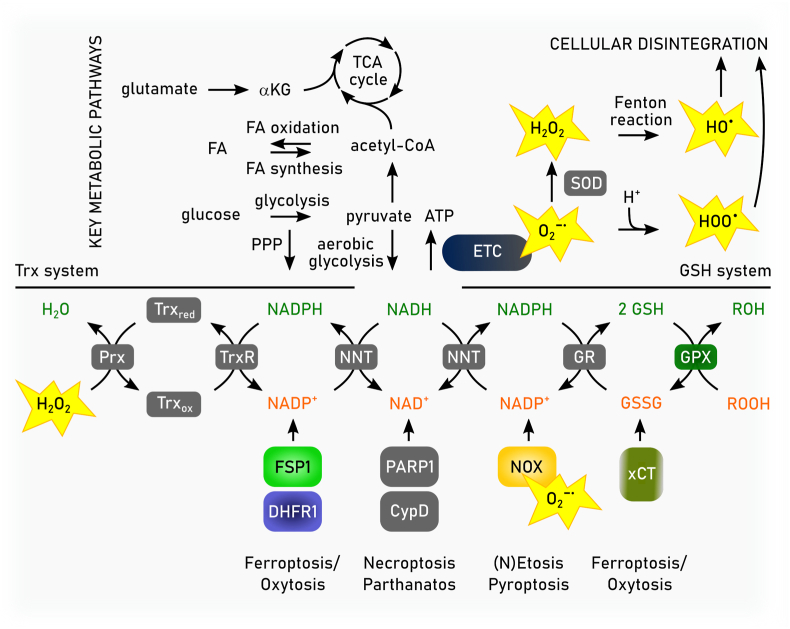
**Deregulated redox metabolome drives regulated necrosis**. The two key thiol-dependent antioxidant systems in mammalian cells, the thioredoxin (Trx) and the glutathione (GSH) redox cycles are dependent on the presence of NAD(P)H cofactor that is restored by nicotinamide nucleotide transhydrogenase (NNT) within the mitochondria. Trx reductase (TrxR) utilizes NADPH to reduce Trx and other low molecular weight compounds. Peroxiredoxins (Prxs), on the other hand, accept electrons from Trx and subsequently scavenges hydrogen peroxide (H_2_O_2_) and other organic hydroperoxides. Like TrxR glutathione reductase (GSR) uses electrons from NADPH to restore reduced GSH from its oxidized state (GSSG). GSH in turn is the main substrate of glutathione peroxidases (GPXs). Members of the GPX protein family convert hydrogen peroxide and lipid hydroperoxides into water and alcohols, respectively. Different redox couples are affected by different types of regulated necrosis. Ac-CoA: Acetyl-CoA, αKG: α-ketoglutarate, DHFR1: dihydrofolate reductase 1, FA: fatty aci, NAD: nicotinamide adenine dinucleotide, NADPH: nicotinamide adenine dinucleotide phosphate, Prx: peroxiredoxin, SOD: superoxide dismutase.

The redox metabolome refers to the collective set of molecules involved in reduction-oxidation reactions within cells, such as NAD(P)H/NAD(P)^+^, GSH/GSSG, cysteine/cystine. During redox reactions electrons from reduced donor molecules are transferred to oxidized acceptor molecules [46]. These redox reactions, which are involved in many cellular signaling events, energy production and responses to oxidative stress, are intertwined with key metabolic pathways [47] (Fig. 2). In the context of the redox metabolome, the electron transport chain (ETC), involved in the metabolic process of oxidative phosphorylation (OXPHOS), serves as a primary source for complex I-derived superoxide radical anion, which is subsequently converted to hydrogen peroxide by superoxide dismutase (SOD), leading to hydroxyl radicals in the presence of Fe^2+^ [47]. At the plasma membrane and other subcellular sites the formation of superoxide anion is catalyzed by NADPH oxidases (NOX). While these reactive oxygen species (ROS) have essential roles in signaling and host defense, supraphysiological levels of ROS and/or impaired antioxidant defenses may causes oxidative damage to DNA, proteins, and lipids, resulting in mitochondrial dysfunction and even cell death [47].

The two key thiol-dependent antioxidant systems in mammalian cells consist of thioredoxin (Trx) and the GSH redox cycles [46]. These redox systems rely on NADPH as their primary electron source, placing NADPH as a central hub in the maintenance of cellular redox balance (Fig. 2). In the cytosol, NADPH is primarily produced through the pentose phosphate pathway (PPP), while mitochondrial NADPH is generated by enzymes such as isocitrate dehydrogenase (IDH) and nicotinamide nucleotide transhydrogenase [46]. The glycolysis pathway in the cytosol and the tricarboxylic acid (TCA) cycle in mitochondria also contribute to the redox balance by generating NADH, which can be converted to NADPH [48]. In various forms of regulated necrosis, cellular demise upon increased oxidative damage is associated with either decreased NAD(P)H and/or GSH levels or by increased levels of reactive species [42] (Fig. 2).

How is redox metabolism linked to different cell death modes by regulated necrosis? Cell death due to reduction in GSH biosynthesis was originally coined oxytosis [43], and later renamed to ferroptosis [5]. The levels of NADPH are an important determinant in ferroptosis sensitivity across different cancer cell lines [49]. It is an essential cofactor for reducing oxidized GSH (GSSG) [28], for the regeneration of reduced co-enzyme Q_10_ (CoQ_10_) and vitamin KH_2_ by FSP1 [[50], [51], [52]], and the biosynthesis of the potent RTA tetrahydrobiopterin (BH4) via dihydrofolate reductase (DHFR) [53,54]. However, the redox metabolome may not only affect ferroptotic cell death, as increasing evidence also suggests an implication in necroptosis [42,55]. Besides serving as a source of reducing equivalents for the key cellular antioxidant defense systems, NADPH is the principal substrate of NOX [56]. NOX1-mediated superoxide formation contributes to necroptosis through its recruitment to tumor necrosis factor (TNF) receptor 1 by riboflavin kinase [57,58]. Necroptosis is associated with increased glycogenolysis, glycolysis, and glutaminolysis following receptor-interacting protein kinase (RIPK) 3 activation, resulting in enhanced NADH production [59]. Increased levels of NADH may induce an overstrained ETC, particularly involving NADH dehydrogenase (mitochondrial complex I of ETC), ultimately leading to enhanced electron leakage and superoxide anion production. Although mitochondrial ROS seems to be dispensable for the eventual execution of necroptosis [60], its inhibition retards its progression [61,62]. In addition to ferroptosis and necroptosis, other modes of regulated necrosis might also be affected by the cellular redox metabolome. Parthanatos is activated in response to DNA damage induced by factors such as UV light, ROS, or alkylating agents, along with an increase in Ca^2+^ concentration. In parthanatos, the PARylation of proteins due to overactivation of Poly ADP ribose polymerase 1 leads to NAD^+^ exhaustion and consequently impaired ATP generation [63,64]. Mitochondrial permeability transition-driven necrosis is characterized by cyclophilin D-mediated opening of the permeability transition pore, which is induced by excessive production of ROS and Ca^2+^ overload of the mitochondrial matrix [65]. Suicidal NETosis also involves a burst of ROS production, which might involve NOX (depending on the involved pathogen) to kill microbial invaders [66]. Increased levels of ROS also plays a pivotal role at the initiation and regulation of inflammasome pathways [67,68]. Caspase 1 activation by the NLRP3 inflammasome is regulated by NOX2 to control phagosome function [69]. Moreover, hydrogen peroxide was shown to sensitize to pyroptosis by oxidation of C192 of gasdermin D, leading to enhanced oligomerization and pore formation downstream of the inflammasome-mediated caspase activation [70].

Altogether, these observations suggest that cellular redox metabolism may affect different forms of regulated necrosis beyond ferroptosis. According to this concept, dysregulation of the redox metabolome leads to diminished antioxidant capacity, increased levels of ROS, energy crisis, organelle dysfunction or rupture, and cellular disintegration [42,71]. The diversity in the molecular initiation mechanisms governing regulated necrosis suggests that targeting redox mediators may offer an attractive broad-spectrum approach of inhibiting regulated necrosis.

## 2 Key players and processes in ferroptosis

### 2.1 Protective enzymes

#### 2.1.1 Glutathione peroxidase 4

Discovered by Ursini and coworkers as a phospholipid hydroperoxide reducing enzyme using GSH as cofactor a little more than 40 years ago, GPX4 was initially named as phospholipid hydroperoxide GSH peroxidase, the second GSH peroxidase to be identified in mammals [21,22]. Meanwhile, it has become clear that GPX4 is one of eight distinct GSH peroxidases in mammals harboring the 21st proteinogenic amino acid selenocysteine in its active site. Intriguingly, selenocysteine in GPX4 can be replaced by its functional homolog cysteine as homozygous cysteine-expressing mice survive up until the preweaning stage (when kept on a mixed C57BL/6j x 129Sv genetic background) in stark contrast to constitutive *Gpx4*^*−/−*^ mice as detailed further below. However, homozygous cysteine-GPX4 expressing mice must be sacrificed at the preweaning stage due to the development of severe epileptic seizures due to the loss-of a distinct subpopulation of GABAergic inhibitory interneurons [72]. Owing to the intrinsically higher sensitivity of the thiol group towards peroxide-induced irreversible overoxidation as compared to the selenol group, this “hypomorphic allele”, when crossed on a conditional loxP-flanked *Gpx4* allele, has become a valuable sentinel mouse line to study the contribution of GPX4 in pathological scenarios that have been linked to ferroptosis [73].

**Fig. 3:**
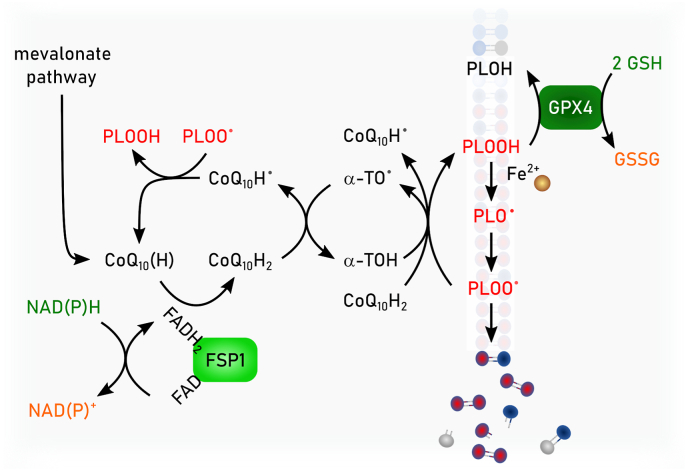
**Enzymatic reactivities of GPX4 and FSP1.** Glutathione peroxidase 4 (GPX4) reduces in a glutathione (GSH)-dependent reaction phospholipid hydroperoxides (PLOOH) to phospholipid alcohols (PLOH), whereas ferroptosis suppressor protein 1 (FSP1) convert phospholipid peroxyl radicals (PLOO^•^) to PLOOH. NAD(P)H reduces the flavin adenine dinucleotide (FAD) bound to FSP1. Via this reduced FAD (FADH_2_) FSP1 reduces quinone (CoQ10(H)) to hydroquinone (CoQ10H_2_). CoQ10H2 promotes the reaction from PLOO^•^ to PLOOH either by its own reaction with PLOO• or by restoring α-tocopherol (α-TO) facilitating the same reaction. Semihydroquinone (CoQ10H^•^) reacts with another PLOO^•^ to form again CoQ10(H).

Phylogenetically, monomeric GPX4 is structurally related to the endoplasmic reticulum (ER)-resident GPX7 and GPX8, although the latter two contain cysteine in their active sites. In humans, five out of eight GPX (i.e., GPX1-4 and GPX6) express selenocysteines in their active site, whereas in rodents GPX6 is a cysteine-containing ortholog. Among the GPX family of proteins, GPX4 is unique as it is the sole enzyme reducing peroxidized acyl chains in PLs and cholesterol hydroperoxides to their corresponding alcohols using GSH and even other low molecular thiols and protein thiols, although the latter might be restricted to its moonlighting function in sperm development as outlined below [74]. Since (phospho)lipid peroxidation has been established to drive ferroptosis [39], it is this enzymatic activity which accounts for the GPX4's principal role in ferroptosis surveillance [6,24,28], and which renders GPX4 the guardian of ferroptosis (Fig. 3). Besides its role in the detoxification of hydroperoxides in PLs and cholesterol, GPX4 is also involved in oxylipin signaling as it governs the activities of the arachidonic acid metabolizing enzymes lipoxygenase and cyclooxygenase [75]. Since enzymes of the lip- and cyclooxygenase protein family carry iron, either as non-heme iron or in form of heme, respectively, this needs first to be oxidized (i.e., “activated”) by a lipid hydroperoxide to allow hydrogen abstraction from a bisallylic methylene carbon of a poly unsaturated fatty acid (PUFA) substrate to give a carbon-centered radical. This then combines with molecular oxygen to form a peroxyl radical, which upon reduction and protonation, yields the monohydroperoxy PUFA product (e.g., 15S-hydroperoxy-5Z,8Z,11Z,13E-eicosatetraenoic acid). As the catalytic iron of both classes of enzymes is oxidized by a lipid hydroperoxides, the lipoxygenase and cyclooxygenase are therefore deemed to be under indirect control of GPX4.

GPX4 is expressed in three distinct forms arising from one gene that consists of eight exons [76]. While the short form (around 19 kD), also known as either “cytosolic” or “somatic” form, is ubiquitously expressed in most tissues and cell lines, expression of the mitochondrial form (mGPX4) with a cognate mitochondrial targeting site at its N-terminus is almost exclusively restricted to male germ cells, i.e. spermatocytes. Similarly, expression of the nuclear form (initially called sperm-nuclei specific form, nGPX4) with a long N-terminal extension including a nuclear localization signal and DNA binding domains resembling those present in protamines, is constrained to testis, specifically in late spermatids [77]. Although GPX4 might be regarded as a housekeeping selenoenzyme, still relatively little is known about its regulation, either on the transcriptional, translational or stability level, particularly under pathological conditions. While an approimately 200 base pair region upstream of the translational initiation site of the cytosolic isoform (3′-ATG) of the murine *Gpx4* gene contains binding sites for stimulating protein 1/3, nuclear factor Y and members of the SMAD family [78], regulation of *Gpx4* by the oxidative stress sensing Kelch-like ECH-associated protein 1-(KEAP1)/nuclear factor-erythroid 2-related factor 2 (Nrf2) system remains strongly debated and also less likely due to the lack of related structural *cis*-elements in its promoter region. Expression of *mGpx4* was further reported to be regulated on the post-transcriptional level by guanine-rich sequence-binding factor 1 (GRFS1) that binds to a sequence in the 5′-untranslated region of *mGPx4* mRNA, thereby upregulating mGPX4 translation [79]. This GRFS1-mediated upregulation is deemed important for embryonic brain development, as siRNA-mediated knockdown of GRFS1 suppresses GPX4 expression causing developmental aberrations, particularly in the brain [79]. However, since mice with a specific knockout of the mitochondrial isoform of GPX4 develop normally and are fully viable (see further below), such a mechanism needs to be carefully considered. Recently, GPX4 was also shown to be subject to regulation on the post-translational level [80] (see also chapter 3.3). Downstream of insulin-like growth factor 1 receptor and serine/threonine kinase (AKT) signalling, phosphorylation of creatine kinase B (CKB) on T133 by AKT shifts its canonical metabolic activity and facilitates binding and phosphorylation of GPX4 on S104. S104 phosphorylation in turn abrogates binding of heat shock cognate 71 kDa protein to GPX4 which causes stabilization of GPX4 due to impaired chaperone-mediated autophagy. Notably, the levels of CKB T133 and GPX4 S104 phosphorylation have been associated with poor prognosis of patients with hepatocellular carcinoma, thus linking ferroptosis evasion to the non-metabolic moonlighting function of CKB [80]. Yet, the finding that GPX4 becomes phosphorylated is not entirely new, as earlier studies showed that GPX4 is tyrosine-phosphorylated during hamster sperm capacitation [81].

Gene knockout studies in mice have been instrumental in providing essential insights into the *in vivo* relevance of GPX4 function - and hence ferroptosis - in tissue homeostasis and protection. The first report using constitutive knockout of the *Gpx4* gene demonstrated its indispensability for early embryonic development as knockout embryos die at the gastrulation stage (E7.5), for yet-unknown reasons [23]. By contrast, the targeted knockout of either the nuclear or the mitochondrial isoforms has no impact on embryo development, and adult mice present only specific defects in sperm development with impaired chromatin condensation in *nGpx4*^*−/−*^ mice and impaired formation of the mitochondrial capsule and male infertility of *mGpx4*^*−/−*^ mice. Due to its moonlighting function, whereby GPX4 converts from a phospholipid hydroperoxidase to a thiol peroxidase due to the absence of sufficient concentrations of cellular GSH (as physiologically evident in developing sperm), GPX4 introduces disulfide bridges into mitochondrial capsular proteins in the midpiece of spermatozoa or into protamines, which replace the majority of histones essential for sperm compaction in spermatids [82,83]. An elegant study by Liang et al. using transgenic mice expressing either the short/cytosolic or the mitochondrial forms demonstrated that only transgenic expression of the cytosolic form rescues embryonic lethality of *Gpx4*^*−/−*^ embryos and allows survival of adult mice (male mice present with sterility due to lack of expression of mGPX4), corroborating that it is the short/cytosolic form that is essential for protecting cells and tissues from ferroptosis [84]. Notably, it was recently reported that mGPX4 is abundantly expressed in photoreceptor cells and that the loss-of mGPX4 causes rapid degeneration of cone-rod photoreceptors [85]. Additional studies are therefore warranted to show whether mGPX4 may confer specific functions beyond those in sperm development and photoreceptor viability. Nonetheless, cellular studies, where an important anti-ferroptotic function of mGPX4 was postulated in somatic cell including cancer cell lines, must be interpreted with care as overexpression of mGPX4 in somatic cells on a GPX4 knockout background may cause improper/insufficient mitochondrial matrix localization, thereby allowing survival of *Gpx4*-deleted cells.

Due to the early embryonic lethal phenotype of GPX4 null mice, a series of tissue-specific knockout approaches have been performed in the past. The first report on a conditional *Gpx4* allele (loxP-flanked, floxed) was by Seiler et al., who provided early proof that neuron-specific ablation of *Gpx4* causes ataxia, neuronal loss in cortex and hippocampus, as well as early death of mice [24]. This mouse model furthermore laid the foundation for generating a cellular system harboring two floxed *Gpx4* alleles and stably expressing 4-hydroxy-tamoxifen-inducible mouse embryonic fibroblast, now generally known as “Pfa1 cells”. In these cells, tamoxifen-induced knockout of *Gpx4* is sufficient to cause lipid peroxidation and a “non-apoptotic form of cell death”, which is prevented by vitamin E and some “lipoxygenase-specific” inhibitors [24]. The initial observation that 12/15-lipoxygenase may play a role in the cell death process downstream of GPX4 was, however, refuted by subsequent double knockout studies in mice showing that 12/15-lipoxygenase encoding *Alox15* loss fails to rescue kidney failure [6], T cell development and homeostasis [86] and even embryogenesis [87]. Meanwhile, we know that GPX4 is not only essential for certain types of neurons, such as glutamatergic and GABAergic neurons in the cortex, cerebellar Purkinje cells and motor neurons, but also for the development/homeostasis of to various cell types and tissues outside the brain, such as renal proximal tubular epithelial cells [6], certain types of T cells [86], spermatogonial stem cells [88], B1 and marginal zone B cells [89], neutrophils [90] and reticulocytes [91]. Phenotypes induced by loss of GPX4 can be compensated by dietary vitamin E levels depending on the organ (see chapter 2.2.2). These studies indicate that besides the general health status and microbiome makeup of mice, it is essential to carefully consider the vitamin E content of the chow. Evidently, this does not only apply to *in vivo* studies using tissue-specific ablation of GPX4, but also to other ferroptosis-related disease models, such as tissue ischemia/reperfusion injury, intoxication, neurodegeneration and cancer.

#### 2.1.2 Ferroptosis suppressor protein 1

FSP1 (also known as apoptosis inducing factor mitochondria associated (AIFM) 2) was discovered as a ferroptosis resistance factor and CoQ_10_ oxidoreductase in 2019 by two groups employing complementary genetic screening strategies [50,51]. N-terminal myristoylation of FSP1 is required for its association with cellular membranes, including the plasma membrane and lipid droplets, and is essential for ferroptosis suppression [50,51]. Targeting FSP1 selectively to the plasma membrane is sufficient to inhibit ferroptosis [50], in accordance with the importance of maintaining plasma membrane integrity and with the plasma membrane as the primary site of FSP1 action in ferroptosis resistance.

The mechanisms that enable FSP1 to localize to specific membranes and whether there is dynamic exchange between organelles remain unclear. Mechanistically, FSP1 functions as an NAD(P)H-dependent oxidoreductase that re-generates lipophilic RTAs, which reduce lipid-derived peroxyl radicals to inhibit the propagation of lipid peroxidation [[50], [51], [52],^92^]. The first substrate of FSP1 to be identified was CoQ_10_ [50,52]. Purified FSP1 reduces oxidized CoQ_10_ (i.e., ubiquinone) to its hydroquinone form (i.e., ubiquinol) at the expense of NAD(P)H *in vitro* [50,52] (Fig. 3). Moreover, expression of FSP1 in cells, but not a catalytically inactive mutant, increases the ratio of ubiquinol to ubiquinone [50,52]. The final steps of CoQ_10_ synthesis occur in the mitochondria, where CoQ_10_ plays its most well-known role in moving electrons within the electron transport chain. The discovery of FSP1 revealed a mechanism to recycle non-mitochondrial pools of CoQ_10_, locally providing a powerful antioxidant to prevent membrane lipid peroxidation. The source and trafficking of non-mitochondrial pools of CoQ_10_ remain poorly understood. StAR Related Lipid Transfer Domain Containing 7 (STARD7) provides a piece to this puzzle, with a proteolytically processed form of STARD7 functioning as a cytosolic CoQ_10_ binding protein that mediates CoQ_10_ transport from mitochondria to the plasma membrane [93]. It has been proposed that a portion of CoQ_10_ biosynthesis takes place outside of the mitochondria mediated by the ER and Golgi enzyme UbiA prenyltransferase domain containing 1 (UBIAD1) [94], though it remains possible that UBIAD1 influences CoQ_10_ synthesis indirectly via its regulation of the mevalonate pathway. CoQ_10_ can also be obtained from circulating lipoproteins, but similar to *de novo* synthesized CoQ_10_, the intracellular trafficking of CoQ_10_ obtained from lipoproteins is not well understood. A potential role for cluster of differentiation (CD) 36 has been demonstrated in brown adipocytes [95]. Consistent with the major role of CoQ_10_ in FSP1-dependent ferroptosis suppression, inhibition of *de novo* CoQ_10_ synthesis sensitizes cells to ferroptosis and deletion of *FSP1* under these depleted CoQ_10_ conditions does not result in additional sensitization [50,51]. FSP1 is able to reduce additional RTAs including vitamin K, vitamin E, and other quinone-containing substrates [[50], [51], [52],^92^]. The discovery that FSP1 reduces vitamin K solved a long standing mystery, identifying the warfarin-resistant oxidoreductase that reduces vitamin K to combat warfarin poisoning [52]. These additional quinones may also contribute to the FSP1 ferroptosis resistance mechanism. The precise contribution of different FSP1 substrates remains to be quantified and may vary depending on cell type or state. Together, the emerging findings support a model in which FSP1 acts at specific membranes to locally recycle RTAs, such as reduced CoQ_10_ and vitamin K, to suppress lipid peroxidation.

The crystal structures of non-myristoylated human (amino acids 10–373) [96] and chicken FSP1 (amino acids 12–373) [97] indicate that FSP1 contains three domains, a FAD-binding domain, a NAD(P)H binding domain, and a C-terminal substrate binding domain. The human FSP1 structure was solved in complex with 6-OH-FAD and NADP+ [96], and the chicken FSP1 structure was solved in complex with FAD and in the presence and absence of CoQ_10_ [97]. There were some notable differences, although crystals might not reflect the situation in solution: human FSP1 was crystalized as a monomer [96], whereas chicken FSP1 was crystalized as a homo-dimer [97]. Purified human FSP1 was observed as both a monomer and dimer by dynamic light scattering and Myc-tagged human FSP1 co-immunopurified with FLAG-tagged FSP1 isolated from cells [97], raising the possibility that FSP1 functions as a dimer similarly to some other oxidoreductases, e.g. AIFM1 or yeast and bacterial NADH-ubiquinone reductases [97]. However, whether human FSP1 acts as a dimer is contentious [96] and remains to be determined. Although FSP1 is capable of using NADH and NADPH to reduce its substrates *in vitro*, it was observed that human FSP1 bound ∼80 fold more tightly to NADPH and structure-guided mutational analyses suggested that FSP1 preferentially employs NADPH in cells [96]. Interestingly, a loop within the chicken FSP1 C-terminal substrate binding domain adopted distinct conformations in the substrate-free and CoQ_10_-bound structures, implying a potential role for this loop in regulating substrate access to the FSP1 active site [97]. Additional studies will be necessary to fully understand the biochemistry and the exact catalytic cascade of FSP1. However, the emerging data are consistent with an expected biochemical mechanism in which FSP1 first employs NAD(P)H to reduce FAD to FADH_2_ (or 6-OH-FAD to 6-OH-FADH_2_), and then two electrons are transferred from FADH_2_ to the substrate (e.g., CoQ_10_).

*FSP1* expression strongly correlates with the resistance of cancer cell lines to ferroptosis induced by GPX4 inhibitors [50,51] and *FSP1* expression is amplified in some cancers [98]. For example, *FSP1* is a target of the transcription factor NRF2 [98]. Indeed, *FSP1* is highly expressed in KEAP1 mutant lung cancers, which have high NRF2 levels due to impaired proteasomal clearance and are resistant to GPX4 inhibition [98]. Knockout (KO) of *FSP1* strongly sensitizes KEAP1 mutant lung cancer cells to ferroptosis [50,98] and shRNA-depletion of FSP1 is sufficient to suppress tumor growth in a KEAP1-deficient lung cancer xenograft model [98]. Recently developed small molecule FSP1 inhibitors (see also chapter 3.6), including iFSP1 [51], FSEN1 [99], icFSP1 [100], and viFSP1 [101] sensitize a variety of cancer cell lines with different tissue origins and oncogenic driver mutations to ferroptosis. Most of these FSP1 inhibitors are specific for human FSP1 [51,[99], [100], [101], [102]], whereas the recently identified viFSP1 is a multi-species FSP1 inhibitor. However, due to insufficient metabolic stability of viFSP1 in liver microsomes, there is a continuing need for inhibitors of mouse FSP1 to enable *in vivo* studies. Several additional, structurally distinct classes of FSP1 inhibitors were described [99], but whether these are effective against mouse FSP1 has not been tested. A major question that remains to be addressed is the therapeutic value of FSP1 as a cancer treatment target. *FSP1* gene disruption [50,52] or chemical inhibition [51,[98], [99], [100]] sensitizes cancer cells to other ferroptosis inducers (e.g., GPX4 inhibitors or endoperoxide-containing ferroptosis inducers), but FSP1 inhibition is typically not sufficient on its own to induce ferroptosis in cultured cancer cells [50,51,[98], [99], [100]]. However, depletion of *FSP1* alone was sufficient to suppress tumor growth in a xenograft model of lung cancer [98], raising the possibility that treatment with only FSP1 inhibitors may have therapeutic value for certain cancers *in vivo*. It is worth noting that *FSP1* KO mice are viable and do not exhibit overt tissue degeneration [52,103,104]. The physiological function of FSP1 is poorly understood, but FSP1 has been found to support glycolytic flux in adipose [104] and exercising muscle [105], thereby promoting cold- and diet-induced thermogenesis and aerobic exercise. The relatively mild phenotypes in the *FSP1* KO mice [103,104] suggest that FSP1 inhibition will be well tolerated in humans. This is in contrast to the more severe phenotypes following the loss of *Gpx4* in mice, such as embryonic lethality in *Gpx4* KO and tissue degeneration following inducible *Gpx4* KO [6]. FSP1 could be targeted on its own or in combination with additional ferroptosis inducers or with standard of care treatments. It is possible that FSP1 inhibition will sensitize certain tissues to ferroptosis inducers. Indeed, loss of *FSP1* sensitizes mice to kidney ischemia-reperfusion injury [73]. Whether FSP1 functions in other tissues to suppress degeneration remains mostly unexplored. Studies of FSP1 regulation and FSP1 inhibition in preclinical models of cancer and degenerative disease remain high priorities for the field.

### 2.2 Metabolism

#### 2.2.1 Lipids

**Fig. 4:**
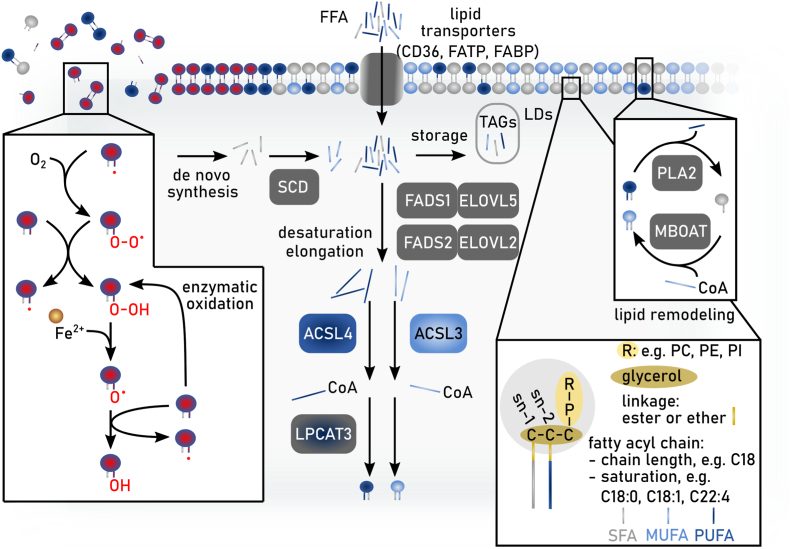
**Lipid metabolism and ferroptosis.** Schematic overview of the main pathways involved in fatty acid biosynthesis and uptake, lipid synthesis and lipid remodeling that regulate ferroptosis sensitivity in cells. In general, two fatty acyl chains are linked to a glycerol backbone via an ester or ether bond. The fatty acids at the sn-1 position are generally saturated fatty acids (SFAs) or mono-unsaturated fatty acids (MUFAs), whereas the fatty acids at the sn-2 position can be SFAs, MUFAs or poly-unsaturated (PUFAs). Lipids attached to the glycerol can be e.g. phosphatidylcholine (PC), phosphatidylethanolamine (PE), or phosphatidylinositol (PI). Free fatty acids (FFA) from the microenvironment can be taken up via CD36, fatty acid transport proteins (FATP) or fatty acid-binding proteins (FABP). The intracellular pool of fatty acids is comprised of SFA as product of *de novo* fatty acid synthesis or uptake, together with either MUFAs or PUFAs as products of fatty acid desaturation and elongation. Noteworthy, certain PUFAs are essential and thus have to be taken up by aforementioned extracellular space. Depending on substrate specificity, fatty acids are desaturated by stearoyl-CoA desaturase (SCD) and fatty acid desaturases (FADS1 or FADS2). Further, different elongases e.g. ELOVL2 or ELOVL5 are synthesizing fatty acids (SFA, MUFA or PUFA) with longer carbon chains. Members of the acyl-CoA synthetase long chain family of proteins (e.g. ACSL3 or ACSL4) convert free long-chain fatty acids into fatty acyl-CoA esters that then can be utilized to become incorporated into membrane phospholipids (PL) mediated by e.g. lysophosphatidylcholine acyltransferase LPCAT3. Additionally, intracellular fatty acids can be stored into lipid droplets (LDs) as they are converted into triacylglyceride. Existing membrane phospholipids can be remodeled via the Lands Cycle. Here, hydrolysis of the fatty acid (e.g. PUFA) at the sn-2 position by PLA2 produces a lyso-PL and a following re-esterification of another fatty acid-CoA (e.g. MUFA) mediated by MBOAT1 leads to the remodeled membrane PL.

Alterations of the lipidome are key determinants of cellular sensitivity towards ferroptosis that are reflected in disease states and cellular development. The major ferroptosis-liable lipid structures are (glycero-) PLs, in which two fatty aciyl chains are linked to a glycerol backbone either as an ester or ether, and which also contain a variable head group. The fatty acids at the sn-1 position are generally saturated or monounsaturated, whereas the fatty acids at the sn-2 position can be fully saturated, mono- or polyunsaturated, determined by the number of carbon-carbon double bonds in the acyl chain; of note, a minor species of diPUFA PLs with PUFA tails at both the sn-1 and sn-2 posotions, was recently reported to be a key contributor to ferroptosis (Fig. 4) [106]. Of the variety of fatty acids found in PLs, PUFAs are most susceptible to autoxidation, the free radical chain reaction that converts lipids to lipid hydroperoxides (lipid peroxidation). For homoconjugated PUFAs such as arachidonic, α-linolenic and linoleic acids, propagation of the chain reaction involves abstraction of a bis-allylic H-atom by a lipid-derived peroxyl radical (LOO^•^), which yields a lipid hydroperoxide (LOOH) and resonance-stabilized lipid radical (L^•^) that rapidly reacts with O_2_ to yield a new peroxyl radical [107,108]. The initiation of lipid peroxidation is largely driven by non-enzymatic Fenton-type reactions of product LOOH with ferrous iron (Fe^2+^), producing highly reactive lipid-derived alkoxyl radicals (LO^•^) that can abstract H-atoms from lipids. Since the products of lipid peroxidation (i.e. LOOH) can initiate new chain reactions, these chain reactions are said to be auto-initiated and proceed at ever increasing rates. Several enzyme classes may also contribute to the initiation of lipid peroxidation. For example, lipoxygenases contribute to the pool of LOOH via their metabolism of arachidonic acid to corresponding hydroperoxyeicosatetraenoic acids, which can engage in Fenton-like reactions to yield lipid peroxidation-initiating LO^•^ [109,110]. Accumulation of lipid peroxidation products and their fragmentation to truncated PLs is believed to underlie the loss of plasma membrane integrity that results in ferroptotic cell death [6]. Therefore, three major factors which determine the susceptibility towards ferroptosis are: i) the modulation of fatty acid diversity and their channeling into PLs resulting in the increase of PUFA-containing membrane lipid species that are prone to lipid peroxidation, ii) the accumulation of labile redox-active iron and the formation of cellular hydroxyl radicals, and iii) the ability to suppress lipid peroxidation via enzymes (e.g. GPX4) or small molecules/metabolites (e.g. RTAs). Factors ii) and iii) are discussed in detail in chapters 2.1, 2.2.2, and 2.2.3. Here, we highlight lipid peroxidation as the central determinant of ferroptosis sensitivity by discussing fatty acid and lipid metabolism with regard to the regulation of PUFA content in membrane PLs.

##### 2.2.1.1 Modulation of fatty acid diversity

Altered fatty acid and lipid metabolism resulting in the remodeling of fatty acids in lipids or a shift to a different processing of lipids can favor a ferroptosis-prone state. The pool of cellular fatty acids that are available for lipid synthesis is sustained via *de novo* fatty acid biosynthesis or the uptake of fatty acids (including essential fatty acids) and lipids from the microenvironment (Fig. 4). In the context of ferroptosis, the relative abundance and uptake of monounsaturated fatty acids (MUFAs), mainly oleic acid (C18:1), from the microenvironment renders cells ferroptosis-resistant once these MUFAs are incorporated into membrane lipids [111,112]. Visa versa, an increased uptake of essential PUFAs, linoleic (C18:2, ω-6) or linolenic acid (C18:2, ω-3), and their subsequent intracellular elongation and desaturation sensitizes cells to lipid peroxidation once these PUFAs are transferred into membrane lipids. Therefore, pathway alterations modulating fatty acid uptake, synthesis and desaturation as well as phospholipid remodeling heavily impact on ferroptosis sensitivity (Fig. 4). Specific processing of essential PUFAs is mediated by elongases, elongation of very long chain fatty acids protein (ELOVL) 2 and ELOVL5, and the fatty acid desaturases (FADS) 1 and FADS2 to produce, among others, arachidonic (C20:4, ω-6) and eicosapentaenoic (C20:4, ω-3) acids that are precursors for production of oxylipins as signalling molecules [113,114]. When arachidonic acid or its elongated product adrenic acid (C22:4, ω-6) are incorporated into PLs such as phosphatidylethanolamines (PE) or phosphatidylcholines (PC), they increase the susceptibility of these PLs to peroxidation and ferroptosis [115,116]. Indeed, by correlating lipidomics data from the Cancer Cell Line Encyclopaedia with essentiality scores derived from whole-genome CRISPR screens (DepMap), researchers found that cells with a high content of PUFA-containing lipids crucially depend on GPX4 expression to sustain viability [117]. By contrast, PUFA-low cells need to maintain high activity of stearoyl-CoA desaturase (SCD), the rate-limiting enzyme for MUFA formation, to avoid accumulation of saturated fatty acids and ER stress, demonstrating an inverse correlation of SCD and GPX4 requirement [117]. Mechanistically, both fatty acid desaturases SCD and FADS2 are transcriptionally controlled by the sterol regulatory element binding protein 1 (SREBP1) downstream of the PI3K/AKT/rapamycin kinase complex 1 (mTORC1) signaling pathway [118]. A recent study shows that activation of SREBP1 by the PI3K/AKT/mTORC1 pathway suppresses ferroptosis by promoting lipogenesis and desaturation [119].

##### 2.2.1.2 Modulating phospholipid diversity

During lipid synthesis, the activation of fatty acids to form acyl-CoA represents the first regulatory step, since different acyl-CoA synthetases display distinct fatty acid substrate selectivity (Fig. 4). More specifically, ACSL4 promotes the incorporation of PUFAs, such as arachidonic (C20:4, ω-6), adrenic (C22:4, ω-6), and docosahexaneoic (C22:6, ω-3) acids into PE lipids [115,116]. Experimentally, it was shown that genetic depletion of ACSL4 renders cells resistant towards ferroptosis induction by inhibition of GPX4, while maintaining a normal proliferative state. Conversely, overexpression of ACSL4 in a panel of breast cancer cells increased lipid peroxidation after GPX4 inhibition [116]. Similarly, the importance of distinct lipid species of plasmalogens (PLs carrying vinyl ether and ester linkages at the sn-1 and sn-2 positions, respectively) for the modulation of ferroptosis susceptibility was described. Again, PUFAs at the sn-2 position within these ether PLs (PUFA-ePLs) are the main species prone to membrane lipid peroxidation. Decreasing the abundance of PUFA-ePLs by downregulating the expression of enzymes regulating synthesis of ePL (e.g. alkylglycerone-phosphate synthases) led to a ferroptosis-resistant state [120]. Another mechanism of ferroptosis-evasion was recently described in metastasizing melanoma cells. During cell transition through the MUFA-enriched (oleic acid, C18:1) lymphatic fluid, these cancer cells gain a substantial survival benefit for the subsequent steps of metastatic dissemination by reducing their ferroptosis-sensitivity [112]. Mechanistically, lipid remodeling by ACSL3 to incorporate oleic acid taken up from the microenvironment into membrane PLs mediates this effect. Together, this establishes the molecular and functional connection between the relative abundance of MUFAs and PUFAs in the microenvironment and the ACSL-dependent channeling of different fatty acids into membrane lipids as defining factors in ferroptosis sensitivity. In addition, membrane PLs are also subject to constant remodeling via the Lands-cycle [121] (Fig. 4). This involves the combined action of phospholipases, lysophosphatidylcholine acyltransferase (LPCAT) or membrane bound O-acyl transferases (MBOATs) that, depending on the acyl-CoA substrate, can either promote or dampen the susceptibility towards lipid peroxidation [[122], [123], [124]]. It was recently shown that the uncommon lipid class of PLs with PUFA tails in both sn-1 and sn-2 positions underlie rapid lipid remodeling. Free fatty acid treatment led to significant changes in PC-PUFA_2s_ levels rather than their PUFA_1s_-conjugated counterparts but had only minor effects on PC-PUFA_2s_-induced lathality implicating them as proximal mediators of ferroptosis [106].

##### 2.2.1.3 Deciphering the biological necessity and consequences of PUFA metabolism

The membrane PL composition as driver for ferroptosis susceptibility can be pre-determined based on the innate lipidome within the tissue or cell of origin. In the brain, for example, unesterified PUFAs, most prominently arachidonic and docohexaenoic acid, serve as precursors for lipid mediators, such as prostaglandins and resolvin Ds, important for balancing pro-vs. anti-inflammatory responses [125]. High PUFA content in a membrane is also described to increase its bending and deformation ability, a feature important for facilitating endocytosis and compartmentalization processes, for example in spermatids or entero- and hepatocytes [126]. Besides cell lineage-intrinsic preferences for lipid subspecies, changes in membrane lipid composition can be acquired as part of cellular plasticity programs. It has been reported that cells with a mesenchymal phenotype, often featured in highly malignant and drug-tolerant cancer cells, exhibit higher incorporation of PUFA into PLs due to an increased expression of long chain PUFA-generating enzymes [114]. This links changes in lipid metabolism to the previously reported increased ferroptosis sensitivity in highly undifferentiated cells [127,128]. Functionally, favoring PUFA incorporation into plasma membranes might also contribute to higher cell mobility, a central requirement during embryogenic development, wound healing or metastasis formation. Mechanistically, the transcription factor zinc finger E-box binding homeobox 1 (ZEB1), a master regulator of the cellular plasticity program ‘epithelial-to-mesenchymal transition’, was identified to fine-tune phospholipid composition via direct transcriptional activation of long chain PUFA-generating enzymes FADS2 and ELOVL5, while simultaneously repressing enzymes regulating MUFA/saturated fatty acid (SFA) production. This is likely one mechanism explaining the higher ferroptosis sensitivity of cells in a mesenchymal state [127]. Another relevant example is provided for clear cell carcinoma, where a selective enrichment of PUFAs into triacylgylcerides and PLs is driven by the activity of hypoxia-inducible factor 2α and the downstream, hypoxia-inducible lipid droplet-associated protein [129], important for excess lipid storage. Fittingly, several reports have shown that inducing lipophagy, a specialized form of lipid degradation, sensitizes cells to ferroptosis [130], possibly by releasing PUFAs for subsequent incorporation into membrane PLs. Ultimately, the necessity for high PUFA abundance in PLs comes at the expense of increased susceptibility towards lipid peroxidation and consequently the dependency on detoxification pathways, thus creating unique targeting opportunities to exploit this vulnerability in various diseases, including cancer [131].

##### 2.2.1.4 Acyl-CoA synthetase long chain family members and lysophospholipid acyltransferases in ferroptosis

There are more than twenty mammalian acyl-coenzyme A synthetase enzymes. These enzymes use ATP and CoA to ‘activate’ fatty acids to fatty acyl-CoAs in a two-step reaction [132]. Fatty acid activation is required for fatty acyl-CoAs to be further metabolized and/or incorporated into more complex lipids such as triglycerides and PLs. In relation to ferroptosis, the five ACSLs have received the greatest attention. Links between ACSL enzymes and ferroptosis regulation first emerged from genetic studies, which identified *ACSL4* as being essential for the execution of ferroptosis in response to covalent GPX4 inhibitors such as RSL3 and ML162 [116,133]. Indeed, across two dozen different ferroptosis-related genetic screens, *ACSL4* is the most frequently identified suppressor gene [134], whereby loss-of-function of ACSL4 is usually associated with decreased ferroptosis sensitivity. Mechanistically, this is explained by the fact that ACSL4 preferentially activates PUFAs, including C20:4 and C22:4, that are incorporated into membrane PLs [115,135]. These PUFA-containing PLs are the key substrates of lethal lipid peroxidation following inactivation of key anti-ferroptotic proteins such as GPX4.

*ACSL4* mRNA expression can be upregulated in certain cancer cells and thereby contribute to increased ferroptosis sensitivity [136]. The activity of ACSL4 can also be regulated post-translationally by protein kinase CβII (PKCβII). PKCβII phosphorylates ACSL4 at threonine 328 to increase the activity of this enzyme, boost the levels of PUFA-containing PLs, and thereby favor the execution of ferroptosis [137]. The activity of PKCβII itself appears to be stimulated by lipid peroxidation, implying the existence of a pro-ferroptotic positive feedback mechanism. This mechanism may be necessary to overcome the anti-ferroptotic effect of oxidized PUFA cleavage by phospholipases from membrane PLs [138]. Whether ACSL4 plays a universal or context-specific role in ferroptosis has recently been questioned. While ACSL4 appears essential for ferroptosis in response to direct GPX4 inhibition, ferroptosis, as defined by the RTA-sensitive inhibition of lipid peroxidation and cell death, can be activated in cancer cells lacking ACSL4 by p53 expression [139], photodynamic therapy [140], and even system xCT inhibition [135]. One possibility is that different ferroptosis-inducing conditions lead to the peroxidation of distinct PUFA-containing PLs, which in turn are the products of different ACS enzymes, such that ferroptosis in response to one lethal stimulus can be fully or only partially dependent upon ACSL4.

ACSL4 is not the only ACSL enzyme linked to ferroptosis regulation. ACSL1 is needed for ferroptosis in cancer cells fed the conjugated PUFA α-eleostearic acid (αESA) [141]. Here, ACSL1 activity is required for αESA incorporation into triacylglycerols, which is an essential step in the lethal mechanism of action of this exogenous PUFA. A naturally occurring substance, tung oil, contains a high concentration of αESA and shows some ability to control tumor growth *in vivo* [141]. In contrast to the roles of ACSL4 and ACSL1 in promoting ferroptosis, the related enzyme ACSL3 is more closely associated with inhibiting ferroptosis, especially in response to exposure of cells to exogenous MUFAs like oleate [111]. Exogenous oleate can be incorporated into membrane PLs, displace PUFAs, and reduce the accumulation of lipid hydroperoxides under ferroptosis-inducing conditions [142]. This ACSL3-dependent anti-ferroptotic mechanism may be active *in vivo* and contribute to metastasis: ACSL3 activity promotes the survival of metastatic melanoma cells that transit through the lymph fluid where they are exposed to a MUFA-rich environment [112]. An emerging picture is that an ACSL3/MUFA axis of lipid metabolism acts in opposition to an ACSL4/PUFA axis to balance the relative concentrations of MUFA-containing and PUFA-containing PLs to govern the overall oxidizability of the membrane and therefore the susceptibility to ferroptosis.

Once fatty acids are activated by ACSL enzymes, the resultant fatty acid-CoA species can be incorporated into more complex lipids, including PLs. Fatty acid-CoA species can be incorporated into PLs during *de novo* synthesis and during phospholipid remodeling (i.e., the Lands cycle) by lysophospholipid acyltransferases (LPLATs). Our knowledge of the structure and function of these enzymes, and the associated nomenclature of these species, is rapidly evolving [143]. In relation to ferroptosis, early evidence indicated that LPCAT3 (LPLAT12) may promote ferroptosis, presumably via incorporation of PUFA-CoAs into membrane PLs [133]. In lung cancer cells, LPCAT3 transcription may be controlled by the transcription factor ZEB1 together with yes-associated protein (YAP) [144], providing a possible rationale for the association between high *ZEB1* levels and higher cellular ferroptosis sensitivity [127]. Isolation of a specific LPCAT3 inhibitor has enabled more refined investigations which confirm that LPCAT3 can promote ferroptosis, but that loss of this enzyme alone does not completely inhibit this process [145]. This may be explained by the observation that inhibition of LPCAT3, while reducing the levels of C20:4-containing PLs, increases the levels of C22:4-containing PLs. This highlights the potential for redundancy in the LPLAT network, with multiple enzymes capable of contributing to PUFA-PL pools that, in turn, can promote ferroptosis in a context-dependent manner. Another emerging area is the study of reactions where PUFAs are directly transferred from one phospholipid species to another. Specifically, transmembrane protein (TMEM) 164 is proposed to enhance ferroptosis sensitivity by transferring C20:4 from phosphatidylcholine to ether PLs [146]. For unknown reasons, this reaction appears to enhance ferroptosis in some but not all cell types. The relative contributions of LPCAT3, TMEM164 and potentially other enzymes to shaping the oxidizable PUFA-PL pool requires further elucidation.

In the same way that ACSL4 and ACSL3 generally appear to have opposing roles in shaping the lipidome and ferroptotic sensitivity of cancer cells, LPCAT3 and the MBOAT1 and MBOAT2 enzymes may play opposing roles, with MBOAT1/2 being more specific for the insertion of MUFA-CoAs into membrane PLs, especially PEs [123]. The biochemical effect of MBOAT1/2 function is to increase the relative concentration of MUFA-containing PLs at the expense of PUFA-containing PLs, and thereby limit ferroptosis by reducing the overall oxidizability of relevant membranes in the cell. However, other MBOAT1/2-dependent changes in lipid metabolism within the cell cannot be ruled out. Intriguingly, *MBOAT1* and *MBOAT2* expression are regulated by the estrogen and the androgen receptors, respectively [123]. This suggests potential sex-specific differences in ferroptosis regulation in both normal and disease states. In the context of cancer therapy, where inhibition of MBOAT1/2 function may help unleash a pro-ferroptotic response, inhibitors of estrogen or androgen receptors may be combined with inducers of ferroptosis to achieve a more potent response. Moreover, one can envision MBOAT1 as a specific target for the development of therapies in estrogen receptor^+^ breast cancers and MBOAT2 as the relevant target in androgen receptor^+^ prostate cancer [123]. The development of small molecule inhibitors of MBOAT1 and MBOAT2, along with the upstream enzyme ACSL3, could allow for the development of potent new drug combinations targeting lipid metabolism to modulate ferroptosis for therapeutic benefit.

#### 2.2.2 Lipophilic vitamins

##### 2.2.2.1 Vitamin E

Vitamin E, a fat-soluble vitamin, acts as Nature's premier lipophilic RTA, safeguarding cells and tissues against excessive lipid peroxidation and ferroptosis. By scavenging lipid peroxyl radicals and halting the propagation phase through the formation of vitamin E radicals, vitamin E plays a crucial defense mechanism [147]. Dietary vitamin E comprises eight natural forms: α-, β-, γ-, and δ-tocopherol and α-, β-, γ-, and δ-tocotrienol, all of which effectively break the autoxidation chain reaction. Furthermore, vitamin E synergistically collaborates with other RTAs, such as CoQ_10_, by facilitating their regeneration [51].

Supplementation with vitamin E effectively prevents ferroptosis and associated disorders in cell culture conditions and animal models. The cell protective effect of vitamin E against cell death induced by cystine-deprivation (now known as ferroptosis) was reported in the 1980s [13], actually long before the term ferroptosis was coined. Vitamin E has also been shown to rescue certain tissues from the deleterious consequences induced by the tissue-specific disruption of *Gpx4* [148]. The first tissue where such a compensatory mechanism was demonstrated, was endothelial cells. While endothelial-specific *Gpx4* knockout mice do not display any overt pathologies under normal housing conditions, feeding these mice a vitamin E-deprived diet causes widespread endothelial cell death, multiorgan thrombus formation and early death of mice [149]. Such a compensatory mechanism is seemingly also at play in T cells [85], hepatocytes [150], myeloid cells [151], bone marrow-derived cells [91], hematopoietic stem cells using Vav- and Mx-Cre [152], and photoreceptor cells [85]. Notably, tissue vitamin E levels and dietary contents are a crucial factor when considering the role of ferroptosis in certain cell types or contexts. For example, endothelial cell- or hepatocyte-specific *Gpx4* knockout animals [52,149] do not show any overt phenotype when kept under a normal diet. However, subjecting these animals to a vitamin E-deficient diet results in tissue-specific cell death and subsequent mortality. This underscores the collaborative action of vitamin E and GPX4 in preventing ferroptosis *in vivo*. The physiological role of vitamin E as a potent natural inhibitor of ferroptosis is also highlighted by the fact that sustained vitamin E deficiency or mutations in the *TTPA* gene, encoding for α-tocopherol transfer protein, lead to neurodegeneration [153,154]. This neurologic phenotype is similar to Friedreich's ataxia, a genetic neurological disorder reportedly linked to ferroptosis [155], suggesting that endogenous vitamin E plays a preventive role against neuronal ferroptosis.

Mouse chow pellets commonly contain physiologically high levels of vitamin E, with concentrations varying among manufacturers (e.g., supplemented ∼50–150 mg/kg of vitamin E) and even within batches. Thus, when evaluating the ferroptotic phenotype in animal models, it is crucial to consider the variation in vitamin E levels within the experimental diet, depending on research objectives. In some instances, supplemented vitamin E in the normal chow can mask the ferroptotic phenotype as described above. This concern may also apply when using high-fat diets in animal studies, since lipophilic vitamins, including vitamin E, are abundant in the raw fat materials contained in the diet.

##### 2.2.2.2 Vitamin K

Vitamin K, another lipophilic vitamin, confers a strong anti-ferroptotic function, in addition to its well-known function linked to blood clotting. In the latter function, it serves as a cofactor for γ-glutamyl carboxylase, which catalyzes the carboxylation of vitamin K-dependent proteins, including coagulation factors [52,156]. Whereas the oxidized form of vitamin K (i.e., vitamin K quinone) lacks RTA activity, the fully reduced form of vitamin K (i.e., vitamin K hydroquinone) acts as a potent RTA and inhibitor of (phospho)lipid peroxidation. FSP1 efficiently reduces vitamin K quinone to its corresponding vitamin K hydroquinone, by consuming NAD(P)H in a manner similar to FSP1-mediated CoQ_10_ reduction [52]. The reaction of vitamin K hydroquinone with lipidperoxyl radicals generates vitamin K quinone, which can be reduced by FSP1 again using two electrons sourced from NAD(P)H. This FSP1-dependent non-canonical vitamin K cycle, termed the Mishima cycle [156], functions to protect cells against detrimental lipid peroxidation and ferroptosis. In addition, FSP1 is the enzyme mediating warfarin-resistant vitamin K reduction in the canonical vitamin K cycle, crucial for blood coagulation. This FSP1-mediated reduction of vitamin K in the canonical cycle is responsible for the antidotal effect of vitamin K against poisoning with warfarin, which is an inhibitor of vitamin K epoxide reductase and a commonly used anticoagulant drug [52,92].

Among the various forms of vitamin K, phylloquinone (known as vitamin K1) is sourced from dietary leafy green vegetables, and can be converted to menaquinone-4 (a form of vitamin K2) in the body. Menadione (known as vitamin K3) is a synthetic hydrophilic variant and an intermediate in the biosynthesis of menaquinone-4. While these three forms of vitamin K exhibit anti-ferroptotic properties, menaquinone-4 shows the most potent antiferroptotic effect and RTA activity in its reduced form *in vitro* [52]. Additionally, administration of a pharmacological dose of vitamin K (menaquinone-4 or phylloquinone) mitigated tissue damage associated with ferroptosis induced by ischemia-reperfusion injury and tissue-specific conditional *Gpx4* knockout [52,157]. Menadione can also prevent ferroptosis, but at high dose (>10 μM) where it shows cellular toxicity due to the generation of superoxide [[158], [159], [160]].

##### 2.2.2.3 Vitamin a and others

Among other lipophilic vitamins, vitamin A, such as retinol and retinoic acid, has also been reported to inhibit ferroptosis through its apparently modest RTA activity and/or the modulation of transcriptional regulation [161,162]. An enzyme involved in vitamin A metabolism, retinol saturase has been reported as a regulator of cellular ferroptosis sensitivity by converting anti-ferroptotic retinol into 13, 14-dihydroretinol, which exhibits decreased RTA activity. In contrast to other lipophilic vitamins, vitamin D does not prevent ferroptosis - at least in cell culture condition [52] - although 7-dehydrocholestrol, a precursor of vitamin D, has been shown to prevent ferroptosis by sparing PLs from autoxidation [[163], [164], [165]].

#### 2.2.3 Relevance of iron in ferroptosis

**Fig. 5:**
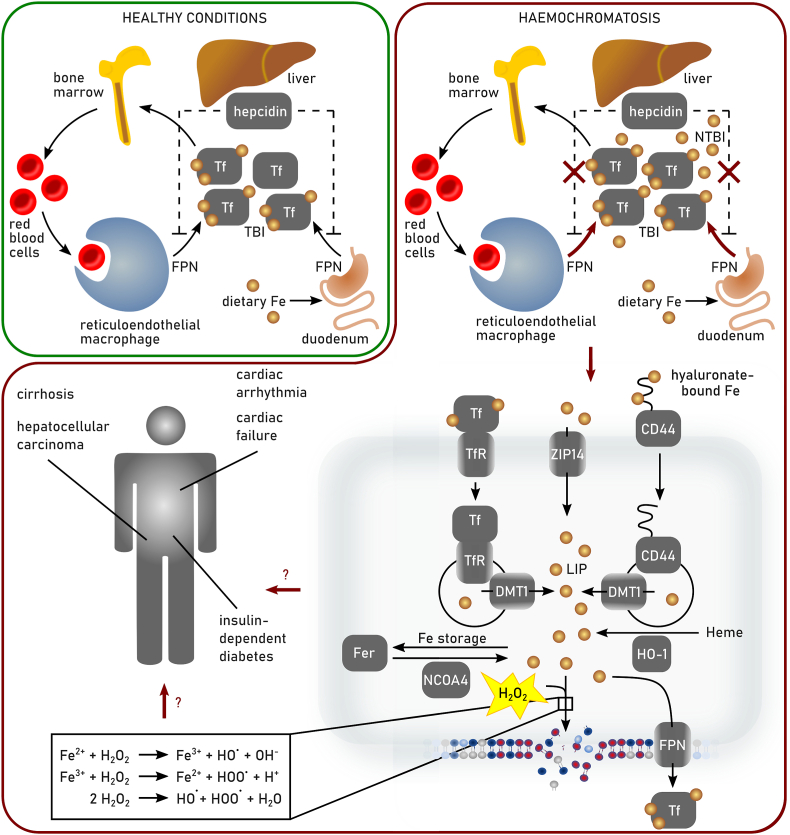
**Iron metabolism and ferroptosis.** Iron metabolism is regulated at the systemic and the cellular level. Iron efflux from duodenal enterocytes and macrophages is controlled by hepcidin, a peptide hormone produced by the liver which binds to and induces degradation of the iron exporter ferroportin. In iron overload diseases (e.g. haemochromatosis), the hepcidin/ferroportin axis is disturbed and iron is released in excess into the bloodstream. The capacity of transferrin to bind iron is exceeded and a potentially toxic form of iron appears, called non-transferrin bound iron (NTBI). TBI (transferrin-bound iron) is imported into cells in a controlled manner *via* the transferrin receptor Tfr, whereas NTBI is taken up in an uncontrolled manner *via* NTBI importers such as ZIP14 and CD44. Iron uptake enlarges the labile iron pool (LIP). Through the generation of hydroxyl (HO^•^) or hydroperoxyl radicals (HOO^•^) by the Fenton reaction, free and redox-active iron can trigger lipid peroxidation and ferroptosis. Iron storage in ferritin (Fer) or iron export *via* ferroportin reduce the LIP and therefore protect cells against ferroptosis. The release of free iron from Fer by NCOA4 mediated ferritinophagy or from heme by heme oxygenase (HO-1) increases the LIP and potentially sensitizes the cells to ferroptosis. A growing number of studies suggests that ferroptosis plays a critical role in the iron-mediated hepatic (cirrhosis, hepatocellular carcinoma), pancreatic (insulin-dependent diabetes) or cardiac (cardiac arrhythmia, cardiac failure) complications observed in iron overload disorders.

The name ferroptosis is derived from the Latin word *ferrum*, which translates as iron. The importance of iron in ferroptotic cell death became clear in 2008, as the iron chelator deferoxamine could block erastin and RSL3-induced cell death [26]. In the same year, a report showed that GPX4 could sense 12/15-lipoxygenase-derived lipid peroxidation, putting forward peroxidized lipids as a hallmark of an at the time unnamed cell death modality [24]. Lipid peroxidation can be triggered by hydroxyl or hydroperoxyl radicals. These radicals are generated by the Fenton reaction, a redox reaction involving Fe^3+^ and Fe^2+^, which are susceptible to convert hydrogen peroxide into oxygen-centered radicals [166] (Fig. 5). Moreover, Fe^2+^ reacts directly with lipid hydroperoxides to produce lipid alkoxyl radicals in a Fenton-like reaction. Work on the natural product salinomycin, which had been identified in 2009 to be able to selectively target the so-called cancer stem cell niche in solid tumors [167], showed that the molecule exerts its activity by sequestering iron in lysosomes [168], using fluorescent labeling of the potent salinomycin derivative ironomycin and an Fe^2+^-specific lysosomal probe. The relatively weak binding of ironomycin to iron still permits the Fenton reaction to occur in this organelle, which can lead to lipid peroxidation and ferroptotic cell death. This study put lysosomal iron into the limelight as an important pool of the metal in ferroptosis. Importantly, ironomycin-induced cell death could be inhibited by ferrostatin, the iron chelator deferoxamine and a reducing agent, giving a powerful hint that indeed ferroptotic cell death is induced by the natural product and its derivatives. This work also advocates lysosomal iron as a druggable target that can be exploited, potentially as a therapeutic approach to either promote or inhibit ferroptosis based on the clinical context. A subsequent study demonstrated that an iron-cleavable prodrug of ironomycin, which accumulates in lysosomes, exhibits ferroptotic activity in organoid models of pancreatic ductal adenocarcinoma [169]. This prodrug consisting of ironomycin linked to dihydroartemisinin can fragment in the presence of Fe^2+^ to afford radicals leading to lipid peroxidation. Since this chimera also accumulates in lysosomes, the drug can be released upon reaction with abundant lysosomal Fe^2+^. The importance of iron, in particular lysosomal iron, during ferroptosis was substantiated by the finding that an iron endocytosis protein, transferrin receptor 1 (TfR1), was proposed as a ferroptosis marker [170]. Previous work in persister cancer cells implicated a role of iron in heightening their vulnerability to ferroptosis [171]. This led to the discovery of a previously unchartered iron endocytosis pathway that utilizes hyaluronic acid and its main cell surface receptor CD44 for iron uptake [172]. Since *TfR1* mRNA is destabilized in the presence of iron due to a lack of iron regulatory protein (IRP) binding to its iron responsive elements in the untranslated region of its mRNA, cancer cells that require high amounts of iron to drive cell state transitions exploit a distinct mechanism. Under these conditions, CD44, which is positively regulated by iron at the epigenetic level, takes over as the main iron uptake protein. CD44 might therefore be considered as a predictor of ferroptosis vulnerability. Indeed, CD44 is an important importer of metals, including iron and copper [172,173], which directly catalyze specific biochemical reactions required for the regulation of cell plasticity, i.e. changing cell state, as seen in the genesis of persister cancer cells or activation of immune cells, independently of genetic alterations. The redox-active Fe^2+^ form exists transiently as a labile pool, prior to being translocated from the endo-lysosomal compartment by divalent metal transporter 1 (DMT1), making it a potentially critical driver of ferroptosis in this organelle. Intracellular iron traffics preferentially to the nucleus, where it is used as the catalyst of iron-dependent demethylases to promote epigenetic changes enabling expression of specific genes, underlying cell plasticity and acquisition of specific cell functions [172,173]. The increased trafficking of iron via lysosomes and the resulting increased pools of labile iron could make these cells more vulnerable to ferroptosis. Further work is required to pinpoint the exact contributions of different cellular iron pools to ferroptosis, including those in the mitochondria, endoplasmic reticulum and lysosomes. An interesting hypothesis would be that lipid perxidation is first initiated in or at the periphery of lysosomes, where the labile and redox-active pool of iron is transiently present, to propagate to other organelles such as the endoplasmic reticulum, where there is abundant lipid peroxidation [174].

##### 2.2.3.1 How does cellular iron handling contribute to ferroptosis?

The ubiquitously expressed TfR1 takes up transferrin-bound iron into cells. Iron is required for maintaining biological processes, such as respiration, DNA synthesis, and oxygen transport. However, when iron is unbound and/or in excess it is toxic. Therefore, sufficient cellular iron supplies and appropriate iron handling inside cells must be assured by the coordination of iron uptake, storage, utilization and export [175,176]. A dysregulation of these critical processes perturbs cellular iron homeostasis and may trigger ferroptosis via the Fenton reaction provoked by the formation of hydroxyl and hydroperoxyl radicals [177]. Transferrin-bound Fe^3+^ binds to TfR1 and induces clathrin-dependent endocytosis (Fig. 5). Endosomes are acidified and iron is reduced from Fe^3+^ to Fe^2+^ by specific reductases (e.g., proteins of the six-transmembrane epithelial antigen of the prostate family) prior to being translocated to the cytosol via specific metal transport proteins, such as DMT1/Solute carrier family 11 member 2 (SLC11A2) [178]. Blocking this transporter with specific inhibitors causes accumulation of reactive iron in lysosomes and promotion of ferroptosis [179]. In the cytosol, poly rC binding proteins (PCBPs) act as iron chaperones, which deliver ferrous iron to the iron storage protein ferritin. When PCBPs are dysfunctional, the labile iron pool (LIP) is significantly increased [180], which can sensitize cells to ferroptosis. Alternatively, non-transferrin-bound iron (NTBI), a ferrous iron conjugate can be taken up by cells via the metal transporter SLC39A14 (ZIP14) [181]. Increased iron uptake through iron transporters such as TfR1 and SLC39A14 dictates cell ferroptosis [136,182]. Transferrin and ferritin can be considered as anti-ferroptosis proteins as they bind and detoxify ionic iron [182,183]. However, in the setting of nutrient depletion, transferrin has been shown to be essential for regulating cellular ferroptosis [184]. Similar to cytosolic ferritin, mitochondrial ferritin can also inhibit the process of ferroptosis through modulating the cellular LIP [185]. In mammalian cells, the sole known iron exporter ferroportin 1 (FPN1), is mainly regulated by hepcidin, an iron-regulatory peptide hormone produced in the liver (Fig. 5). Recently, ring finger protein 217 was identified as an E3 ubiquitin ligase that controls hepcidin-mediated FPN1 degradation [186]. Decreased FPN1 expression increases the cellular sensitivity to ferroptosis due to the intracellular accumulation of iron [187].

Ferroptosis sensitivity is further controlled by heme oxygenase 1 (HO-1), an enzyme that degrades heme into biliverdin, carbon monoxide and ferrous iron [188]. Most recently, metformin, the first-line treatment for type 2 diabetes, was reported to exacerbate acute kidney injury by ferroptosis provoked NETosis in an iron-dependent manner through the binding of CD184 receptor to the Ngal-metformin-Fe complex [189]. Furthermore, in conditions of iron depletion, nuclear receptor coactivator 4 (NCOA4) targets ferritin to the lysosome (ferritinophagy) for degradation. This process releases iron from ferritin and facilitates ferroptosis [185]. Interestingly, in response to pro-ferroptotic stimuli, prominin-2 protects cells against ferroptosis via exosome-mediated cellular export of ferritin [190]. As a key pathogenic mechanism, interconnected iron metabolism and ferroptosis have been well-recognized as a valid target for treating many diseases, including cancer, neurodegeneration, cardiovascular disease, liver disease, etc. [177,181,191].

Mitochondrial iron homeostasis is another regulatory layer that modulates ferroptotic sensitivity. For example, mitoferrins 1/2 (SLC25A37/28) are localized in the inner mitochondrial membrane, where they control mitochondrial iron availability for heme and iron-sulfur cluster biogenesis. In contrast to the mitoferrins [192,193], the Fe_2_S_2_ coordinating proteins CDGSH iron sulfur domain (CISD) 1 (mitoNEET) and CISD2 (NAF1) reduce ferroptosis sensitivity by limiting mitochondrial iron uptake [177]. Cellular iron metabolism is orchestrated by the IRP and iron responsive element regulatory axis that post-transcriptionally controls iron related genes, such as ferritin, TfR1, FPN1 and DMT1. Accordingly, both IRP1 and IRP2 play import roles in regulating ferroptosis [194]. Iron metabolism genes are also controlled at the transcriptional levels via NRF2, which has been shown to control iron homeostasis and ferroptosis through controlling the expression of the HECT and RLD domain containing E3 ubiquitin ligase 2, that regulates NCOA4, F-box and leucine rich repeat protein 5 and vesicle-associated membrane protein 8 [195].

##### 2.2.3.2 Systemic iron homeostasis, iron overload diseases and ferroptosis

In addition to being regulated at the cellular level, iron homeostasis is controlled at the systemic level by the hepcidin/FPN1 regulatory axis. Hepcidin is a small peptide hormone produced by the liver that inhibits cellular iron efflux by binding to the iron exporter FPN1, inducing its internalization and degradation. As a consequence, intestinal iron absorption and macrophage iron release is inhibited [196]. The hepcidin/FPN1 axis is disrupted in various hereditary and acquired pathologies that lead to anemia or iron overload. For example, in the frequent hereditary iron overload disorder hemochromatosis (carrier frequency of 1 in 8) mutations in upstream regulators (human homeostatic iron regulator protein, TfR2, hemojuvelin) or the hepcidin receptor ferroportin impair the hepcidin/ferroportin regulatory system. As a consequence, excess iron is released into the bloodstream from dietary uptake via duodenal enterocytes and from iron-recycling macrophages exceeding the capacity of transferrin to bind iron. This results in the appearance of a potentially toxic form of free iron called NTBI that accumulates mainly in parenchymal cells such as hepatocytes, pancreatic cells and cardiomyocytes. Through the production of hydroxyl or hydroperoxyl radicals via the Fenton reaction or alkoxyl radicals via Fenton-type reactions, cellular iron accumulation can lead to severe complications in the liver (cirrhosis, hepatocellular carcinoma), the heart (hypertrophy, insufficiency, cardiac arrhythmia) and the endocrine glands (insulin-dependent diabetes) [175,197] (Fig. 5).

A growing number of studies suggests that iron overloaded tissues are damaged by ferroptosis. Indeed, ferroptosis seems to be induced in the severely iron-overloaded liver of mice with juvenile subtypes of HC (hemojuvelin encoding *Hjv*^*−/−*^ and Smad4Alb/Alb) [198]. This is also the case in the pancreas, where repeated injections of iron dextran cause ferroptosis of acinar cells [199]. This observation is in agreement with previous studies showing that extensive tissue damage in the iron-overloaded pancreas was associated with lipid peroxidation (*Hepcidin* knockout and FpnC326S mice) [200,201]. It is important, however, to note that iron accumulation does not always induce ferroptosis in all organs. This was illustrated by the analysis of FpnC326S mice that accumulate a comparable concentration of iron in the liver and pancreas, but present tissue damage only in the pancreas causing their early death by exocrine pancreatic failure [201]. This organ-dependent response clearly demonstrates that in a situation of tissue iron accumulation, the induction of ferroptosis depends on various factors. In this context it is important to realize that the mechanisms of iron handling differ from one cell type to another. For example, the severity of tissue iron overload can be dictated by the expression level of NTBI importers, in particular SLC39A14 (ZIP14). *Slc39a14* ablation has been shown to prevent iron deposition in hepatocytes and pancreatic acinar cells of hemochromatosis mice [202]. Likewise, the iron export capacity of cells and tissues may affect ferroptosis sensitivity. Elemental iron is exported from cells via FPN1, the target receptor of hepcidin. However, iron also leaves cells via ferritin. Since iron-loaded ferritin lacks a canonical signal sequence, it can be exported by secretory autophagy or endosomal microautophagy. Ferritin secretion in exosomes is a dynamic process and can be activated in response to ferroptosis, iron loading or high lipogenic activity. The fate of ferritin-containing exosomes is not well understood, but they are probably captured by neighbouring cells. Furthermore, iron may also be exported from the cell as heme iron. In the absence of these iron export pathways iron will accumulate in cells and contribute to ferroptosis.

Beyond the entry and exit of iron, the management of the metal within the cells may also contribute to ferroptosis sensitivity as discussed in the previous section. For example, in the context of iron overload, a cell type that has a lower iron storage capacity due to weak ferritin expression, will have a higher risk of ferroptosis as a result of a larger LIP [183]. Whether or not a cell undergoes ferroptosis further depends on the ability of its antioxidant defense system to prevent oxidative damage induced by iron overload. Here, again, not all organs are equal [203]. Despite this, the organ selectivity of ferroptosis-mediated tissue damage induced by iron overload remains poorly understood. However, the ongoing identification of the metabolic pathways regulating this cell death process is progressively improving our understanding of why some organs are more sensitive than others. Deciphering the molecular contribution of iron in ferroptosis is of great interest to patients, as its prevention appears to be a promising approach to prevent/reduce tissue damage in iron overload disorders.

#### 2.2.4 GTP-cyclohydrolase 1/tetrahydrobiopterin/dihydrofolate reductase axis

##### 2.2.4.1 Canonical role of tetrahydrobiopterin in cellular redox balance

BH4 is a GTP-derived pteridine best known for its cofactor function in the synthesis of dopamine and serotonin precursors from aromatic amino acids. In addition to using BH4, phenylalanine, tyrosine, and tryptophan hydoxylases require oxygen and iron. In these reactions, BH4 is oxidized to stabilize the oxo-iron complex that allows for the hydroxylation of the substrate [204]. BH4 is also a cofactor for the three isoforms of nitric oxide synthase (NOS) [205], where it acts as a single electron donor, and alkylglycerol monoxygenase [206]. Finally, given that BH4 is readily oxidized into dihydrobiopterin (BH2), it has roles as an endogenous antioxidant [207,208]. Importantly, BH4 depletion and disruption of the BH4/BH2 ratio can lead to NOS-uncoupling and the formation of superoxide and peroxynitrite.

**Fig. 6:**
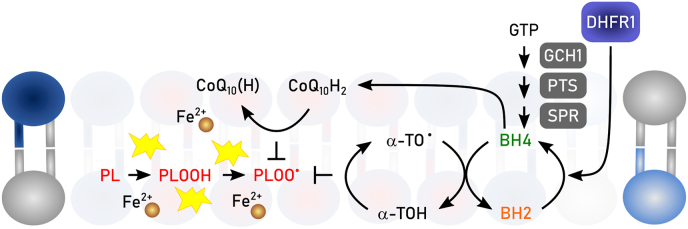
**Ferroptosis protection by the GCH1/BH4/DHFR axis**. Tetrahydrobiopterin (BH4) is synthesized through a series of steps catalyzed by GTP cyclohydrolase 1 (GCH1, the rate-limiting enzyme), 6-pyruvoyl-tetrahydropterin synthase (PTS), and sepiapterin reductase (SPR). BH4 acts as a radical trapping antioxidant in lipid membranes, directly quenching lipid radicals. BH4 can regenerate oxidized a-tocopherol (vitamin E), an endogenous lipophilic antioxidant. Oxidized BH4 (BH2) is recycled back to BH4 by DHFR1. Additionally, BH4 may contribute the synthesis of CoQ_10_, promoting the protective effects of the NAD(P)H/FSP1/CoQ_10_ system.

BH4 availability is largely driven by the Mg^2+^, Zn^2+^, and NADPH dependent *de novo* synthesis pathway composed of the rate limiting enzyme GTP cyclohydrolase I (GCH1) together with 6-pyruvoyl-tetrahydropterin synthase and sepiapterin reductase. GCH1 expression is controlled at the transcriptional and post-translational level and represents the rate-limiting step of BH4 biosynthesis. Interestingly, cytokines including interferon (IFN) γ [209], TNFα, and interleukin (IL) 1β [210] can induce GCH1 expression in some immune cells and other cell types. High levels of BH4 promote the binding and inactivation of GCH1 via GCH1 feedback regulator (GFRP), thereby regulating its activity on a post-translational level. Conversely, high levels of phenylalanine can convert the inactive GCH1-GFRP complex back to its active form, promoting BH4 synthesis [211]. In addition to the *de novo* synthesis pathway, BH4 can be recycled from the inactive forms generated during enzymatic reactions or from quenching ROS. Specifically, pterin-4a-carbinolamine dehydratase and dihydropteridine dehydratase [212] can regenerate BH4 from the product of the hydroxylase reactions, while DHFR [213] regenerates BH4 from its oxidized form, BH2 (Fig. 6).

##### 2.2.4.2 BH4 as a ferroptosis suppressor

Recently, both loss [53] and gain of function [54] CRISPR screens revealed BH4 as a potent ferroptosis suppressor. Indeed, loss of GCH1, 6-pyruvoyl-tetrahydropterin synthase or sepiapterin reductase sensitize cancer cells to GPX4 inhibition [53] and overexpression of GCH1 is sufficient to increase resistance to *Gpx4* loss and inhibition and imidazole ketone erastin (IKE) treatment [54]. GCH1 expression across cell lines predicts dependence on BH4 during ferroptosis and supplementation with BH4 or BH2 improves ferroptosis resistance in these cell lines. Importantly, genetic deletion or pharmacological inhibition of DHFR with the chemotherapeutic methotrexate sensitizes cells to GPX4 inhibition and prevents BH2-mediated rescue [53]. Thus, like the FSP1/ubiquinone/vitamin K axis the DHFR/BH4 system emerges as a GPX4-independent mechanism to suppress ferroptosis.

Molecular characterization of the protective properties of BH4 showed that the pteridine protects PUFA-esterified PLs from degradation during ferroptosis and increases the levels of reduced CoQ_10_ [53,54] (Fig. 6). Liposomal co-autoxidation reactions with phosphatidylcholine and the lipid peroxidation reporter STY-BODIPY (FENIX system) revealed that BH4 is an effective direct inhibitor of lipid peroxidation. Indeed, BH4 inhibits autoxidation similarly to α-tocopherol, one of the most effective endogenous lipophilic RTAs. Though BH2 alone did not inhibit lipid peroxidation, addition of recombinant DHFR, but not quinoid dihydropteridine reductase, dramatically improved its RTA activity and this was fully reversed by the addition of methotrexate [53]. As such, the α-tocopherol/BH2/DHFR system is reminiscent of the α-tocopherol/CoQ_10_/FSP1 system [50,51]. Notably, BH4 and α-tocopherol, but not BH4 and CoQ_10_, synergized to significantly inhibit lipid peroxidation in liposomes. Thus, the increased levels of reduced CoQ_10_ observed in GCH1-overexpressing cells activity might be explained by an increase in the conversion of phenylalanine to tyrosine by phenylalanine hydroxylase [214], which can be used in CoQ_10_ production [54].

##### 2.2.4.3 BH4 in pathophysiology and cancer

In humans, BH4 deficiency resulting from mutations in the *de novo* synthesis or recycling pathways primarily manifests as a deficit in neurotransmitters and progressive neurological symptoms. GCH1 and BH4 are required for murine embryonic development [215], however, conditional deletion of GCH1 in animal models has provided insights into the role of BH4 in distinct cell types. Indeed, BH4 has emerged as a critical regulator of redox homeostasis in macrophages, where loss of GCH1 promotes the accumulation of iNOS-dependent and -independent ROS and impairs NRF2 activation [216]. Human and murine T cells deficient in GCH1 and BH4 display defects in activation and proliferation. Strikingly, BH4 depletion in T cells ameliorates autoimmunity and allergic inflammation while GCH1 overexpression enhances anti-tumor immunity in mouse models. BH4 depletion in T cells reduces iron levels and upregulates levels of the iron storage proteins, ferritin and mitoferrin [217]. Additionally, overexpression of GCH1 or BH4 supplementation in rat skin and skin cells prevented radiotherapy-induced oxidative damage resulting from BH4 oxidation and, consequently, NOS uncoupling. Further investigations in skin cells uncovered that GCH1 is directly regulated by NRF2 [218]. These findings raise the possibility that targeting BH4 synthesis in cancer cells can synergize with radiotherapy. Though these earlier studies clearly indicate an antioxidant role for BH4, its anti-ferroptotic role in disease remains to be tested.

The first reports of BH4 as a ferroptosis suppressor indicate that cancer cell lines with high levels of GCH1 expression could be more dependent on BH4 for ferroptosis protection [53,54]. Indeed, GCH1 expression predicted ferroptosis sensitivity in some cell lines [54]. Interestingly and consistent with the major role of BH4 in leukocyte biology, blood cancer cell lines seemed to be overrepresented among the most sensitive to ferroptosis and most dependent on BH4 synthesis, compared to the other cell line origins tested. This suggests that targeting BH4 may improve the efficacy of current therapies for blood cancer that induce ferroptosis, such as immunotherapy [219] and radiation [220]. Moreover, a recent study suggests that GCH1 and BH4 depletion in colorectal cancer cells increases ferrous iron accumulation and ferroptosis susceptibility [221]. Given the potential of ferroptosis induction as an anti-cancer strategy, understanding the role of BH4 in tumorigenesis and therapy resistance is crucial.

#### 2.2.5 Cysteine and glutathione

##### 2.2.5.1 Mechanisms of cysteine acquisition

**Fig. 7:**
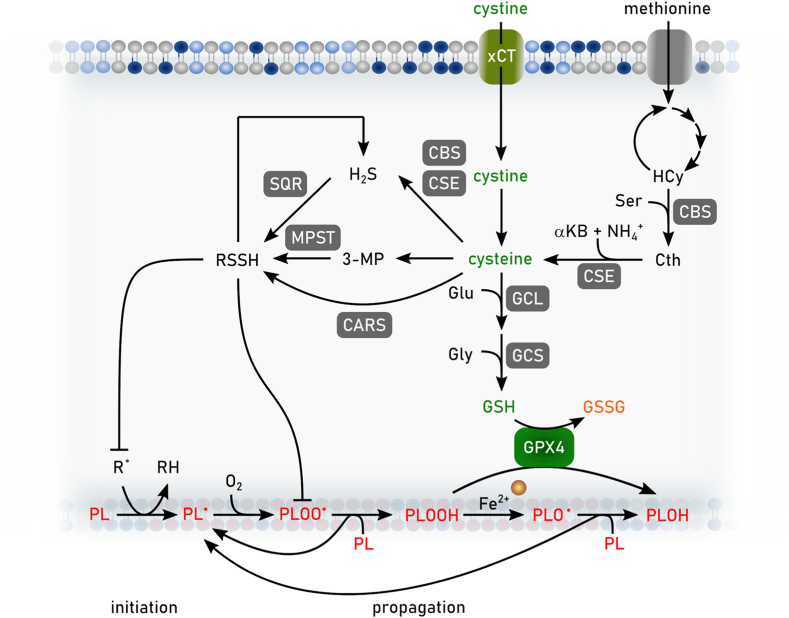
**Cysteine metabolism and ferroptosis.** Schematic overview of the main pathways involved in cysteine uptake, *de novo* cysteine synthesis, and cysteine utilization that regulate ferroptosis sensitivity in cells. Cysteine can be taken up in its oxidized form cystine via the xCT transporter, or synthesized *de novo* from methionine and serine via the transulfuration enzymes cystathionine β-synthase (CBS) and cystathionine γ-lyase (CSE). Production of glutathione (GSH) from cysteine supports the activity of glutathione peroxidase 4 (GPX4) to detoxify phospholipid hydroperoxides (PLOOH) and mitigate the propogation of the lipid peroxidation cycle. Moreover, cysteine-derived sulfane sulfur species (hydropersulfides, RSSH) act as radical trapping agents that can suppress the initiation and propagation of the radical chain reaction driving lipid peroxidation.

Cysteine is a thiol (-SH) containing amino acid that plays a central role in protection against ferroptosis. In the oxidizing conditions found in tissue cultures and the circulatory system to some extent, cystine, the oxidized form of cysteine, is the primary extracellular form of cysteine. However, it is known that there are huge discrepancies in cysteine availability between tissue culture conditions and a whole organism [222]. Whereas in tissue culture conditions almost all cysteine is oxidized to cystine within minutes, in transgenic mice lacking the substrate-specific subunit of the cystine-glutamate amino acid transporter xCT (aka SLC7A11), there are substantial amounts of cysteine present in plasma [222]. Nonetheless, in cell culture cells predominantly import cystine through the system xCT antiporter in exchange for glutamate [223]. xCT was identified as the target of erastin, a potent inducer of ferroptosis in cultured cancer cells [5]. *In vivo* studies using a genetically engineered mouse model of pancreatic ductal adenocarcinoma have demonstrated that genetic disruption of SLC7A11 or enzymatic depletion of circulating cyst(e)ine also induces ferroptosis [224]. Additionally, in some tissues, cysteine can be synthesized *de novo* via the transsulfuration pathway, involving the enzymes cystathionine β-synthase (CBS) and cystathionine γ-lyase (CSE) (Fig. 7). In the first step of the transsulfuration pathway, CBS catalyzes the condensation of homocysteine derived from the methionine cycle with serine to yield cystathionine, which is subsequently cleaved by CSE to release cysteine while generating α-ketobutyrate as a byproduct. However, while cyst(e)ine significantly contributes to the cysteine pool in diverse tissues and tumors *in vivo*, only in liver and hepatocellular carcinoma there is evidence for a significant contribution of the transsulfuration pathway as a source of cysteine [225]. Therefore, targeting cyst(e)ine transport and/or extracellular availability is expected to impact its availability in most contexts. Yet, more work is needed to understand the dependency of different tissues and tumors on specific cysteine/cystine transport systems.

##### 2.2.5.2 Cysteine and glutathione

One of the primary mechanisms by which cysteine protects against ferroptosis is through its role in the biosynthesis of GSH, the most abundant intracellular antioxidant. The synthesis of GSH involves two key enzymes: γ-glutamyl cysteine ligase (γ-GCL) and GSH synthase (GSS). γ-GCL catalyzes the ligation of cysteine with glutamate, followed by the addition of glycine by GSS, resulting in the formation of the γ-glutamyl-cysteinyl-glycine tripeptide (Fig. 7). While cysteine availability is typically the limiting factor in this process, it is not always the case. When cysteine becomes scarce, GSH synthesis is impaired, leading to a depletion of GSH levels. This depletion is not solely caused by impaired synthesis; cysteine starvation also triggers the expression of ChaC GSH specific gamma-glutamylcyclotransferase 1 (CHAC1), an enzyme responsible for hydrolyzing GSH [29]. The induction of CHAC1 is a result of the activation of the activating transcription factor 4 (ATF4)-mediated amino acid starvation response. It has been recently shown that in melanoma cell lines ATF4 is also able to augment NRF2 through a combination of transcriptional and metabolic mechanisms contributing to adaptation [226]. Thus, cysteine starvation is likely to induce more rapid depletion of GSH than inhibition of GSH synthesis. GSH mediates protection from ferroptosis through multiple mechanisms. Most importantly, GSH supports the GSH/GPX4 axis (see chapter 2.1.1), which is the main system that counteracts ferroptosis by limiting unrestrained lipid peroxidation. GSH is also used by other GSH peroxidases to reduce hydrogen peroxide (H_2_O_2_) and other organic peroxides, thereby limiting H_2_O_2_ availability for reactivity toward iron to generate hydroxyl radicals that can initiate the lipid peroxidation process. GSH has other roles within the cell, including metal chelation, drug export, methylglyoxal detoxification, and nucleotide metabolism, which may contribute with these redox functions to influence ferroptosis sensitivity. Indeed, suppression of nucleotide metabolism was shown to protect against ferroptosis by sparing GSH for scavenging peroxides via professional redox enzymes [227]. Additional work is needed to understand the interplay between other GSH-dependent reactions during ferroptosis.

##### 2.2.5.3 Beyond GSH – the role of cysteine metabolites

Cysteine's role in cellular homeostasis extends beyond its involvement in GSH synthesis. Firstly, cysteine is essential for the synthesis of iron-sulfur clusters, which serve as electron carriers and catalytic centers in enzymes and proteins involved in energy metabolism, DNA repair, and other vital processes. Cysteine acts as the sulfur donor during cluster assembly, starting with the formation of a persulfide (-SSH) intermediate on a protein scaffold, followed by the incorporation of iron to form a Fe_2_S_2_ cluster. Disruption of this process can activate the iron starvation response and can lead to iron accumulation, triggering ferroptosis [228]. Secondly, cysteine is critical for CoA synthesis, an essential cofactor in energy production, fatty acid synthesis, and amino acid metabolism. Cysteine, along with pantothenate (Vitamin B5), serves as a precursor for CoA synthesis. Although studies have shown that exogenous CoA can rescue ferroptosis [229] and inhibiting CoA synthesis can promote ferroptosis [224], the exact mechanism by which CoA influences ferroptosis remains unclear. Thirdly, cysteine can generate free persulfides and polysulfide (-S(n)SH) species, which can protect against ferroptosis by scavenging free radicals and inhibiting lipid peroxidation independently of GPX4 [230,231] (see chapter 2.2.6). Finally, cysteine deficiency leads to glutamate accumulation due to impaired export by the xCT transporter and reduced utilization for GSH synthesis. Interestingly, the GSH synthesis machinery has an additional function independent of GSH synthesis under low cysteine conditions, in which γ-GCL ligates glutamate to other amino acids to scavenge glutamate into γ-glutamyl peptides and prevent glutamate toxicity [232]. In sum, cysteine metabolism plays a central role in ferroptosis, controlling cellular redox balance, antioxidant defenses, and susceptibility to lipid peroxidation. The intricate relationship among cysteine uptake, synthesis, and utilization for GSH production ultimately determines the cell's response to ferroptotic stimuli. However, further investigations are needed to fully comprehend the dependency on specific mechanisms of cysteine acquisition particularly in tissues and tumors, providing valuable insights into the pathophysiology of oxidative stress-related diseases and potential therapeutic targets. Additionally, more research is required to elucidate the involvement of cysteine-derived molecules in ferroptosis sensitivity and the compartmentalization of cysteine under limiting conditions to support cellular processes. Advancements in this field hold promise for the development of novel strategies to modulate ferroptosis and potentially mitigate the impact of oxidative damage in various pathological conditions.

#### 2.2.6 Sulfane sulfur species and ferroptosis

Around 20 years ago, hydrogen sulfide (H_2_S) was recognized as a ‘gasotransmitter’, along with nitric oxide and carbon monoxide. The investigation of its biological roles led to the realization that at least some of its effects are caused by sulfur species derived from H_2_S, so-called sulfane sulfur (S^0^) species. Subsequently, it became clear that S^0^ species are produced by all living organisms [233]. Hydropersulfides (RSSH) emerged as one of the most prominent S^0^ species. In essence, hydropersulfides are thiols with an extra sulfur atom. In mammalian cells, hydropersulfides can reach intracellular concentrations of up to 100 μM [234]. They can be synthesized by several metabolic pathways, all of which use cysteine (or homocysteine) as the sulfur source. Some, but not all of these pathways involve H_2_S as an intermediary metabolite [235]. Intracellular RSSH levels were observed to increase under oxidative stress [230,236]. Moreover, N-acetyl cysteine, a pro-drug of cysteine, was found to protect cells against oxidative stress by promoting increased RSSH synthesis [237]. These and other observations implicated RSSH as cytoprotective agents.

##### 2.2.6.1 What makes hydropersulfides special?

Several charcteristics of RSSH have been proposed to explain their antioxidative and cytoprotective properties. RSSH are more nucleophilic than thiols (RSH). They react with electrophiles (e.g., H_2_O_2_) 10–100 times faster than thiols [238]. However, their relatively high reactivity as nucleophiles is unlikely to explain their antioxidative properties inside cells, since RSSH are at least 100 times less abundant than RSH [234]. Arguably, however, the most striking difference between the RSSH and RSH is their reactivity towards free radicals. RSSH are generally much better H atom donors than RSH, due to the greater stability of the resulting perthiyl radicals (RSS^•^), relative to thiyl radicals (RS^•^) [239]. Importantly, RSS^•^ are not observed to abstract H-atoms from other molecules. They also do not seem to react with molecular oxygen. Instead, they rapidly dimerize to yield a non-radical product, a tetrasulfide (RSSSSR) [240,241]. Therefore, RSSH act as a ‘radical sink’, eliminating free radicals through combination [230]. Importantly, tetrasulfides can be reduced back to RSSH, further sustaining the effect of RSSH in radical scavenging [230].

##### 2.2.6.2 Hydropersulfides scavenge free radicals inside living cells

Recently, the ability of persulfides to scavenge intracellular radicals was investigated using a chemogenetic approach. Briefly, expression of the heme peroxidase apurinic/apyrimidinic endodeoxyribonuclease 2 in the cytosol enabled the controlled generation of free radicals. Their intracellular concentrations were followed in real-time by luminescence. The exogenous addition of persulfide donors lowered the radical concentration in a dose-dependent manner. Moreover, the manipulation of endogenous persulfide levels by overexpressing or silencing genes involved in persulfide generation or degradation, including those encoding CSE and persulfide dioxygenase, revealed a correlation between endogenous persulfide levels and intracellular radical scavenging capacity [230].

##### 2.2.6.3 Hydropersulfides inhibit lipid peroxidation and ferroptosis

RTAs inhibit the propagation of lipid peroxidation by intercepting lipid peroxyl radicals (Fig. 7). A series of studies has now reveale innocuous perthiyl radicals. Indeed, synthetic RSSH [231] and inorganic polysulfides (HSS_n_SH) [242] were shown to act as RTAs in liposomal phospholipid bilayers. Moreover, both synthetic and natural precursors of RSSH were recently found to inhibit ferroptosis in cells exposed to GPX4 or xCT inhibitors [243] or lacking GPX4 [231]. Additional observations support the notion that S^0^ species are endogenous ferroptosis inhibitors. Cysteine uptake can protect cells not just by supporting GSH biosynthesis, but also by providing sulfur for S^0^ synthesis. Specifically, overexpression of xCT increased intracellular persulfide levels and rescued GPX4-deficient cells [230]. Moreover, S^0^ species are observed to be upregulated under pro-ferroptotic conditions, suggesting that enhanced biosynthesis of persulfides is part of an adaptive response that protects cells against lipid peroxidation and its consequences [230].

##### 2.2.6.4 Ferroptosis is modulated by enzymes involved in S^0^ metabolism

Numerous studies have found that the two central enzymes of the transsulfuration pathway, CSE and CBS, can protect against ferroptosis. For example, both CSE and CBS protect MYCN-amplified neuroblastoma cells against ferroptosis [244]. Overexpression of CBS led to ferroptosis resistance in erastin-treated ovarian cancer cells [245], while depletion of CBS sensitized ferroptosis-resistant cells [246]. Observations like these are typically interpreted to indicate that the transsulfuration pathway provides an internal cysteine source for GSH biosynthesis. However, it is also possible that CSE and CBS counteract ferroptosis by generating H_2_S/S^0^. Indeed, the depletion of CBS in breast cancer cells lowered endogenous persulfide levels, without affecting overall GSH or cysteine levels, while sensitizing the cells to ferroptosis [247]. Moreover, another enzyme regulating persulfide levels independently of the transsulfuration pathway, persulfide dioxygenase, was observed to influence ferroptosis sensitivity [230]. A potential anti-ferroptotic role of 3-mercaptopyruvate sulfurtransferase, an enzyme generating persulfides independently of the transsulfuration pathway, remains to be further explored.

The persulfide-based radical scavenging system is probably inherited from the last common ancestor of all forms of life, as persulfides are seen to be produced in archaea, bacteria and eukaryota. Persulfides appear to be more abundant than the long-known radical scavengers ascorbate and tocopherol, which humans need to acquire through their diet. Pharmacological manipulation of persulfide metabolism may have biomedical applications. On the one hand, inhibition of persulfide production pathways may be useful as a strategy to promote ferroptosis in cancer cells. On the other hand, enhancement of persulfide production may inhibit ferroptosis in neurons, potentially slowing their degeneration.

#### 2.2.7 Selenium

Selenium, an essential trace element, plays a crucial role in maintaining redox homeostasis. Selenium is highly reactive due to its high energy electrons, wich are key to its unique redox reactivity [248]. The biological function of selenium is mediated by selenoproteins, which contain the 21st proteinogenic amino acid selenocysteine, a cysteine analog that possesses selenium instead of sulfur [249]. Twenty-five unique selenoproteins have been discovered in humans, including five types of GPXs, three types of thioredoxin reductases, and selenoprotein P in plasma [249,250].

##### 2.2.7.1 Selenium/selenocysteine metabolism

**Fig. 8:**
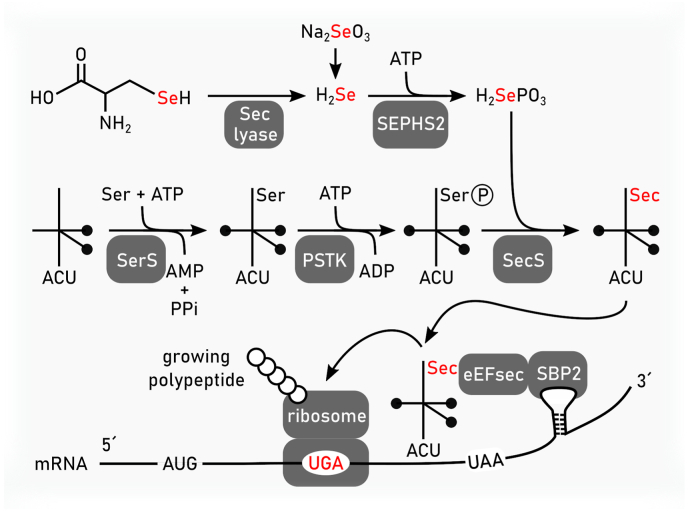
**Selenium metabolism and selenoprotein synthesis.** Selenocysteine (Sec) is a unique amino acid synthesized on transfer RNA (tRNA) by incorporating inorganic selenium. Initially, Sec is converted by the enzyme Sec lyase (SCLY) to selenide and then further delivered to selenophosphate by SEPHS2. Selenite and other inorganic forms of selenium can also enter this pathway through cellular reduction processes. Concurrently, Serine (Ser) is bound to tRNA[Ser]Sec, which harbors the UGA anticodon, by seryl-tRNA synthetase (SerS), followed by phosphorylation facilitated by phosphoseryl-tRNA kinase (PTSK). Sec is then produced on tRNA by Sec synthase (SecS/SEPSECS). During translation, Sec is incorporated into selenoproteins via the Sec-insertion sequence (SECIS), a stable loop structure located in the 3′-UTR of selenoprotein mRNA. The complex formed by Sec-tRNA[Ser]Sec and associated factors, such as eukaryotic elongation factor for Sec translation (eEFSEC) and SECIS binding protein 2 (SBP2/SECISBP2), facilitates the translation process. At the UGA codon, Sec-tRNA[Ser]Sec is supplied from the SECIS complex to ensure accurate incorporation of Sec into the growing peptide chain and preventing premature termination.

Selenocysteine is encoded by the stop codon “UGA". Unlike other amino acids, selenocysteine is synthesized on its cognate tRNA^[Ser]Sec^ (TRSP) using inorganic selenium [249,251] (Fig. 8). The synthesis of selenocysteine-loaded tRNA^[Ser]Sec^ commences with amino acylation of serine to tRNA^[Ser]Sec^ by seryl-tRNA synthetase, followed by phosphorylation of the hydroxyl group of Ser by phosphoseryl-tRNA kinase. Selenophosphate synthetase 2 (SEPHS2) phosphorylates inorganic selenium, generating selenophosphate. Subsequently, in the presence of selenophosphate and phospho-serine-loaded-tRNA^[Ser]Sec^, selenocysteine synthetase (Sep (O-Phosphoserine) tRNA: selenocysteine tRNA, SEPSECS) catalyzes the formation of the selenol group, completing the synthesis of selenocysteine on its tRNA. Notably, SEPHS2 itself is a selenoprotein, suggesting its involvement in the self-regulation of selenocysteine synthesis.

The supply of inorganic selenium for seleneocysteine synthesis is derived from dietary intake. Selenium is absorbed from the digestive tract and enters distinct biosynthetic pathways, depending on its chemical form [250,252]. When selenocysteine is obtained from ingested food, it is metabolized by selenocysteine lyase. On the other hand, inorganic selenium, such as sodium selenite, can also enter cells [253], undergo reduction, and contribute to selenocysteine synthesis. Selenomethionine, a methionine analog that contains selenium instead of sulfur, serves as one of the primary sources of selenium absorbed by the body. Selenomethionine is metabolized similarly to methionine and can be directly incorporated into proteins. In some cases, selenomethionine is converted to selenocysteine by the transulfuration pathway and subsequently enters the selenocysteine synthesis pathway through the action of selenocysteine lyase.

The successful decoding of UGA as a selenocysteine necessitates the presence of a stable stem-loop like structure, called the SECIS (selenocysteine-insertion sequence) element, present in the 3′UTR of selenoprotein mRNAs [249,254] (Fig. 8). A protein-RNA complex is formed with the SECIS element, involving SECIS-binding protein 2 (SBP2), eukaryote elongation factor for selenocysteine translation, several other factors and selenocysteine-tRNASec [255]. When the ribosomes encounter the UGA codon, only the interaction of this complex with the ribosomes and selenocysteine-tRNA^[Ser]Sec^ leads to successful incorporation of selenocysteine into the nascent polypeptide chain. The SECIS element plays a crucial role in maintaining the stability of selenoprotein mRNA. In the absence of SBP2 and selenocysteine-tRNASec, selenoprotein mRNA is subject to degradation through nonsense-mediated mRNA decay [254,256]. The affinity between SBP2 and SECIS is not equal and varies among different selenoproteins. For instance, the SECIS elements of GPX4 and Trx reductase (TrxR) exhibit a strong affinity for SBP2, while the affinity of GPX1 is comparatively lower. In conditions of selenium deficiency when selenocysteine-tRNA^[Ser]Sec^ levels are low, the biosynthesis of a subset of selenoproteins is suppressed, and instead, selenium is preferentially utilized for the biosynthesis of GPX4 and TrxR. This differential utilization ensures that the limited selenium resources are allocated efficiently to selenoproteins with higher affinity for SBP2, thus maintaining essential cellular functions under selenium starvation conditions.

##### 2.2.7.2 Selenium metabolism and ferroptosis

Selenium metabolism is closely related to the regulation of ferroptosis because the cellular content of selenocysteine-tRNA^[Ser]Sec^ is one of the limiting factors for GPX4 synthesis [10]. Selenium-deficient conditions can induce lipid peroxidation and ferroptotic cell death, and combined deficiencies of both selenium and vitamin E exacerbates susceptibility to ferroptosis *in vitro* and *in vivo* [10,257]. Further, defects in selenoprotein synthesis are associated with lethal phenotypes, as observed in GPX4 and selenocysteine-tRNASec KO mice [23,258]. On the other hand, patients with defects in selenoprotein synthesis, such as the ultrarare mutations in SBP2 and TRSP, have been described [259,260]. Patients who harbor mutations in selenoprotein synthesis show decreased levels of several selenoproteins and have characteristic abnormalities in thyroid hormone levels with high thyroxine T_4_ and low triiodothyronine T_3_ as well as short stature during childhood and bone maturation delay due to the decrease in selenoenzyme iodothyronine deiodinases. In these patients, increased lipid peroxidation and protective effects of vitamin E have been reported, which are likely linked to the decrease of GPX4. A complex phenotype, including photosensitivity, azoospermia, axial muscular dystrophy, abnormal mononuclear cell cytokine secretion, and telomere shortening, has also been reported [259,261]. The complex phenotype of selenium deficiency is likely a result of the varying degree of selenoprotein loss observed in the different model system studied. Notably, activation of the antioxidative transcriptional factor NRF2 has been reported in *Trsp* KO mice, likely as a compensatory mechanism [262].

Plasma selenocysteine-containing protein selenoprotein P (SELENOP) plays a pivotal role in selenium-metabolism via its function as a selenium-transporter to maintain selenoenzyme expression in tissues with high selenium demand, such as the brain, kidney and testis [263,264]. SELENOP is mainly synthesized in the liver and contains up to 10 selenocysteine residues and is secreted into the plasma. SELENOP uptake in target cells and tissues depends on its receptors, such as apolipoprotein E receptor 2 (APOER2/LDL Receptor Related Protein 8, LRP8) and megalin (LDL Receptor Related Protein 2, LRP2) [255,265]. Similar phenotypes between SELENOP KO mice and LRP8 KO mice have been reported in brain and testis, indicating the biological significance of receptor-mediated uptake of selenium in the form of SELENOP in these tissues. For instance, the severe phenotype of SELENOP KO mice in spermatogenesis and the well-established essential role of GPX4 for sperm development also suggests a direct link of SELENOP to maintain GPX4 expression in testis. In addition to the liver, SELENOP-expressing cells were found in several tissues. SELENOP expression in the brain is important for maintaining brain selenium and selenoproteins via the so-called SELENOP cycle, which has been demonstrated by liver-specific-SELENOP KO mice [264,266]. In the case of cells employing the SELENOP cycle to maintain cellular selenoproteins including GPX4, the decrease of SELENOP uptake is sufficient to cause ferroptosis-like cell death [253].

The close relationship between selenium metabolism, selenoprotein expression and defense against lipid peroxidation corroborates the importance of selenium metabolism in ferroptosis.

#### 2.2.8 Membrane permeabilization and calcium signaling in ferroptosis

Damage to plasma membrane integrity during ferroptosis is accompanied by alterations in ion homeostasis that disrupt the physiologically steep concentration gradients between the intracellular and extracellular medium. Among physiologically-relevant ions, calcium is of special interest because of its central role in regulating multiple signaling processes, including cell death pathways, as well as in the activation of membrane repair mechanisms.

Increased cytosolic calcium is recognized as one of the hallmarks of ferroptosis. It is mediated by osmotic forces and is associated with the formation of nanopores in the plasma membrane [267]. These conclusions were based on studies using the osmoprotectant polyethyleneglycol (PEG) 8000, which, when added to cells, is not able to enter through plasma membrane pores occurring during ferroptosis, thereby counterbalancing the high intracellular osmotic pressure. Accordingly, addition of PEG 8000 prevented water influx, cell collapse, as well as the increase in cytosolic calcium, indicating that there is a connection between alterations in cellular osmotic pressure and calcium fluxes in ferroptosis. Given the small size of calcium ions, it is possible that they enter ferroptotic cells not only through ferroptotic nanopores, but also by transient changes in the plasma membrane permeability. Simultaneously tracking of the kinetics of calcium fluxes and cell death at the single-cell level showed that, upon treatment with RSL3, a sustained increase in cytosolic calcium precedes complete plasma membrane rupture [267]. In contrast, treatment with erastin triggered two spikes in cytosolic calcium: 1) an early calcium increase, possibly associated with the inhibition of the voltage-dependent anion-selective channel proteins (VDACs) 2 and 3 [268]; and 2) a secondary increase in cytosolic calcium that was related to membrane damage and that could be inhibited by ferrostatin-1 [267]. Therefore, sustained increase in cytosolic calcium can be considered an early indicator of membrane permeabilization in ferroptosis. Another study showed that calcium signals and ferroptotic cell death can spread within cultured cells following a wave-like pattern [269]. In this case, treatment with PEG 1450 protected cells from membrane rupture, but failed to affect the wave-like spreading of lipid peroxidation nor calcium fluxes. This supports the hypothesis that wave-like propagation of lipid peroxidation, membrane damage and subsequent calcium fluxes do not require complete cell lysis [269].

So far, most evidence regarding the source of calcium fluxes in ferroptosis comes from studies on ferroptosis (at this time named glutamate-induced oxytosis). The increase in cytosolic calcium in oxytosis was initially attributed to influxes from the extracellular medium since cell death was inhibited in calcium-free medium [270]. However, this view has been challenged by findings showing a connection between lipid peroxidation and inhibition of calcium transport through ion channels. In this regard, the general inhibitor of calcium channels CoCl_2_ protected cells from oxytosis and ferroptosis [18,271]. Moreover, inhibitors of L-type and T-type voltage-gated calcium channels at the plasma membrane rescued cells from oxytosis [270]. Ruthenium red, an inhibitor of mitochondrial calcium uptake, also inhibited mitochondrial ROS production and cell death induced by glutamate, suggesting that mitochondrial calcium might contribute to ROS production in oxytosis [18]. Also, treatment with 2-aminoethoxydiphenyl borate, a specific inhibitor of the store-operated calcium entry, or the genetic inhibition of the calcium release-activated calcium channel protein (Orai) 1 conferred strong protection against oxytosis [272], as further supported by genetic downregulation of Orai1 and Orai3 protecting cells against ferroptosis [273]. Mitochondrial calcium uptake 1 also plays an important role in ferroptosis as it is required for mitochondrial calcium increase, mitochondrial hyperpolarization and lipid peroxidation in cold-stress induced ferroptosis [93]. Suppression of Ca^2+^ release from ER-mediated by ryanodine receptor channels was further shown to protect cells from ferroptosis [274]. Altogether, these studies suggest that both extracellular and intracellular calcium, and in particular cellular calcium stores in the ER and mitochondria, contribute to ferroptotic cell death. However, whether the increase in cytosolic calcium represents an early event important for proper functioning of the cellular machinery that controls ferroptosis induction or a late event consequence of plasma membrane damage durin ferroptosis execution are key open questions in the field.

Membrane damage in ferroptosis has been associated with the activation of the endosomal sorting complex required for transport III (ESCRT-III)-dependent membrane repair machinery [267,275]. ESCRT-III downregulation via the knockdown of the components charged multivesicular body proteins (CHMPs) 4B, 5, or 6 sensitized cells to ferroptosis [267,275]. Also, CHMP4B, which is usually present as a homogenous cytosolic distribution in healthy cells, reorganized into punctae upon ferroptosis induction [267]. Of interest, the cell-by-cell confocal analysis revealed that CHMP4B puncta formation kinetically coincided with the increase in cytosolic calcium, suggesting that calcium signaling and membrane repair are two interconnected processes in ferroptosis [267]. In this regard, the treatment with PEG 8000 not only inhibited calcium fluxes, but also CHMP4B puncta formation in ferroptotic cells. Plasma membrane permeabilization during ferroptosis causes the release of otherwise confined intracellular components to the extracellular space. The time between the initiation of membrane damage and cellular collapse affects the communication of cells with their microenvironment. As a result, modulation of the kinetics of ferroptosis through the activation of membrane repair mechanisms may impact the nature and quantity of intracellular factors that are released from dying cells. In line with this, ESCRT-III activation in ferroptosis modulates cytokine secretion to the extracellular medium and the activation of immortalized bone marrow-derived macrophages [267].

**Fig. 9:**
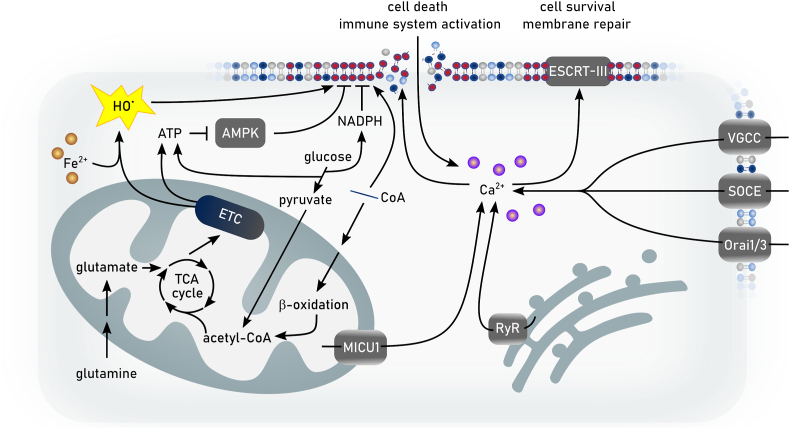
**Calcium signaling and energy metabolism in ferroptosis.** Increased cytosolic calcium is one of the hallmarks of ferroptosis. It remains unclear, however, whether calcium ions enter the cell through membrane nanopores, through endogenous channels at the plasma membrane, or whether the ER or mitochondria are responsible for calcium transport, or a combination of those. Cytosolic calcium contributes to ferroptosis and to the subsequent release of intracellular components. Yet, calcium signaling also connects plasma membrane damage with the activation of membrane repair mechanisms that counterbalance cell death. Energetic metabolism centered around mitochondrial activity is the major source for the generation of free radicals and reactive oxygen species in the cell. Therefore, energetic metabolism plays a central role in ferroptosis induction. Related processes, such as glutamine anaplerosis, TCA cycle and eletron transfer chain (ETC) activity, have all been demonstrated to be involved in ferroptosis. AMPK: AMP-activated protein kinase, ESCRTIII: endosomal sorting complex required for transport III, MICU1: Mitochondrial calcium uptake 1, ORAI: calcium channel protein, RyR: ryanodine receptor, SOCE: store-operated calcium entry, VGCC: voltage-gated calcium channels.

Accumulating evidence suggests a model in which increased cytosolic calcium constitutes an early event occurring before downstream lipid peroxidation and prior to cell death (Fig. 9). The time between the rise in cytosolic calcium and cell death might represent a time window where calcium signaling can be exploited to either mediate membrane repair or to facilitate cell death. Thereby, ESCRT-III plays a central role in these processes, as it is activated in a calcium-dependent manner to repair membrane damage, counterbalance cell death, and modulate the immunological signature of ferroptotic cells. Whether membrane repair mechanisms other than ESCRT-III are activated in ferroptosis in response to membrane damage and calcium remains an open question that requires further study.

#### 2.2.9 Energy metabolism and ferroptosis

Mammalian cells predominantly rely on glycolysis and OXPHOS as their main ATP production pathways. Glycolysis, occurring in the cytosol, metabolizes glucose into pyruvate to yield 2 molecules of ATP. Under anaerobic conditions, pyruvate is primarily reduced to lactate by lactate dehydrogenase A to uphold the NAD^+^/NADH redox balance. In contrast, under aerobic conditions, pyruvate enters the mitochondria to undergo oxidation to form acetyl-CoA. This marks the initiation of the TCA cycle and OXPHOS, leading to the potential generation of up to 36 molecules of ATP. Remarkably, the TCA cycle and OXPHOS can also be fueled by sources other than glucose, including glutamine and fatty acids. An intriguing hallmark of cancer cells is their heightened use of glucose for aerobic glycolysis (Warburg effect). Notably, the ratio between glycolysis and OXPHOS in contributing to total ATP yield exhibits variability across different cell types, growth phases, and microenvironments. In addition to ATP synthesis, the intermediates originating from glycolysis and the TCA cycle play a pivotal role in the synthesis of macromolecules like nucleic acids, lipids, and proteins. These molecules are essential for the vigorous growth and proliferation of cancer cells. Importantly, energy metabolism not only generates free radicals as initial triggers of ferroptosis, but also fundamentally impacts on cellular susceptibility to ferroptosis through diverse mechanisms as discussed below.

##### 2.2.9.1 Mitochondrial OXPHOS-generated superoxide in ferroptosis induction

While glycolysis plays a major role in cancer energy metabolism, a substantial number of cancers opt for OXPHOS either as a primary energy production route or in conjunction with glycolysis. Notably, mitochondrial OXPHOS represents a significant source of cellular ROS [276]. Specifically, electron leakage from ETC complexes I and III yields superoxide, which subsequently converts to hydrogen peroxide through the activity of SOD or by spontaneous dismutation. Protonated superoxide (the hydroperoxyl radical) or hydroxyl radicals arising from the Fenton reaction of H_2_O_2_ with labile iron, may then initiate the peroxidation of PLs containing PUFAs. Intriguingly, glutamine metabolism serves to support the mitochondrial TCA cycle, thereby intensifying the generation of mitochondria-derived ROS, which in turn play a pivotal role in M) ferroptosis [184]. Moreover, metabolite intermediates from the TCA cycle, including α-ketoglutarate, fumarate, succinate, and malate, can effectively substitute for glutamine and induce CDI ferroptosis [277]. Interestingly, the application of various ETC complex inhibitors or mitochondrial uncoupling agents has been shown to effectively impede CDI ferroptosis [277]. This underscores the critical role of electron transport and proton pumping within mitochondria in the initiation of ferroptosis (Fig. 9). Importantly, loss-of-function mutations of fumarase, an enzyme in TCA cycle, can make cancer cells more resistant to CDI ferroptosis, thus conferring tumorigenic advantage [277]. However, it is important to note that the TCA cycle and OXPHOS are not prerequisites for ferroptosis induced by decreased GPX4 activity, suggesting that under this condition, modest free radical production independent of OXPHOS could be sufficient to induce overwhelming phospholipid peroxidation [277]. Treatment with MitoQ [278] or the nitroxide antioxidant XJB-5-131 [279], which are mitochondria-targeted versions of these lipophilic antioxidants, were found to significantly rescue cells from undergoing ferroptosis. However, mitochondria-targeted quinones are less effective than untargeted quinones [6], whereas mitochondria-targeted nitroxides are almost as effective as untargeted nitroxides [280].

While fatty acid beta oxidation can contribute to mitochondrial OXPHOS, it generally exerts a suppressive effect on ferroptosis likely by limiting the availability of unesterified PUFAs. 2,4-Dienoyl-CoA reductase 1 (DECR1), which is involved in PUFA beta-oxidation in the mitochondria, is notably overexpressed in prostate cancer. Knocking out DECR1 induces ER stress and sensitizes castration-resistant prostate cancer cells to ferroptosis both *in vitro* and *in vivo*. Furthermore, inhibiting beta-oxidation serves to enhance ferroptosis in cancer cells [281,282].

##### 2.2.9.2 Ferroptosis regulation within mitochondria

Compelling evidence supports the importance of mitochondria in driving CDI ferroptosis, hence it is reasonable to assume that there might be mitochondria-localized defense systems to prevent ferroptosis. A recent study suggested that in addition to the mitochondrial form of GPX4, dihydroorotate dehydrogenase (DHODH), an inner mitochondrial membrane-localized enzyme involved in pyrimidine synthesis can reduce CoQ_10_ to CoQ_10_H_2_ [283]. The inhibition of DHODH by brequinar accordingly sensitizes cancer cells towards ferroptosis via detoxifying mitochondrial lipid peroxidation [283]. However, another study posits that brequinar's sensitizing effect on ferroptosis is actually accomplished via FSP1 inhibition and not via DHODH [284], as in the earlier study exceedingly high concentrations of brequinar were used, which easily inhibit FSP1. The latter study also claimed that cytosolic GPX4, which for yet unknown reasons can also enter the mitochondrial intermembrane space in somatic cells, but not the mitochondrial form of GPX4, which localizes to the mitochiondrial matrix, is responsible for the suppression of mitochondria-related ferroptosis [284].

Additionally, mitochondrial outer membrane protein FUN14 domain containing 2 (FUNDC2) has been recognized as a ferroptosis promoter through its interaction with the mitochondrial GSH transporter SLC25A11, which in turn negatively regulates mitochondrial GSH levels [285]. Moreover, FUNDC2's involvement in ferroptosis has been demonstrated in conditions such as cardiomyopathy induced by the anthracycline doxorubicin [285].

##### 2.2.9.3 Cellular energy status links fatty acid synthesis to ferroptosis sensitivity

AMP-activated protein kinase (AMPK) acts as a pivotal sensor of cellular energy levels. Recent research has revealed that instances of energy depletion, such as glucose starvation, trigger the activation of AMPK. This, in turn, suppresses the synthesis of certain PUFAs and subsequently inhibits ferroptosis by phosphorylating and deactivating acetyl-CoA carboxylase, a rate-limiting enzyme in fatty acid biosynthesis. Conversely, the inactivation of AMPK augments susceptibility to ferroptosis [286,287]. Intriguingly, cancer cells exhibiting loss-of-function mutations in AKT, a tumor suppressor and an upstream activator of AMPK, manifest elevated basal AMPK activity, consequently displaying heightened resistance to ferroptosis [287]. Conversely, SLC2A1 (glucose transporter 1)-mediated glucose uptake fosters glycolysis, pyruvate oxidation, TCA cycle and fatty acid synthesis, thereby ultimately facilitating ferroptosis in cancer cells [288]. Besides glycolysis, glucose is also shunted to the PPP to generate NADPH. Since NADPH plays a crucial role in maintaining cellular redox homeostasis, glucose metabolism can also play a ferroptosis-suppressing function in a context dependent manner.

Given their diverse origins, growth stages, and microenvironments, cancer cells exhibit heterogeneous energy metabolism phenotypes (see chapter 4.1). The glycolytic phenotype in cancer cells primarily emerges due to factors such as hypoxia and gene reprogramming driven by oncogenes, such as Ras and Myc. Suppression of glycolysis in cancer cells leads to the shift of energy metabolism from glycolysis to mitochondrial respiration. Since OXPHOS-generated superoxide play a pivotal role in CDI ferroptosis, this raises several intriguing questions: Does CDI ferroptosis first originate within mitochondria? Does the ratio of glycolysis to OXPHOS determine sensitivity to ferroptosis? Moreover, can we manipulate OXPHOS in such a way that ferroptosis in cancer cells is selectively potentiated?

## 3 Regulation of ferroptosis

### 3.1 Regulation via epigenetics and long non-coding RNA

Epigenetic regulation is based on (de-)methylation, (de-)acetylation, and (de-)ubiquitination of DNA and histones, as well as non-coding RNAs. Epigenetic regulation of ferroptosis has mainly been investigated in the context of cancer progression, as summarized elsewhere [289]. DNA methylation usually generates 5-methyl-cytosine in GpG island and seemingly affects transcription of genes involved in all aspects of ferroptosis. The promotor of the gene driving expression of the *Gpx4* gene for example is less methylated in cancer cells contributing to higher levels of the protein in these cells [290], whereas elevated methylation of GpG islands of *Gpx4* and *FSP1* leads to diminished protein levels [291]. Both methylation as well as acetylation of histones regulate susceptibility of cells towards ferroptosis [292,293], e.g. acetylation of K27 on histone 3 of the *Gpx4* encoding gene increased its transcription [293].

On the mRNA level, *FSP1* [294] and *SLC7A11* [295] are stabilized by N-acetyltransferase 10-induced ac4C acetylation. The most relevant mRNA modification is the formation of *N*^6^-methyladenine. mRNAs of *Gpx4*, *SLC7A11*, and *FSP1* are targeted by *N*^6^-methyladenine formation leading to decreased expression and increased ferroptosis [296]. Non-coding RNAs have been shown to affect ferroptosis as well. Several miRNAs bind to the mRNAs of *Gpx4*, *SLC7A11*, and other ferroptosis-related proteins [297] leading to their degradation and thereby enhanced or impaired ferroptosis, respectively. Moreover, ncRNAs affect ferroptosis by targeting proteins responsible for lipid, redox, and iron homeostasis [297]. Several long non-coding RNAs are associated with regulation of ferroptosis and specific diseases making them potential prognistic tools, such as the 36 lncRNAs that were linked to ferreoptosis as well as progression of bladder cancer [298].

### 3.2 Redox regulation and ferroptosis protection by KEAP1-NRF2 system

As an iron-dependent cell death associated primarily with excessive and aberrant peroxidation of membrane PLs, ferroptosis is intricately linked to cellular redox status. In turn, it is unsurprising that NRF2, the major regulator of the cellular antioxidant response, is closely related to this oxidative cell death. In fact, many of the processes that protect cells from ferroptosis are directly or indirectly under the regulation of NRF2, such as cystine uptake, GSH and NADPH synthesis, iron sequestration and export, and synthesis of molecules containing sulfur catenation. Of note, several of these pathways are also directly required for the anti-ferroptotic function of GPX4 and FSP1, further highlighting the link between NRF2 and ferroptosis.

#### 3.2.1 KEAP1-NRF2 system

**Fig. 10:**
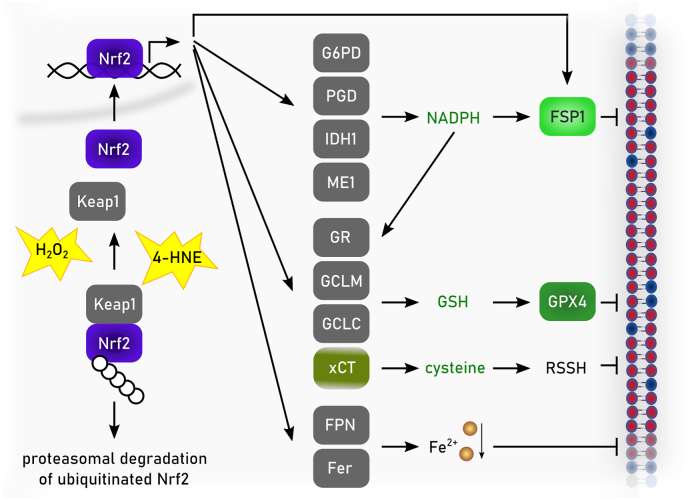
**NRF2 target genes involved in the ferroptosis regulation.** NRF2 is bound by KEAP1 and degraded in proteasome in unstressed conditions. Exposure to oxidative stress, such as hydrogen peroxide and 4-hydroxynonenal, leads to KEAP1 inactivation and NRF2 stabilization. NRF2 suppresses ferroptosis by promoting NADPH production, glutathione synthesis, and cystine uptake and by decreasing labile iron. The increased cystine uptake leads to increased sulfane sulfur production. GPX4 and FSP1 appear to be context dependent NRF2 target genes. GCLC and GCLM: catalytic and regulatory subunits of gamma-glutamylcysteine ligase, G6PD: glucose-6-phosphate dehydrogenase, IDH1: isocitrate dehydrogenase 1, ME1: malic enzyme 1, PGD: phosphogluconate dehydrogenase, RSSH: hydropersulfide, xCT: representing cystine/glutamate antiporter.

NRF2 is a potent transcription activator that coordinates expression of a plethora of antioxidant and cytoprotective genes [[299], [300], [301]]. Under homeostatic and physiological conditions, NRF2 is targeted for proteasomal degradation by KEAP1-mediated ubiquitination. However, when electrophiles and hydrogen peroxides react with and modify thiols of cysteine residues in KEAP1, its ubiquitination function is suppressed, allowing for the stabilization and nuclear translocation of NRF2 (Fig. 10). Put simply, KEAP1 is an electrophile sensor, and NRF2 is an effector that mediates cellular response. NRF2 has been reported to promote cell survival under oxidative stress via multiple mechanisms, including inflammation control [302], regulation of cell differentiation/proliferation [303], and extensive rewiring of redox metabolism [[304], [305], [306]]. Given its critical role in regulating redox homeostasis, NRF2 has begun to attract attention as an important suppressor of ferroptosis [307,308].

#### 3.2.2 NRF2 and GPX4/FSP1

NRF2 target genes include GSH biosynthesis genes. These include genes encoding the catalytic and regulatory subunits of γ-GCL (GCLC/GCLM), which form the rate-limiting enzyme for GSH synthesis, and GSH reductase. Additionally, import of cysteine (CysSH), a building block of GSH, is regulated by the transporter *SLC7A11*/xCT, which is also under NRF2 regulation. NRF2-dependent enhancement of CysSH uptake and GSH synthesis directly contributes to GPX4's anti-ferroptotic function, as GPX4 catalyzes the reaction of GSH with lipid hydroperoxides to form lipid alcohols. Additionally, SLC7A11 activity increases the influx of selenium into the cells and therefore boosts GPX4 translation [309].

NRF2 also contributes to support cellular NADPH supplies by directly regulating four key NADPH-producing enzymes: glucose-6-phosphate dehydrogenase, phosphogluconate dehydrogenase, IDH1, and malic enzyme 1 [310]. NADPH is a key reducing equivalent required for several redox reactions, including cystine import via xCT as well as GSH and Trx regeneration by GSH reductase and TrxR1, respectively. Furthermore, FSP1 is an oxidoreductase that consumes NAD(P)H in order to reduce CoQ_10_ and vitamin K, both of which serve as lipid RTAs to suppress ferroptosis [51,52]. Finally, in addition to providing sufficient reducing power for these ferroptosis-suppressing effectors, NRF2 is considered to directly promote expression of some GPX family members and potentially FSP1, albeit in a context-dependent manner [98,311].

NRF2's involvement in iron metabolism is also notable [312]. NRF2 directly regulates genes encoding ferritin heavy/light chains (FTH/FTL) and FPN1 for iron sequestration and export, respectively [307]. Thus NRF2-activated cells have lower labile iron levels, thereby protecting cells from ferroptosis. However, NRF2 was also recently described to promote ferroptosis via increased HO-1 expression, which subsequently resulted in heme degradation and release of labile iron that could promote lipid peroxidation and ferroptosis [313]. Therefore, the role of NRF2-mediated iron metabolism in ferroptosis protection is likely dynamic and context-specific and warrants further investigation.

#### 3.2.3 NRF2 and sulfane sulfur species

Sulfane sulfur species have been found to protect cells from oxidative stress, control inflammation, and regulate mitochondrial energy metabolism [314,315]. Recent studies have indicated that hydropersulfides (RSSH), can suppress ferroptosis by scavenging lipidperoxyl radicals [230,240] (see chapter 2.2.6). NRF2 is thought to indirectly enhance supersulfide production by increasing cellular cysteine via xCT upregulation [316], so the radical scavenging by supersulfides represents another downstream mechanism of NRF2-mediated ferroptosis prevention.

NRF2 is considered a multimodal anti-ferroptotic regulator, which increases cellular reducing equivalents, regulates iron metabolism, and promotes sulfur utilization and metabolism (Fig. 10). Since ferroptosis underlies various pathological conditions, such as ischemia-reperfusion injury, stroke, and renal failure, activation of NRF2 may be a promising approach to ameliorate these pathologies. Furthermore, NRF2 hyperactivation in cancer is common and confers resistance to ferroptosis, which underlies therapeutic resistance and disease progression. The sensitization of cancer cells to ferroptosis as well as overcoming NRF2-mediated resistance to ferroptosis remains an exciting area of investigation to improve the efficacy of cancer treatments.

### 3.3 Posttranslational modifications as regulators of ferroptotic enzymes

**Fig. 11:**
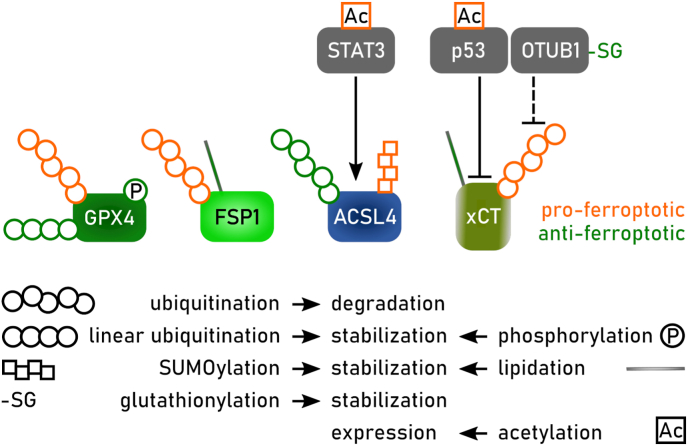
Regulation of ferroptosis-related proteins via posttranslational modifications. The activities or levels of several pro- and anti-ferroptotic proteins are regulated by (linear) ubiquitination, SUMOylation, glutathionylation, phosphorylation, lipidation, or acetylation ultimately leading to either increased (orange) or decreased (green) susceptibility towards ferroptosis. ACSL4: acyl-CoA synthetase long chain family member 4, FSP1: ferroptosis suppressor protein 1, GPX4: glutathione peroxidase 4, OTUB1: OTU deubiquitinase ubiquitin aldehyde binding 1, STAT3: signal transducer and activator of transcription 3, xCT: representing cystine/glutamate antiporter.

Intracellular processes and interactions are based on signal transduction, which can lead to posttranslational modifications of proteins affecting structure, localization, and activity. In light of the many publications dealing with ferroptosis, physiological regulation of this cell death mechanism remains only poorly studied. However, some modifications were already described as regulators of ferroptosis-related proteins (Fig. 11).

The small protein ubiquitin labels proteins for degradation by the proteasome system (see chapter 3.5). This is the main route to facilitate protein homeostasis. Some of the main proteins related to ferroptosis - GPX4 [317], FSP1 [318], ACSL4 [319], and SLC7A11 [320] – are subject to ubiquittination. The subsequent degradation of the respective proteins affects ferroptosis in several ischemia-related clinical contexts including ischemic stroke, kidney transplantation, or myocardial injury. In contrast, linear ubiquitination by the HOIL-interacting protein (HOIP) stabilizes GPX4 [321]. HOIP is part of the linear ubiquitin chain assembly complex and deficiency of this complex renders cells more sensitive to ferroptosis.

Small ubiquitin-like modifier (SUMO) can be conjugated to lysine residues leading to stabilization of proteins as described for ACSL4. DeSUMOylation by the SUMO/sentrin-specific protease 1, leads to destabilization of ACSL4 and to inhibition of ferroptosis [322].

Another important process regulating protein homeostasis is autophagy. GPX4 can be labeled for degradation by chaperone-mediated autophagy via interaction with heat shock cognate 71 kDa protein [80]. This interaction is inhibited in response to S104 phosphorylation of GPX4 by CKB. This activity of CKB depends on another phosphorylation of CKB T133 as a result of activation of serine/threonine kinase 1 via insulin-like growth factor 1 receptor signaling. Protein phosphorylation is one of the most studied posttranslational modifications and describes a covalent bond between a phosphate and either a serine or a tyrosine. ACSL4 is activated upon PKCβII-dependent phosphorylation [137]. Activated ACSL4 increases the concentration of PUFA-containing PLs, consequently increasing ferroptosis sensitivity. SLC7A11 is indirectly regulated by phosphorylation. AMPK-mediated phosphorylation at serines 90, 93, and 96 of Beclin1, a protein known for its central role in autophagy, is required for the formation of a Beclin1-SLC7A11 complex resulting in inhibition of xCT [323].

Acetylation, another extensively studied posttranslational modification, involves the ε-amino group of lysine and affects the expression of two ferroptosis related genes. Acetylated signal transducer and activator of transcription 3 increases expression of ACSL4 [324], whereas acetylation of p53 K383 decreases SLC7A11 expression [325]. Crotonylation is another less studied form of lysine acylation. Hypo-crotonylation of methylenetetrahydrofolate dehydrogenase 1 is suggested to impair ferroptosis by increasing protein levels of SLC7A11 and GPX4 as well as the GSH:GSSG ratios [326].

Lipidation summarizes the binding of different lipids to proteins. Myristic acid is such a lipid causing lipidation via covalent binding of a myristic acid-derived myristoyl group to glycine. Myristoylation of FSP1 localizes FSP1 to the cell membrane allowing the recovery of oxidized CoQ_10_ [50,51]. ACSL1 was mentioned as a regulator of the myristoylation of FSP1 [327]. Palmitic acid covalently bound to cysteines forms S-palmytoylation, a modification found at SLC7A11. S-palmytoylation diminishes ubiquitination of SLC7A11, thereby stabilizes and increases xCT levels, and provides increased resistance against ferroptosis in glioblastoma cells [328].

Oxidative posttranslational modifications can be reversible or irreversible. It was shown that a U46C variant of GPX4, but not the selenocysteine-containing wildtype enzyme, is highly susceptible to peroxide-induced overoxidation (i.e. sulfonylation) and irreversible enzyme inactivation, indicating that selenocysteine utilization in GPX4 protects against this irreversible oxidative modification [72]. Glutathionylation is a reversible modification, where GSH binds to a protein thiol and may indirectly affect ferroptosis. For instance, upon glutathionylation, the deubiquitinase OTU deubiquitinase ubiquitin aldehyde binding 1 (OTUB1) can interact with ubiquitin-conjugating enzyme E2 D1 and block ubiquitination of SLC7A11 by this enzyme. De-glutathionylation of OTUB1 by Grx1, in turn, leads to destabilization of xCT [320].

As mentioned above, the functions of many key ferroptotic enzymes emerge to be modulated by a variety of posttranslational modifications, although this research area is surprisingly poorly investigated so far. As such, future studies are needed to identify physiological regulators that ultimately modulate ferroptosis sensitivity and that in turn can represent new targets for pharmacological intervention.

### 3.4 Crosstalk with the immune system

#### 3.4.1 Ferroptosis in immune cells

**Fig. 12:**
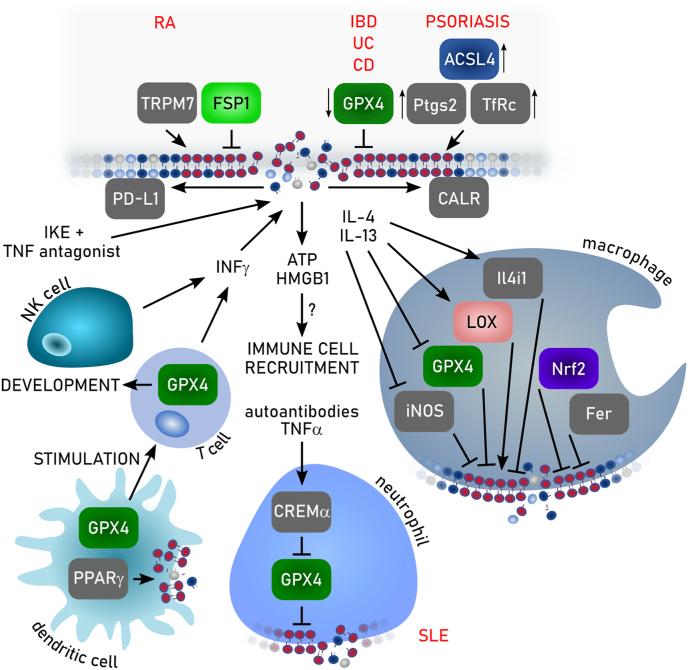
**Ferroptosis and immunity.** Ferroptosis is induced in diseases such as rheumatoid arthritis (RA) via transient receptor potential cation channel, subfamily M, member 7 (TRPM7) and ferroptosis suppressor protein (FSP1), inflammatory bowel disease (IBD), ulcerative colitis (UC), Crohn's disease (CD) via decreasing glutathione peroxidase 4 (GPX4), and psoriasis via fatty acid-CoA ligase 4 (ACSL4), prostaglandin-endoperoxide synthase 2 (Ptgs2) and transferrin receptor (TfRc). Thereby, ferroptosis leads to exposure of programmed cell death ligand-1 (PD-L1) and calreticulin (CALR) as well as release of ATP and high mobility group box 1 (HMGB1) which facilitate immune cell recruitment. As potential therapeutic inducers of ferroptosis IKE in combination with TNF antagonists are discussed. In addition, interferon (INF) γ released by natural killer NK cells and T cells, which relay on GPX4 during development, contributes to ferroptosis induction. Ferroptosis is mediated in dendritic cells by peroxisome proliferator-activated receptor (PPAR) γ. During systemic lupus erythematosus (SLE) ferroptosis is facilitated by autoantibodies and TNFα which induce cAMP-responsive element modulator (CREM) α, a transcriptional repressor for GPX4, in neutrophils. In macrophages interleukin (IL) −4 and −13 contributes to ferroptosis by inducing lipoxygenases (LOX) and blocking GPX4 and inducible nitric oxide synthase (iNOS). Nevertheless, it inhibits ferroptosis via l-amino acid oxidase IL-4 induced gene-1 (IL4iL). Further, nuclear factor erythroid-2-related factor 2 (NRF2) and ferritin (Fer) protect macrophages from ferroptosis.

Immune cells coordinate innate and adaptive immune responses that range from cell proliferation to death. They also appear as targets for ferroptosis, which impacts their maturation, functionality, and survival [329] (Fig. 12).

##### 3.4.1.1 Macrophages

In murine peritoneal macrophages RSL3 blocks pro-inflammatory responses upon lipopolysaccharide treatment by inducing NRF2, which inhibits the recruitment of RNA polymerase II to the transcription start site of several cytokines [330]. At the same time, activation of NRF2 also explains their resistance to ferroptosis. Also, in primary human macrophages, RSL3 activates NRF2, thereby promoting iron export through enhanced ferroportin expression [331]. Activation of NRF2 upon oxidative stress is well established and emerges as a powerful anti-ferroptotic mechanism in macrophages. In a tumor context macrophages are recruited by the tumor and support progression which make them to an interesting target during cancer therapy. Here, induction of ferroptosis in macrophages might be a possibility and future studies might need to inhibit their antioxidant capabilities in combination with ferroptosis inducers. Human macrophages also express high amounts of mitochondrial ferritin following RSL3 treatment by decreasing ferritinophagy [332]. Ferritins store iron and thus protect cells from iron-evoked oxidative stress. Accordingly, knock-down of mitochondrial ferritin and FTH increases the sensitivity of macrophages towards ferroptosis. Induction of ferritin by hypoxia also contributes to the protection from oxidative stress in macrophages and supports the observation that the cellular redox state and/or oxygen availability are crucial determinants of sensitivity towards ferroptosis.

##### 3.4.1.2 Neutrophils

Neutrophils react to infections by phagocytosis, degranulation, and neutrophil extracellular trap (NET) formation. NET formation is able to defend against invading microorganisms even after neutrophil death. NET generation is induced by sulfasalazine via non-enzymatic lipid peroxidation [333], but does not appear as a general response to ferroptosis inducers such as Erastin.

##### 3.4.1.3 T lymphocytes

In T cells GPX4 emerged to be crucial for the expansion and execution of an anti-viral defense but seems to be dispensable for their development in the bone marrow [86]. Specifically, it was demonstrated that GPX4 deficient mice display reduced numbers of CD8^+^ and CD4^+^ T cells [86]. Cystine and cysteine transporters are only marginally expressed on naïve T cells, but undergo a substantial increase upon their activation, which might be a compensatory mechanism to deal with increased levels of ROS resulting from elevated ana- and metabolic activities. Furthermore, IFN-γ, which is released by CD8^+^ T cells or natural killer (NK) cells, was shown to sensitize cancer cells towards ferroptosis [334]. This effect is linked to a decreased expression of SLC7A11 and consequently reduces GSH availability as well as increases mitochondrial ROS formation. Hence, an impaired anti-ferroptotic system in T cells might have general effects on immune responses [334].

##### 3.4.1.4 B lymphocytes

B cells are divided into B1, marginal zone, and B2 cells. Innate-like B cells (B1 and marginal zone cells) require GPX4 for their development, survival, and functional responses. Depletion of GPX4 in those cells increases lipid peroxidation and thus ferroptosis sensitivity. In contrast, B2 cells do not rely on GPX4 during development and function [89].

##### 3.4.1.5 Dendritic cells

RSL3-induced ferroptosis in murine dendritic cells (DCs) is regulated by peroxisome proliferator-activated receptor γ, which blocks maturation and consequently, DC function. Ferroptotic DCs lose the ability to produce cytokines and stimulate IFN production of CD8^+^ T cells [335].

#### 3.4.2 Ferroptosis of immune cells in type 2 immunity – a particular challenge

While the regulation of ferroptosis in immune cells is essential for homeostasis (see above), the control of this inflammatory and potentially damaging form of cell death is particularly challenging under type 2 inflammatory conditions such as asthma or helminth infection. Indeed, the hallmark type 2 cytokines IL-4 and IL-13 reduce the expression and activity of GPX4, while increasing 12/15- lipoxygenase [336] or the human ortholog 15- lipoxygenase [337], thus increasing ferroptosis susceptibility of immune cells (Fig. 12). Furthermore, inducible NOS, which can protect cells from ferroptosis [338], is downregulated during type 2 immune responses. Hence, under type 2 inflammatory conditions, multiple immune cell types, including group 2 innate lymphoid cells (ILC2), neutrophils, basophils, mast cells, eosinophils, M2 macrophages, B cells and T helper 2 cells are activated and expand in a ferroptosis-prone environment. Yet, how ferroptosis is regulated and how it may affect functionality in this diverse set of immune cells remains largely unknown. While innate lymphocytes such as NK cells can undergo ferroptosis in the context of cancer, ferroptotic cell death of ILCs in infectious or inflammatory settings remains largely unexplored. Neutrophil ferroptosis can contribute to immunosuppression in cancer [336], however whether type 2 cytokines, which promote tumor immune evasion, may support neutrophil ferroptosis is unclear. While studies assessing the ferroptosis susceptibility of mast cells and basophils are lacking, several recent reports suggest that eosinophils – another key cell type of type 2 immune responses – can undergo ferroptosis. Indeed, eosinophils show hallmarks of ferroptosis after treatment with ferroptosis-inducing agents (damaged mitochondria, reduced viability), however, treatment with ferrostatin-1 or liprocstatin-1 cannot rescue the cells from ferroptotic cell death, although it blocks the accumulation of lipid hydroperoxides [339]. Thus, inducing eosinophil ferroptosis may provide a therapeutic benefit in type 2 inflammatory settings such as allergic asthma or nasal polyposis. M2 (alternatively activated) macrophages currently represent the best-explored cell type with regard to ferroptosis susceptibility in the context of type 2 immune responses. M2 macrophages show a high susceptibility to undergo RSL3 induced ferroptosis *in vitro* [338], and these cells are sensitive to the lack of GPX4, causing ferroptotic cell death *in vivo*, e.g., during infection with a parasitic nematode [340]. IL-4-stimulated macrophages do not only upregulate the expression of the ferroptosis promoting enzyme 15- lipoxygenase, but also the l-amino acid oxidase IL-4 induced gene-1. The latter enzyme protects macrophages from ferroptosis by indole-3-pyruvate-dependent free radical scavenging and induction of an anti-oxidative gene expression program [341]. Thus, type 2 cytokines induce multiple factors that modulate ferroptosis susceptibility of macrophages, enabling a network that controls the potent effector functions of these cells in type 2 immune settings. While ferroptosis of M2 macrophages may fine-tune type 2 inflammation, anti-helminth host defense and tissue repair, ferroptotic death of T- or B-lymphocytes could further determine the strength and longevity of the adaptive immune response against allergens or helminth parasites. GPX4 is essential for protecting CD4^+^ T cells from ferroptosis and for efficient antibody responses during influenza vaccination [342]. However, whether ferroptosis of T or B cells is altered under type 2 inflammatory conditions and can determine, e.g. pathological IgE- or protective antibody responses in asthma or helminth infection, remains to be investigated. Future work should determine the functional roles of ferroptosis in the key immune cell types involved in type 2 immunity.

#### 3.4.3 Involvement of ferroptosis in autoimmune disease

Ferroptosis has been reported to be involved in various autoimmune disease scenarios (Fig. 12). Depending on the specific disease context, the occurrence of ferroptosis can be either pathogenic or therapeutic. In the following, the role of ferroptosis in different autoimmune diseases will be discussed.

##### 3.4.3.1 Systemic lupus erythematosus

Systemic lupus erythematosus (SLE) is a chronic autoimmune disorder, which is mainly characterized by the production of proinflammatory autoantibodies, leading to self‐attack of cells. The accelerated neutrophil death and deficiency in clearing dying neutrophils are the important factors promoting autoimmune responses in patients with SLE [343]. In a recent study, Li et al. reported that ferroptosis is the main form of neutrophil cell death in both lupus-prone mice and SLE patients [90]. Accordingly, liproxstatin-1 was shown to significantly ameliorate disease progression in the lupus-prone mouse model. Mechanistically, autoantibodies and IFN-α present in the serum suppress *Gpx4* expression in neutrophils through the transcriptional repressor cAMP-responsive element modulator α and sensitize neutrophils to ferroptosis. Of note, neutrophil-specific *Gpx4* haploinsufficiency recapitulates key clinical features of human SLE, suggesting the important contribution of ferroptosis to the development of SLE [90]. Additionally, B cells were also reported to be susceptible to ferroptosis in lupus patients and MRL/lpr mice. And liproxstatin-1 treatment increases CD19^+^ B cells while reducing plasma cells in the spleen, which is the potential source of autoantibodies driving SLE [344]. Treatment of patients exhibiting lupus nephritis, that often worsen SLE, with liproxstatin-2 diminished ferroptosis in proximal tubular epithelial cells [35] highligthing the therapeutic potential of suppressing ferroptosis in SLE.

##### 3.4.3.2 Rheumatoid arthritis

Rheumatoid arthritis (RA) is an autoimmune inflammatory disorder characterized by progressive joint destruction and a hyperplastic rheumatoid synovium with abnormal synovial fibroblasts proliferation. Recently, Wu et al. found that IKE can decrease fibroblast populations in RA synovium and modulate joint inflammation and tissue damage in collagen-induced arthritis mouse model. Two groups of fibroblasts were identified with distinct susceptibility to IKE-induced ferroptosis through scRNA-Seq. Mechanistically, a ferroptosis-resistant state of fibroblasts may arise from TNF signaling promoting cystine uptake and biosynthesis of GSH. A low dose of IKE along with TNF antagonist was sufficient to induce ferroptosis in fibroblasts and further attenuate arthritis progression [345]. Additionally, it has been shown that iron deposition in the synovial fluid induces macrophage ferroptosis and aggravates RA disease progression. Notably, liproxstatin-1 alleviates the progression of K/BxN serum-transfer-induced arthritis mice with an increase of the M2 macrophage population, which exhibits a higher susceptibility to ferroptosis than M1 macrophages in this disease context [346]. However, in another study, Zhou et al. reported that the elevated expression of transient receptor potential melastatin 7 promotes ferroptosis of chondrocytes during RA, which is tightly associated with the progression of RA [347]. These studies indicate that ferroptosis induction can be a potential therapeutic strategy of RA. However, the effects of ferroptosis induction in RA, can be complex and cell type-dependent.

##### 3.4.3.3 Inflammatory bowel disease

Inflammatory bowel disease, including ulcerative colitis (UC) and Crohn's disease, is a chronic inflammatory disorder of the gastrointestinal tract with progressive aggravation and high relapse rates [348]. Recent studies have reported the accumulation of iron, MDA and decreased GSH levels in UC colonic tissues. Additionally, an increase of *Acsl4* and decrease of *Gpx4* expression in intestinal epithelial cells (IECs) suggests that IECs undergo ferroptosis during UC. Furthermore, ferrostatin-1 and liproxstatin-1 treatment dramatically ameliorates the severity of dextran sulfate sodium-induced UC [[349], [350], [351]]. In contrast, the involvement of ferroptosis in the pathogenesis of Crohn's disease is less clear. A recent study reported that IECs in Crohn's disease indeed exhibit impaired GPX4 activity and an increase of 4-hydroxy-2-nonenal (4-HNE) immunoreactivity, indicating a vulnerability of IECs to ferroptosis in Crohn's disease [352], although the therapeutic potential of targeting ferroptosis for Crohn's disease remains elusive.

##### 3.4.3.4 Psoriasis

Psoriasis is a chronic inflammatory skin disease that features localized or widespread erythema, papules, and scaling. Recent studies have reported the involvement of ferroptosis in psoriatic lesions, with increased expression levels of *Acsl4*, *prostaglandin-endoperoxide synthase 2 (Ptgs2,* (encoding cyclooxygenase-2)) and *Tfrc* coinciding with decreased *Gpx4* levels. Moreover, ferrostatin-1 alleviated psoriasiform dermatitis induced in mouse models [353], whereas intradermal injection of RSL3 aggravated the development of psoriasis-like dermatitis [354].

In addition to the diseases discussed above, recent work has also revealed the involvement of ferroptosis in other autoimmune diseases, including autoimmune hepatitis [[355], [356], [357]] and multiple sclerosis [33,[358], [359], [360]], highlighting the potential of targeting ferroptosis as a new strategy for autoimmune disease therapy.

#### 3.4.4 Implications of ferroptosis in anti-tumor immunity

Antitumor immunotherapy, such as the use of immune checkpoint inhibitors, has shown tremendous success in the past decades across many types of cancers, but response rates are often still unsatisfactory and strategies to improve therapy efficacy are urgently needed [361]. The efficiency of immunotherapy relies on the induction of a lasting anti-tumor immune response to eliminate malignant cells and prevent recurrence [362]. Immunogenic cell death is a form of regulated cell death leading to the release of inflammatory mediators that will induce activation of immune cells and might therefore synergize with immunotherapy [363].

Ferroptotic cells release damage-associated molecular patterns (DAMPs), such as high mobility group box 1 (HMGB1) and ATP, and expose the ER protein calreticulin to the outer leaflet of the plasma membrane, which are hallmarks of Immunogenic cell death [363] (Fig. 12). However, the data about the actual immunogenicity of ferroptotic cell death is conflicting. Recently, it was shown that despite releasing DAMPs, cells dying from ferroptosis reduce DC maturation and inhibit the anti-tumor activity of DCs in prophylactic cancer vaccination models [364]. Coincubation of ferroptotic cancer cells was associated with transfer of lipid droplets to the dendritic cells, a condition known to interfere with antigen cross presentation [365]. By contrast, Efimova et al. used RSL3 to induce ferroptosis in murine fibrosarcoma or glioma cells and apparently found that cells displaying early ferroptosis would be able to induce bone-marrow derived DC activation and protection in a prophylactic vaccination setting [366]. It can not be excluded that the difference between Wiernicki et al. and Efimova et al. is the prophylactic vaccination outcome may be due to the incomplete cell death induction during prophylactic vaccination with early ferroptotic cells (genetic inducible model eventually leading to 100 % cells vs RSL3-induced leading to partial cell death by short treatment). Results from multiple other groups suggest that the use of ferroptosis inducers, in combination with immunotherapy, could improve treatment outcome [366]. Freire Boullosa et al. demonstrated that auranofin, an FDA-approved antirheumatic drug with anticancer properties, induces Immunogenic cell death in non-small-cell lung cancer (NSCLC) cell lines overexpressing mutant p53 [367]. This effect was shown to be caused by ferroptotic cell death induction through GPX4 downregulation, albeit auranofin is a bona fide TrxR inhibitor. Moreover, treatment of NSCLC cell lines with auranofin triggers the release of DAMPs such as HMGB1, ATP and calreticulin exposure to the plasma membrane and induces DC maturation as well. The use of auranofin also primes NSCLC to be eliminated by NK cells. In head and neck squamous cell carcinoma (HNSCC), spatial transcriptomic studies support the colocalization of tumor inflammation and ferroptosis signatures in the same histological regions of HNSCC [368]. Inducing ferroptosis in HNSCC cells by RSL3 or ferroptosis inducer (FIN) 56 triggers expression of programmed cell death ligand-1, creating a more inflamed tumor microenvironment that effectively respond to anti-programmed cell death ligand-1 therapy [369]. This encourages combining immune checkpoint inhibitors with ferroptosis inducers for treating HNSCC patients [368]. Along the same lines, apolipoprotein (APOE) L3 promotes ferroptosis and enhances CD8^+^TC antitumor immunity in colorectal cancer, both *in vitro* and in mouse models, partly through enhancing PD-1 blockade [370]. In gastric cancer, the high expression of FSP1 and CISD1, both suppressing ferroptosis, is correlated with an increased number of lymph node metastases [371]. Using database analysis, Zang et al. revealed that the high expression of ferroptosis suppressing genes in patients correlates with a decreased infiltration of CD8^+^TC and DCs. FSP1 is overexpressed in patients with hepatocellular carcinoma (HCC) as well [372]. Taken together, despite conflicting results in the literature, it is indisputable that the use of ferroptotic cell death holds great promises in the improvement of cancer immunotherapy.

### 3.5 Proteasomal degradation

Lysosomes and proteasomes are two major degradation hubs in eukaryotic cells. While the lysosome is an acidic organelle (pH ca. 4.5–5.0), which contains acidic-dependent hydrolases for degrading a variety of biomolecules, the proteasome is a large 26S, multi-catalytic protease complex. As a central part of the ubiquitin-proteasome system (UPS), proteasomes are responsible for the degradation of often short-lived, misfolded, and damaged proteins modified by K48-polyubiquitylation. Both lysosomal and proteasomal degradation are regulated by ubiquitination, cross-communicate, and are implicated in ferroptotic cell death. The broad impact of the lysosome and proteasome on ferroptosis is not only associated with the degradation of key pro- or anti-ferroptotic regulators, such as transferrin/lactotransferrin receptor or SLC7A11 and GPX4, but also, by their influence on cellular metabolism, oxidative stress, and iron homeostasis [373].

#### 3.5.1 Lysosomal-associated degradation and ferroptosis

The autophagy-lysosomal pathway (ALP) is involved in the degradation of various cytoplasmic materials and macromolecules including organelles, invading bacteria, long-lived proteins, lipids, carbohydrates, and nucleic acids. Blocking the ALP inhibits ferroptosis induced by canonical FINs, such as RSL3 or erastin [374], indicating that ALP sensitizes cells to ferroptosis. Part of these observations might relate to ferritinophagy, a process of autophagic degradation of ferritin, which accelerates ferroptosis execution in different cellular settings. Ferritin is a protein complex of 24 subunits which incorporates up to 4500 iron atoms, and thus, plays a key role in iron storage and detoxification. Ferritin is delivered to lysosomal degradation by NCOA4, which was identified as a ferritin binding protein and proposed to function as a selective cargo receptor, as its silencing inhibited ferritinophagy and ferroptotic cell death [375]. Under iron-replete conditions, NCOA4 undergoes ubiquitination by the HECT and RLD domain containing E3 ubiquitin protein ligase 2 and subsequent proteasomal degradation as a negative feedback mechanism, thus highlighting the crosstalk between ALP and UPS. In addition to ferritinophagy, autophagic degradation of lipid droplets through lipophagy is thought to contribute to ferroptosis by releasing fatty acids for fueling mitochondrial beta-oxidation. Indeed, blocking lipophagy prevented RSL3-induced lipid peroxidation and subsequent ferroptosis [376]. Key regulators of ferroptosis are also directly degraded by the lysosome. For example, GPX4 is degraded in response to erastin by chaperone-mediated autophagy, while transferrin receptors are partially delivered to the lysosome for degradation. Also, key signaling nodes involved in nutrient sensing and growth are implicated in this context, as induction of autophagy using mechanistic target of mTORC1 inhibitors sensitized cancer cells to ferroptosis and synergized with FINs to suppress tumor growth in animal models [377]. This demonstrates that ferroptosis is linked to conserved pathways of the ALP.

#### 3.5.2 Proteasome-associated degradation and ferroptosis

Proteasome function and ferroptosis show a reciprocal relationship in a context-dependent manner. Firstly, proteasomal degradation of key ferroptosis regulators such as GPX4, SLC7A11 and NRF2 sensitizes cells to ferroptosis insults [378], implying that proteasomal activity would promote ferroptosis. NRF2 is constitutively degraded by the UPS as it interacts with KEAP1, an adaptor subunit of the Cullin 3 E3 ubiquitin ligase (see chapter 3.2). Besides the canonical regulation of KEAP1 stability by electrophile stress, the autophagy receptor sequestosome 1/p62 also interacts with KEAP1. This interaction competes with NRF2 binding, especially in response to FINs, highlighting the interplay between the UPS and ALP systems. Polyubiquitination of additional anti-ferroptotic enzymes has been reported after induction of ferroptosis [379], yet the significance of this finding has not been further investigated so far. Also, deubiquitinylation has been implicated in ferroptosis. Specifically, OTUB1 has been shown to protect against ferroptosis by stabilizing the protein levels of SLC7A11 [380]. Another interesting UPS-related enzyme is BRCA1-associated protein 1 (BAP1), a deubiquitinase which targets histone 2A and thereby modulates the epigenetic regulation of SLC7A11 [381]. Secondly, next to the direct regulation of distinct key ferroptosis players, it has become apparent that the general global cellular proteasomal function is compromised during ferroptosis and sustaining proteasomal activity was shown to protect from ferroptosis [382]. Both chemical and genetic induction of ferroptosis by GPX4 depletion transiently reduce proteasomal activity [382], which promotes ferroptosis. As a compensatory mechanism for the insult to protein homeostasis, NFE2 Like BZIP Transcription Factor 1 (NFE2L1) is recruited. NFE2L1 regulates the adaptive component of proteasomal activity by targeting genes coding for proteasome subunits. However, the acute activation of NFE2L1 during ferroptosis occurs posttranslationally in a complex sequence of events. In most cells, NFE2L1 is virtually absent as it is quickly degraded. NFE2L1 undergoes deglycosylation by N-glycanase 1 and cleavage by DNA damage inducible 1 homolog 2 (DDI2). When proteasome function is compromised, NFE2L1 escapes proteasomal degradation and translocates into the nucleus, a process that is highly upregulated after induction of ferroptosis with RSL3 [379]. Multiple studies have shown that cells prone to undergo ferroptosis rely on this mechanism, whereas NFE2L1 deficient cells are more sensitive to ferroptosis [382]. Both, N-glycanase 1 [378] and DDI2 [379] are required for NFE2L1 to achieve its anti-ferroptotic capacity. Therefore, NFE2L1 seems to be a suitable candidate for targeting proteasomal activity to achieve higher susceptibility against inducers of ferroptosis.

In conclusion, degradation of cellular materials by lysosomes and proteasomes has profound impact on ferroptosis, both by means of global regulation of iron levels and protein quality as well as through a direct impact on key anti- or pro-ferroptosis guardians. More work is needed to put these findings into a physiologic framework and explore their potential for cancer therapy and beyond.

### 3.6 Chemical inducers

**Fig. 13:**
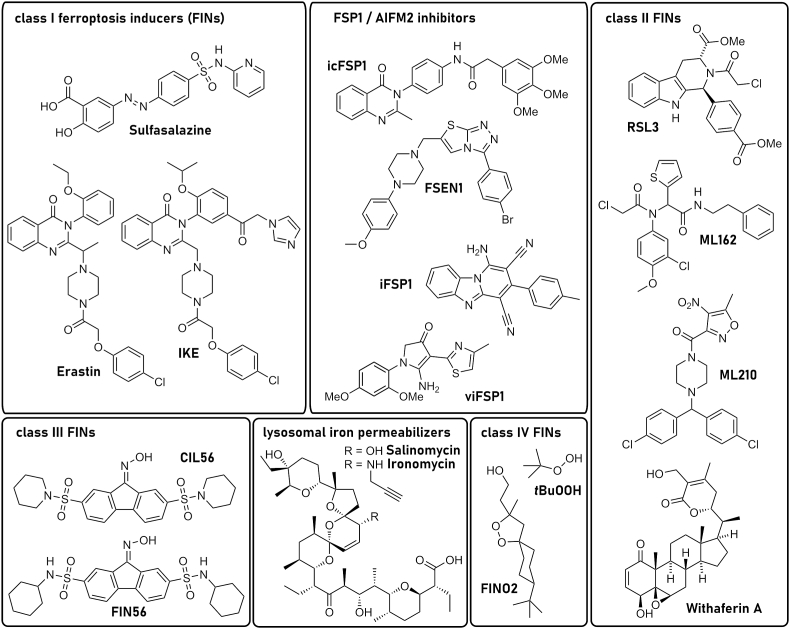
**Chemical structures of ferroptosis inducers**.

***Class I FINs*** (Fig. 13) include compounds that inhibit xCT, which imports cystine and exports glutamate in a 1:1 ratio. Blocking this antiporter causes GSH, for which cyst(e)ine serves as precursor, to be depleted. Reducing GSH levels increases the risk of entering a cascade of events resulting in the occurrence of lethal lipid peroxidation and ultimately cell death via ferroptosis. One way of blocking xCT function is to elevate the concentration of extracellular glutamate to millimolar concentrations, causing competitive inhibition of the antiporter [14]. In the case of ischemic stroke and neurodegenerative diseases, glutamate excitotoxicity (or a micromolar buildup of glutamate, the major excitatory neurotransmitter), is coupled to neuronal injury, with ferrostatin-1 mitigating this damage [5,383].

Erastin, initially identified in 2003 by Dolma et al. as a small molecule which could kill cancer cells possessing the RAS and small T oncogenes, induces oxidative, iron-dependent cell death by inhibiting xCT and subsequently depleting GSH [28]. However, the solubility, potency and metabolic stability compromise effectiveness *in vivo*. IKE, an inhibitor which acts through a reversible covalent mechanism of action, was designed as an improvement with a nanomolar (GI_50_ = 310 nM in HT-1080 cells) potency - a 200-fold increase compared to erastin (in BJeLR cells) [27]. Erastin also interacts with mitochondrial VDAC2 and 3 and has selective lethality in cells with activated RAS/rapidly accelerated fibrosarcoma/mitogen-activated protein kinase signaling [27].

Sorafenib, a multi-targeted kinase inhibitor with FDA approval for HCC with targets such as rapidly accelerated fibrosarcome, platelet-derived growth factor, and vascular endothelial growth factor [384], was suggested to impair xCT activity, although this was recently questioned by Zheng et al. [385]. In addition to being able to induce necrosis and possibly apoptosis, its mechanism of inducing tumor cell death was considered to be through the production of intracellular ROS in HCC [386]. Of note, since sorafenib induces a mixed mode of cell death, its lethality cannot generally be suppressed by any one type of cell death inhibitor, even though markers of ferroptosis are evident. Sorafenib also targets branched-chain amino acid aminotransferase 2 by impairing its transcription and reducing the pool of intracellular glutamate, which reduces the activity of the xCT transporter [387]. Another initiator of ferroptosis, sulfasalazine [388], which inhibits xCT in the high micromolar range, is an anti-inflammatory drug for rheumatoid arthritis and ulcerative colitis.

***Class II FINs*** (Fig. 13) include compounds that inactivate or degrade GPX4, which mediates the conversion of lipid hydroperoxides to lipid hydroxides utilizing GSH. RSL3, identified in a lethal screen against cells with oncogene RAS [26], possesses a chloroacetaldehyde electrophile group that is essential to inactivate GPX4 by binding directly with the selenocysteine within the active site through alkylation [28], as well as cysteine-66 [389]. ML162 also inactivates GPX4 through these two mechanisms [390,391]. Nevertheless, poor solubility and pharmokinetic properties precludes their use *in vivo*. ML210, unlike RSL3 and ML162, possesses a nitroisoxazole group that reveals a nitrile oxide group upon hydrolysis [392]. Some reports have suggested that these compounds have additional targets [393,394].

The natural product withaferin A was found to induce ferroptosis in high-risk neuroblastoma cells [395]. Remarkably, its activity is characterized by two modes: 1) At high concentrations, withaferin A reduces protein levels and directly abolishes the enzymatic activity by binding to GPX4, thus working as a class II ferroptosis inducer; and 2) at medium doses the natural product promotes ferroptosis by increasing the pool of labile Fe^2+^. The latter is accomplished through the inhibition of KEAP1 which leads to the NRF2-dependent upregulation of HO-1 [395]. The antioxidant enzyme HO-1 detoxifies heme into biliverdin which releases carbon monoxide and labile Fe^2+^.

***Class III FINs*** (Fig. 13) include FIN56, a more specific analog of CIL56, that works by depleting GPX4 protein abundance, but does not inhibit its transcription or enzyme activity. Additionally, FIN56 acts through the mevalonate pathway to deplete CoQ_10_ [50,51], which occurs by activating squalene synthase [396]. The active portion of FIN56 is its oxime functionality. Unlike FIN56, which exclusively induces ferroptosis, CIL56 can also induce an alternate cell death pathway that is separate from apoptosis, necroptosis, and ferroptosis [133,397].

***Class IV FINs*** (Fig. 13) react with free iron and induce lipid peroxidation via Fenton and Fenton-type reactions. FINO_2_ indirectly inhibits GPX4 and oxidizes ferrous iron to induce ferroptosis via its 1,2-dioxalane group (peroxide), which is required along with its hydroxyl moiety for its activity [398,399]. Tertiary-butyl hydroperoxide, has also been identified as a ferroptosis initiator as rescue is observed with ferrostatin-1, liproxstatin-1, alpha-tocopherol, and deferoxamine [400]. In this process, cardiolipin oxidation is essential for cell death induced by tertiary-butyl hydroperoxide and is reversible with cardiolipin inhibitors. Though cell-cell contacts provide cells with resistance to ferroptosis in human and murine cell lines, as well as reducing lipid peroxidation [401].

#### 3.6.1 FSP1 inhibitors

FSP1 is a relevant protein involved in safeguarding cells from ferroptotic cell death by regenerating the reduced form of CoQ_10_ [51], an RTA that protects cells from lipid peroxidation. Inhibition of FSP1 is an effective approach to sensitize certain cell types to ferroptosis. The first inhibitor described for FSP1 is iFSP1, which has been shown to target the CoQ_10_ binding pocket of the protein [51,101,102] (Fig. 13). However, iFSP1 acts specifically on human FSP1 [51,101]. To target the NAD(P)H binding pocket in the enzyme of both humans and rodents, a versatile inhibitor of FSP1 (viFSP1) was reported [101]. Another compound is FSEN1, which was described as an uncompetitive inhibitor that binds to a complex of FSP1 with its cofactor NADH, as well as its substrate and thereby inactivates the protein function [99]. In a biochemical assay that assessed the ability of purified FSP1 to reduce CoQ_10_, FSEN1 exhibited an IC_50_ of 313 nM while iFSP1 had an IC_50_ of 4 μM [99]. The latest generation of FSP1 inhibitors, represented by icFSP1, do not competitively or uncompetitively inhibit the enzymatic activity, but instead cause a relocalization and phase separation of the protein in cell culture and *in vivo*. IcFSP1 sensitizes cancer cells to ferroptosis and has a superior plasma stability compared to iFSP1, making it more suitable for xenograft studies in mice [100].

#### 3.6.2 Lysosomal iron permeabilizers

A natural product that induces ferroptosis by affecting the labile pool is salinomycin and its synthetic derivative ironomycin [168] (Fig. 13). These two chemical probes sequester the metal ions in the lysosomes which perturbs iron metabolism and leads to locally increased ROS formation. Salinomycin was shown to be an effective ferroptosis inducer in mesenchymal state cancer cells that have a high expression of the plasticity marker CD44 [168].

### 3.7 Chemical inhibitors

**Fig. 14:**
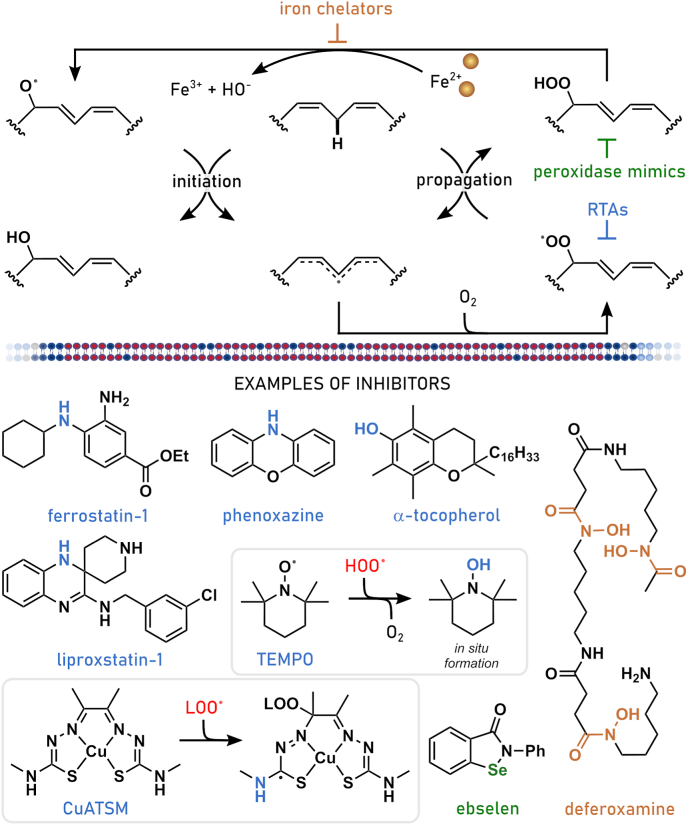
**Mode of action and structures of ferroptosis inhibitors**.

The key role of lipid peroxidation in ferroptosis execution is perhaps most clearly revealed by the wide variety of compounds that can inhibit this mode of cell death. As such, chemical inhibitors of ferroptosis have emerged as powerful tools, where they are routinely used to characterize cell death as occurring by ferroptosis, as well as to provide leads for therapeutics that target ferroptosis-associated diseases. The most effective point of intervention to date is in the lipid peroxidation chain reaction (see chapter 2.2.1), by reaction with the peroxyl radicals that propagate it (Fig. 14). RTAs generally react with peroxyl radicals by H-atom transfer (HAT) [402]. Perhaps the premier example is α-tocopherol, the most biologically active form of vitamin E, and a key endogenous ferroptosis-suppressing small molecule. Other prominent examples include ferrostatin-1 liproxstatin 1 and derivatives thereof [5,6], which were discovered by compound library screening in cell-based assays, and later shown to be RTAs [4]. However, also viral factors, in particular a viral insulin-like peptide referred to as vPIF-1, have been reported to function as powerful inhibitors of ferroptosis [403].

The HAT mechanism common to most RTAs enables ready rationalization and prediction of their activities; compounds that possess weak O-H or N-H bonds undergo fast HAT reactions with peroxyl radicals. Since the resultant O- or N-centered radicals are efficiently stabilized by an adjacent aromatic ring, most RTAs are aromatic alcohols (phenols) or aromatic amines. α-tocopherol is an example of a phenol. With an O-H bond dissociation energy of 77 kcal/mol, H-atom transfer from α-tocopherol to a peroxyl radical to form a hydroperoxide (which has an O-H bond dissociation energy of 87 kcal/mol) is highly thermodynamically favourable (i.e. Δ*G* = Δ*H* ∼10 kcal/mol). In comparison, the N-H bonds in ferrostatin-1 and liproxstatin 1 are stronger (∼83 kcal/mol), resulting in less favourable reactions [4]. As a result, α-tocopherol is inherently more reactive toward peroxyl radicals than ferrostatin-1 or liproxstatin-1. A second consideration is that the RTA-derived radicals arising from HAT must be persistent – they must not undergo reactions that produce radicals that can restart lipid peroxidation (*e.g.*, lipids or O_2_) – and instead react with a second peroxyl radical to form non-radical products. Despite being more inherently reactive as an RTA, α-tocopherol is significantly less potent than ferrostatin-1 and liproxstatin-1 in cells. While differences in cell permeability and/or subcellular localization may contribute, strong H-bonds between the reactive H-atom in α-tocopherol and the phosphodiester headgroups in PLs plays an important role, as it renders α-tocopherol unreactive to HAT [404]. Ferrostatin-1 and liproxstatin-1 engage in weaker H-bonds, such that there is more free RTA available to trap peroxyl radicals and inhibit lipid peroxidation. As such, although many RTAs can suppress ferroptosis, the most potent compounds identified to date are aromatic amines, since they have relatively weak N-H bonds and engage in relatively weak H-bonds with PLs. Based on these considerations, phenoxazines have emerged as attractive scaffolds for RTA-based ferroptosis inhibitor design [32]. Aromatic amines have the added benefit that they can be regenerated from the resultant aminyl radicals by reaction with hydroperoxyl radicals (HOO^•^, the conjugate acid of superoxide) – thereby trapping two ROS, and without being consumed in the process [405]. This reactivity appears to underlie the anti-ferroptotic reactivity of nitroxides, such as the persistent radical 2,2,6,6-tetramethylpiperidin-*N*-oxyl and related derivatives [279,406]. The anti-ferroptotic activity of CuATSM and related thiosemicarbazones has also been ascribed to RTA activity [407,408]. These compounds are examples of a handful of RTAs that do not react by rate-limiting HAT [409]. Instead, peroxyl radicals add to the ATSM ligand, which is held in conjugation by the metal center. Depending on the precise structure of the ligand, subsequent HAT or radical combination completes its RTA action. Since the reaction does not involve the metal centre, this activity is not unique to complexes of Cu, and can be observed with other metals, including Ni. This finding is particularly important because Cu toxicity has halted progression of CuATSM in clinical trials.

Since lipid peroxidation is auto-initiated (see chapter 2.2.1), the process can also be inhibited by reducing the rate of initiation by either reduction of the product lipid hydroperoxides – as does GPX4 at the expense of GSH – or sequestration of the labile iron that catalyzes their decomposition to initiating radicals. In the initial characterization of ferroptosis, this was demonstrated using ebselen, a chemical mimetic of the GSH peroxidases, and deferoxamine, an excellent iron chelator, respectively (Fig. 14). Since then, other metal chelators (*e.g*., ciclopirox, deferiprone) have been shown to be suppressors of ferroptosis. It is noteworthy that, to date, examples of these two classes of chemical inhibitor have been found to be far less potent than RTAs. Thus, there may be opportunities to significantly develop inhibitors in this area. Related to this point, necrostatin-1, an inhibitor of RIPK1 and necroptosis [410] and also an off-target inhibitor of ferroptosis [6,73], is proposed to react with lipid hydroperoxides to form a lipophilic sulfenic acid that can trap peroxyl radicals as an RTA [411].

Another strategy for ferroptosis suppression by small molecules involves treatment with less oxidizable lipids. Doing so effectively dilutes the highly oxidizable lipids, slowing both initiation and propagation of lipid peroxidation. A unique example of this strategy involves deuterated PUFA [110,412], wherein the H-atoms of the reactive bis-allylic moieties on oxidizable PUFAs have been replaced with heavier D-atoms, leading to a larger energetic barrier and consequently slower rate for propagation of lipid peroxidation. A similar protective effect has been observed upon treatment with monounsaturated fatty acids (*e.g*., oleic acid) [111], although the mechanism seems to be more complicated than simple dilution.

Lipid peroxidation can be initiated by superoxide or hydrogen peroxide produced from mitochondrial respiration or enzymes, such as NADPH oxidases and cytochrome P450 oxidoreductase, enzymes that lead to lipid peroxidation directly, such as lipoxygenases, or exogenous peroxides, such as *t*-butyl hydroperoxide [109,413]. However, a variety of inhibitors of these and other enzymes can have off-target RTA, iron chelation, or peroxidase activity, so the mechanism of protection must be carefully investigated in each case, such as using FENIX assays [404,411] and/or target knock-downs in cell assays. Structure-activity relationships can help illuminate such issues. Along these lines, it should be stressed that small molecules that specifically target enzymes that confer ferroptosis sensitivity remain widely lacking. One of few examples are the thiazolidinediones (alias glitazones). Although their conventional target is peroxisome proliferator-activated receptor (PPAR) γ, they have been shown to inhibit ACSL4, thereby causing remodeling of phospholipid bilayers from a PUFA-rich state to a MUFA/SFA-rich state in an PPARγ independent manner, thereby providing ferroptosis resistance – at least in cultured cells [115,116]. However, it is worth noting that PPARγ has been reported to modulate ferroptosis sensitivity [414]; thus, PPARs seem to also regulate ferroptosis in some contexts.

## 4 Feroptosis in diseases

### 4.1 Cancer

#### 4.1.1 Regulation of ferroptosis by oncogenic signaling

**Fig. 15:**
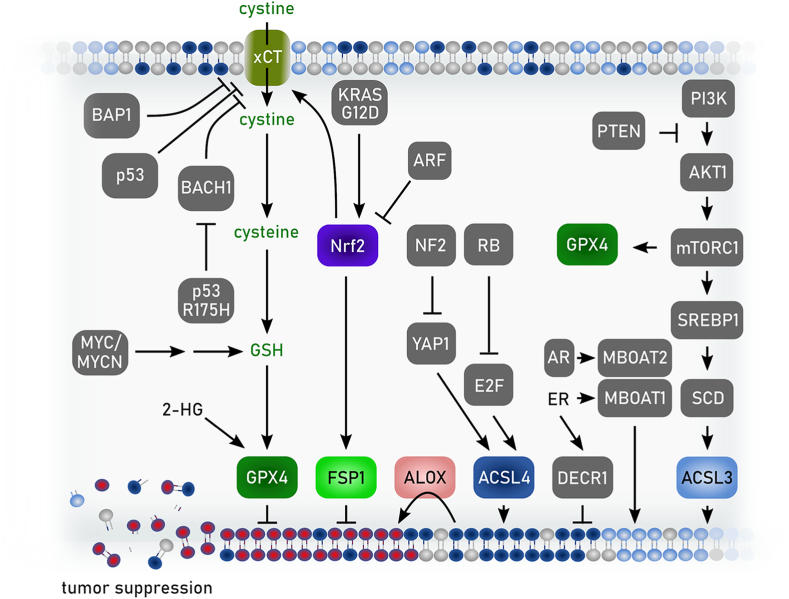
**Schematic overview of the complexity of different pathways modulating ferroptosis in cancer cells.** p53 and BAP1 suppress SLC7A11 to restrain the uptake of cystine. NRF2 and MYC/MYCN inhibit ferroptosis in both GPX4-or FSP1-dependent manner. KRAS G12D and ARF positively or negatively modulate NRF2 activities, respectively. NF2-YAP1 and RB-E2F axis regulate cell ferroptosis vulnerability by modulating ACSL4 expression and PUFA-PL content. Conversely, AR-MBOAT2, ER-MBOAT1, or PI3K-AKT-mTORC1-SERBP1-SCD pathways decrease cell sensitivity to ferroptosis by modulating MUFA-PL content. ACSL: acyl-CoA synthetase long-chain family member, AKT1: AKT serine/threonine kinase 1, ALOX: lipoxygenase, AR: androgen receptor, ARF: Tumor suppressor ARF, BAP1: BRCA1 associated protein 1, BACH1: BTB domain and CNC homolog 1, DECR1: 2,4-dienoyl-CoA reductase 1, E2F: E2F transcription factor 1, ER: estrogen receptor, FSP1: ferroptosis suppressor protein 1, GPX4: glutathione peroxidase 4, 2-HG: 2-hydroxyglutarate, KRAS: KRAS proto-oncogene, GTPase, MBOAT: O-acyltransferase domain containing, mTORC1: mammalian target of rapamycin complex 1, MYC: MYC proto-oncogene, MYCN: MYCN proto-oncogene, NF2: neurofibromin 2, NRF2: nuclear factor erythroid 2-related factor 2; PI3K: phosphatidylinositol 3-kinase; PTEN: phosphatase and tensin homolog, RB: RB transcriptional corepressor 1, SCD: stearoyl-CoA desaturase, SREBP1: sterol regulatory element-binding protein 1, xCT: SLC7A11 - solute carrier family 7 member 11, YAP1: Yes1 associated transcriptional regulator.

Dissection of the roles and mechanisms of ferroptosis reveals insights into tumor suppression and tumor immunity, which provides new therapeutic strategies for treating cancers by targeting the vulnerability of cancer cells [415]. Most importantly, ferroptosis has been shown to be a natural defense against cancer development; on the contrary, oncogene- or oncogenic signaling-mediated ferroptosis defense ability contributes to tumor progression, metastasis, and therapeutic resistance. Cancer cells with the signatures of high levels of reactive species and under nutritional deficit conditions expose vulnerabilities to ferroptosis, which is also under the control of cancer genetics in the manner of metabolism remodeling. Here, we summarize the current understanding of cancer genetics and ferroptosis, dissect the regulatory mechanisms of ferroptosis by genetic alterations, and explore strategies for targeting ferroptosis in cancer therapy (Fig. 15).

#### 4.1.2 Tumor suppressor and ferroptosis

Tumor suppressor p53 transcriptionally represses *SLC7A11* expression to restrain the uptake of cystine and sensitize cancer cells to ferroptosis in a 12-lipoxygenase-dependent manner [139,416]. The function of p53 in triggering ferroptosis explains why p53 still has tumor suppression capacity upon loss of function to induce cell cycle arrest, apoptosis, and senescence [417,418]. On the other hand, p53 hotspot mutations, which are found in over 50 % of tumors, have been proven not only to lose the ability to induce ferroptosis but also become more resistant to metabolic stress-induced ferroptosis, providing new insights to how mutant p53 promotes tumorigenesis and metastasis. For example, p53 R175H displays gain-of-function activities in the presence of BTB domain and CNC homolog 1 [419]. Indeed, several additional metabolic targets of p53 also contribute to its ferroptotic response and the defects in p53-mediated ferroptosis caused by an African-specific polymorphism in the *TP53* gene impair its tumor suppressor function [[420], [421], [422], [423]]. The other frequently mutated tumor suppressor, BAP1, which encodes a deubiquitinating enzyme, reduces histone H2A ubiquitination on the *SLC7A11* promoter region to repress *SLC7A11* expression and consequently promotes ferroptosis [381,424]. Mutation of BAP1 abrogates the tumor repressive ability and may contribute to the progression of uveal melanoma and renal cell carcinoma. Moreover, the dynamic regulation of ferroptosis is also implicated by the RB transcriptional corepressor 1/E2F transcription factor 1 axis [425,426].

Although gene alteration in *KEAP1* or *NFE2L2*, encoding NRF2, are found in approximately 4 % of all cancer samples according to the cBioportal database, tumors harboring KEAP1 or *NRF2* mutations are particularly higher in lung cancer (15 % or 9 %, respectively). Since KEAP1 negatively keeps the basal protein levels of NRF2 through the KEAP1-Cul3-RBX1 E3-ubiquitin ligase complex (see chapter 3.2), somatic mutations of KEAP1 abolish this protein-protein interaction with NRF2 and lead to NRF2 activation, which is targetable in lung cancer therapy. Upon activation, NRF2 transcriptionally stimulates cellular bioenergetics and enhances the intracellular antioxidant system, which is involved in GSH synthesis, iron metabolism, and NADPH metabolism. Somatic mutation-induced KEAP1 inactivation or NRF2 activation counteracts oxidative stress to protect cells from ferroptosis [195,308,427]. Moreover, the KEAP1/NRF2 axis may also regulate FSP1 expression and ferroptosis defense in a CoQ_10_-dependent manner [98]. Taken together, these studies suggest that negatively targeting NRF2-mediated metabolism remodeling is critical for tumor suppression.

Although tumor suppressors protect cells from tumorigenesis, their mutation increases the tumors’ resistance to high levels of ROS or survival under metabolic stresses. Fortunately, in certain contexts, mutations of tumor suppressor unexpectedly endow vulnerability to ferroptosis induction. It is reported that tumor suppressor cyclin dependent kinase inhibitor 2A (CDKN2A, also known as ARF) suppresses NRF2 activities and promotes ferroptosis during tumor suppression in a p53-independent manner [428]. In contrast, CDKN2A shows a high possibility of genomic deletion event in glioblastoma. CDKN2A deletion remodels the lipidome making tumors more susceptible to ferroptosis [429]. Tumors harbor genetically inactivated tumor suppressor neurofibromin 2, which primes tumor cells to ferroptosis induction through YAP-mediated upregulation of ACSL4 [136].

#### 4.1.3 Oncogene, oncogenic signaling, and ferroptosis

Mutations of the RAS proto-oncogene, especially the KRAS proto-oncogene, GTPase (KRAS) G12/13 mutation, have compromised GTP hydrolysis activity and thus keep a sustained activating status. KRAS activation stimulates downstream signaling cascades and drives tumor progression [430]. However, rewiring of cellular metabolism by KRAS exposes tumors to vulnerability upon ferroptosis induction [431]. Pancreatic cancer cells are addicted to glutamine and cystine metabolism, which is regulated by the KRAS/NRF2 axis [[432], [433], [434]]. Targeting glutamine metabolism by glutaminase inhibitors or cystine metabolism by cystine deprivation may present potentially useful clinical strategies [224,432]. It is noted that FSP1 is also upregulated in KRAS-mutant cells and is responsible for ferroptosis resistance and thus, combining FSP1 inhibitors with ferroptosis induction is proposed to be an efficient therapy of KRAS-mutant cancers [435]. Last but not least, KRAS mutated lung cancer cells also modulate ACSL3 expression to remodel fatty acid metabolism and to increase the content of MUFA-PLs [436]. Considering the recent discovery of selectively potent KRAS mutant inhibitors, it is worthy to investigate the combination of KRAS inhibitors with ferroptosis inducers in cancer therapy [437,438].

Besides the RAS signaling pathway, the PI3K signaling pathway is also well-defined as a hallmark of cancer, which participates in a broad range of cancer processes [439]. The oncogenic PI3K/phosphatase and tensin homolog deleted on chromosome 10/mTORC axis has been shown to be involved in ferroptosis resistance through promoting GPX4 expression and modulating stearoyl coenzyme A desaturase 1 expression to increase intracellular MUFA contents [119,377,440]. Another example is that cystine addiction of epidermal growth factor receptor mutated NSCLC renders these tumors particularly sensitive to cystine deprivation [441]. Last but not least, IDH1-mutated cancer cells, which have increased levels of the oncometabolite 2-hydroxyglutarate, also become vulnerable to GPX4 inhibition-induced ferroptosis [442].

Amplified *MYCN* oncogene is a well-known genetic alteration associated with poor prognosis in neuroblastoma. Surprisingly, *MYCN*-amplified neuroblastoma cells are particularly susceptible to ferroptosis [244]. This susceptibility is intricately entwined with perturbation in cysteine metabolism, redox equilibrium, and iron regulation within neuroblastoma cells, particularly in the context of elevated MYCN activity. The increased levels of the MYCN transcription factor disrupt the delicate balance of redox reactions by constraining intracellular cysteine availability, a crucial component for the synthesis of GSH, and by impeding the cystine/cysteine redox cycle [443]. Basal cysteine levels in neuroblastoma cells with amplified *MYCN* are relatively low, which is partly due to reduced cystine uptake via xCT and excessive usage of cysteine for protein synthesis [244]. Beside xCT, the SELENOP receptor, LRP8, is required to protect *MYCN*-amplified neuroblastoma cells from ferroptosis [253]. Deletion of *LRP8* in neuroblastoma cells leads to ferroptosis as a result of an insufficient supply of selenocysteine required for GPX4 biosynthesis (see chapter 2.2.4). It is still unclear if and how oncogenic MYCN or specific features of the cell of origin trigger ferroptosis sensitivity of neuroblastoma cells. Intriguingly, oncogenic MYCN in neuroblastoma cells also drives compensatory mechanisms to suppress ferroptosis, including induction of GSH synthesis (via *GCLC*) and an elevated usage of the transsulfuration pathway mediating methionine to cysteine conversion via CBS [244]. Nevertheless, elevated ROS production in fast-proliferating neuroblastoma cells with high MYCN poses a threat to these cells when cyst(e)ine and/or selenocysteine are limiting and ferroptosis-triggering mechanisms are not suffciently counteracted. Translation of these findings into new treatment concepts using ferroptosis induction in *MYCN*-amplified neuroblastomas, which is often a deadly disease as a result of multiple drug resistance mechanisms, is still missing due to a lack of compounds with pharmacological properties suitable for *in vivo* use in humans.

Sex hormone signaling regulates the global transcription and lipid remodeling involved in tumor development through prohibiting ferroptosis. DECR1-dependent PUFA remodeling enhances mitochondrial oxidative stress to promote lipid peroxidation and ferroptosis, which is a negatively regulated by androgen receptor, thus suggesting ferroptosis induction by targeting androgen receptor signaling in human prostate cancer [281,282]. Accordingly, ubiquitination of androgen receptor induced by a curcumin analog shows promising effects in suppressing temozolomide-resistant glioblastoma [444]. Recently, Liang et al. reported a differential regulation of MBOAT1 and MBOAT2 by estrogen and androgen receptor signaling, respectively [123]. This study thus provides unprecedented insights into the critical role of ferroptosis suppression related to sex hormone signaling and rationalizes inhibition of sex hormone signaling in tumor therapy.

#### 4.1.4 Pediatric cancers

Pediatric cancers represent a distinctive subset of malignancies in the context of tumor initiation, malignant progression and resistance development following currently used cancer treatment concepts. Unique characteristics of pediatric malignancies encompass several key facets, including lower mutational burdens, differences in cell of origin and specific oncogenic events, that collectively set them apart from adult tumors [445]. Tumor cells from pediatric malignancies also harbor specific vulnerabilities, which include a noticeable sensitivity to different cell death scenarios and may partly contribute to the more favourable overall survival rates as compared to those of adult cancers [446]. Remarkably, metabolic liabilities, including ferroptosis sensitivity, have been linked to specific pediatric cancer cell types.

A prominent disparity between pediatric and adult cancers lies in the considerably lower mutational burden observed in pediatric malignancies [447]. Unlike their adult counterparts, which often develop as a result of the gradual accumulation of genetic alterations over a longer lifespan, pediatric tumors originate in a relative short time from embryonic tissues that have undergone limited mutational changes [448]. This lower genetic complexity is a distinctive feature of pediatric cancers and may point towards dependencies linked to fewer oncogenic drivers. Specifically, the reduced mutational load suggests that pediatric cancers may have fewer mechanisms to defend against cell death pathways like ferroptosis, potentially making them more susceptible to this form of programmed cell death. Beyond mutational differences, the cell of origin plays a pivotal role in shaping the characteristics of pediatric cancers. Pediatric tumors typically originate from fast-proliferating embryonic or fetal tissues, in contrast to adult cancers that predominantly arise from mature tissues with few cycling cells [445]. This distinctive cellular origin can contribute to the unique vulnerabilities of pediatric cancers to specific therapeutic strategies. Understanding the developmental context of these tumors is essential for tailoring treatment approaches and optimizing outcomes.

Several studies on childhood neuroblastoma, the most common extracranial solid tumor arising during embryonal development from neural crest progenitor cells of the sympathetic nervous system [448,449], have demonstrated a special liability to ferroptosis induction [244,395,450,451], which exceeds the sensitivity to ferroptosis in most adult cancers.

Beyond neuroblastoma, other pediatric cancers display distinct insights into ferroptosis. In other neuronal and neuroectodermal pediatric cancers, susceptibility to ferroptosis is observed. Group 3 medulloblastoma tumors reveal a novel miRNA, miR-1253, disrupting iron homeostasis and presenting a strategy for targeting iron imbalance, while glioma cells may undergo ferroptosis upon exposure to the anesthetic agent sevoflurane, offering a potential therapeutic target [452]. Wilms tumor research has unveiled a group of long non-coding RNAs linked to ferroptosis, carrying both prognostic implications and roles in modulating tumor immunity [453]. In the context of hepatoblastoma studies, considerable attention has been directed towards investigating SLC7A11 and its epigenetic regulation as potential therapeutic targets [454]. In addition, renal medullary carcinoma displays a notable resistance to ferroptosis, a trait associated with specific transcription factors linked to loss of SWI/SNF related, matrix associated, actin dependent regulator of chromatin, subfamily B, member 1 [455,456].

#### 4.1.5 Ferroptosis in hematopoietic malignancies

Acute and chronic leukemias represents a diverse set of hematologic malignancies characterized by the abnormal proliferation and differentiation of developing leukocytes, prevalent across all age groups [457]. In particular myeloid malignancies originate from hematopoietic stem and progenitor cells and are often preceded by the pre-malignant stage of clonal hematopoiesis [458]. Clonal hematopoiesis is increasing with age and caused by single mutations in hematopoietic stem and progenitor cells such as in DNA methyltransferase 3 or Tet methylcytosine dioxygenase 2 leading to expansion of mutant clones over time. Strikingly, clonal hematopoiesis is not only associated with a moderately increased risk of leukemic transformation, but also with an increased incidence of cardio-vascular disease caused by mutated myeloid effector cells. Additional genetic and epigenetic abnormalities then lead to the reprogramming and distortion of differentiation with the generation of leukemic stem cells and high numbers of proliferating leukemic blasts [459]. Healthy hematopoietic stem cells are also vulnerable to genotoxic or other stress-induced insults, such as oxidative stress, which can impair their function over time, promoting pre-malignant states that may eventually drive blood cancers. Zhao and colleagues demonstrated that human hematopoietic stem cells, as opposed to progenitors, are susceptible to ferroptosis due to the loss of the histone deubiquitinase Myb like, SWIRM and MPN domains 1 [460], leading to their selective loss. Their study suggests that decreased protein synthesis in hematopoietic stem cells leads to increased cell death, which can be fully rescued by ferroptosis inhibitors. Hematological malignancies encompass a spectrum of diseases, including acute myeloid leukemia (AML), chronic lymphocytic leukemia, chronic myeloid leukemia, multiple myeloma, and acute lymphoblastic leukemia, making disease specific therapies challenging [461]. The current therapeutic strategies include combination chemotherapies to target proliferating leukemic cells and allogeneic stem cell transplantation. However, despite many patients entering complete remission, almost all relapse. In AML for example, the current management heavily relies on intensive chemotherapy, notably utilizing the combination of cytarabine/daunorubicin [462]. More recently, advanced targeted treatment strategies, such as combining the B cell lymphoma-2 inhibitor Venetoclax with the hypomethylation agent Azacytidine, which can also target leukemia stem cells, have been employed [463]. Despite these recent advances in treating AML, 5-year survival rates for individuals older than 60 years are as low as 10 % due to the development of primary and secondary resistance and subsequent relapse, which is clinically challenging. Therefore, exploring new anti-tumor strategies is of vital importance and targeting ferroptosis with several pharmacologically amenbale nodes might present a path forward in treating certain hematological malignancies. Pontel and colleagues demonstrated that in T and B cell acute lymphoblastic leukemia, the FSP1 promoter is hypermethylated, resulting in the silencing of FSP1 expression. This genetic alteration creates a specific reliance on GSH/GPX4-centered anti-ferroptosis defenses [464], thus presenting an opportunity to disrupt this anti-ferroptosis axis. Moreover, leukemic cells, unlike normal myeloid cells, have been shown to depend on the aldehyde dehydrogenase 3a2 enzyme. This enzyme oxidizes long-chain aliphatic aldehydes and thus prevents cellular oxidative damage and ferroptosis [465]. Sabatier et al. have demonstrated that CCAAT-enhancer binding protein α, a crucial factor in both normal and leukemic differentiation, plays a key role in maintaining lipid homeostasis and redox balance [466]. This study revealed a previously unknown vulnerability of fms-related receptor tyrosine kinase 3-mutant AML cells to ferroptosis, suggesting potential therapeutic opportunities through the use of ferroptosis-inducing compounds. Recently Garciaz and colleagues demonstrated that ironomycin induces mitochondrial stress through iron depletion in AML cells [467]. The iron-dependent cell death induced by ironomycin is characterized by significant lipid peroxidation and concurrent activation of Bcl-2-associated X protein/Bcl-2 homologous killer. These effects were not rescued by classical inhibitors of ferroptotic or apoptotic pathways. Notably, they found that the combination of low doses of Venetoclax and ironomycin was well tolerated in mice, causing no systemic toxicity. This combination resulted in a marked anticancer effect, leading to significantly increased survival rates in preclinical models. Interestingly, Kalkavan and colleagues reported that persister cells, which evade apoptosis during targeted cancer therapies involving pro-apoptotic BH3 mimetic compounds, exhibited increased sensitivity to ferroptosis upon GPX4 inhibition [468]. Although, this observation was made in lung cancer cell lines, the possibility remains that this approach could also be applicable to other cancer types, such as leukemia, after exposure to Venetoclax.

#### 4.1.6 Ferroptosis in lung cancer

Lung cancer is the leading cause of cancer-related deaths worldwide [469]. In 2020, lung cancer was the second most diagnosed cancer entity after breast cancer and the leading cause of malignant cancer-related death in males [470]. Lung cancer can be subdivided into NSCLC and small cell lung cancer (SCLC), which account for 85 % and 15 % of all cases, respectively [471]. Lung cells are specialized in processing oxygen and within lungs, oxygen handling is further compartmentalized through alveoli at the end of bronchi, which are essential for respiration. Hence, lung cells specifically require a sensitive network sensing cellular oxygen load. Given this compartment-specific function, it is not surprising that normal and malignant lung cells require a tightly regulated antioxidant defense system. The fact that ferroptosis ensues as a result of catastrophic collapse of lipid peroxide-specific detoxification has raised an interest in therapeutic induction of ferroptosis in cancers derived from the lung. Pulmonary neuroendocrine cells - the proposed cells-of-origin for SCLC - are responsible for sensing oxygen and subsequently regulating its uptake [472]. This inert function in regulating oxygen uptake could indicate a high dependence on antioxidant defense systems and SCLC deriving from this particular cell type might be particularly vulnerable to ferroptosis. Indeed, non-neuroendocrine SCLC cells are characterized by a ferroptosis-prone lipidome and therefore sensitive to the induction of ferroptosis, whereas neuroendocrine SCLC cells are ferroptosis resistant but specifically sensitive to the inhibition of the Trx system. Combined induction of ferroptosis and inhibition of the Trx system successfully reduced tumor growth [473].

Similarly, NSCLC has been described in several studies to be vulnerable to the induction of ferroptosis. Targeting GPX4, SLC7A11 or FSP1, which have all been reported to be upregulated in NSCLC, has been shown to reduce tumor growth in different NSCLC model systems [28,98,474]. Whereas low expression of ACSL4 has been described to promote NSCLC invasiveness [475], its increased expression correlates with increased ferroptosis sensitivity in NSCLC [476,477]. Furthermore, NSCLC cell lines with acquired resistance to targeted therapy or radiation therapy have been shown to be resensitized to the treatment by increasing ferroptosis sensitivity [478,479]. Moreover, depletion of nitrogen-fixing bacteria S-like protein, a cysteine desulfurase harvesting sulfur from cysteine essential for biosynthesis of iron-sulfur cluster, sensitizes lung adenocarcinoma to ferroptosis [228].

In the context of NSCLC, KRAS is the most frequently mutated driver oncogene [480]. Interestingly, ferroptosis has firstly been described to cause synthetic lethality in RAS mutated cancer cells [[25], [26], [27]]. Yet, more recent literature using isogenic cellular model systems suggests that KRAS-mutated cancer cells are in fact more resistant to ferroptosis. Along these lines, Bartolacci et al. describe that blocking fatty acid synthase and the downstream Lands cycle and thereby synthesis of SFA and MUFA species specifically sensitizes KRAS mutant lung cancer [122]. Moreover, expression of mutant KRAS was shown to upregulate FSP1 in an NRF2-dependent manner, which in turn leads to increased ferroptosis resistance and increased tumor initiating capacity [435].

#### 4.1.7 Ferroptosis in liver cancer

Liver tissue and the element iron are known to have an intrinsically close relationship. The liver is the primary site for iron storage and in case of systemic iron overload, liver cells are particularly vulnerable to transition into liver cancer cells. Moreover, even in absence of systemic iron overload, iron-enriched liver cancer cells are considered more aggressive compared to their iron low counterparts [481]. Thus, triggering ferroptosis in these liver cancer cells might be a promising therapeutic option.

In liver cancer research, interest in ferroptosis has been sparked when it was reported that sorafenib - a clinically established multi-kinase inhibitor used to treat HCC - induces ferroptosis in liver cancer cells. However, an opposing study by Zheng et al. demonstrated that sorafenib is not a classical ferroptosis inducer, but induces a form of nonspecified cell death [385,482], even though markers of ferroptosis are induced by sorafenib [29]. In turn, canonical ferroptosis inducers, such as l-buthionine sulfoximine as the bona fide inhibitor of GCLC and auranofin as an inhibitor of TrxR were demonstrated to have antitumor activity on liver cancer cells both *in vitro* and *in vivo* [427]. The combination of the established ferroptosis inducer RSL3 and sorafenib showed beneficial synergistic effects in liver cancer and bypassed the therapeutic limitations in chemotherapy-resistant liver cancer [427]. Apart from these promising *in vivo* data, the induction of ferroptosis in liver cancer cells accompanied with an increase in lipid peroxidation may also have adverse effects, as ferroptosis has also been shown to promote liver degeneration and fibrosis, which in turn is a risk factor for the development of HCC [483,484].

Notably, ferroptosis inhibition was investigated as an anti-tumor strategy even before the nomenclature of ferroptosis was established. In 1992, Hie-Won et al. demonstrated that administration of the ferroptosis inhibitor deferoxamine reduced tumor growth in HCC-bearing mice [485].

In addition to animal-based research, Gao et al. developed a clinical model by correlating the outcome of patients with HCC to the expression of ferroptosis-associated genes in tumor tissue. This is aimed at predicting both overall survival and response to immunomodulating therapy by screening for ferroptosis-related genes in HCC [486].

#### 4.1.8 Cancer treatment by low-temperature plasma

Plasma is the 4^th^ physical state of matter, characterized an ionized gaseous mixture, holding higher energy than solid, liquid and gas phases, and consisting of a wide array of components such as electrons, ions, radicals and ultraviolet ray. Representative natural plasmas include the sun, aurora, and thunder. Recent progress in electronics discovered body-temperature plasma, designated as low-temperature plasma (LTP), non-thermal plasma or cold plasma since the 1990's [487]. LTP has drawn the attention of researchers for possible medical applications, and is used for disinfection, cancer therapy, coagulation, wound healing, immunomodulation, drug delivery and facial rejuvenation mostly at the preclinical stage [488].

The core biological effect of LTP is loading oxidative stress to the target area by the application of electrons to atmospheric molecules [489,490]. Both direct and indirect exposures are possible, and one of the standards of the latter is plasma-activated lactate (PAL) [491], containing H_2_O_2_, NO_2_^−^ and other identified chemical species such as glyoxylate and 2,3-dimethyltartrate [492]. Cancer cells are characterized by persistent self-replication, destruction of normal structures and high cytosolic catalytic Fe^2+^ [493]. Indeed, excess iron is a risk for carcinogenesis, where mutagenic environments enabled by the Fenton reaction can eventually cause ferroptosis-resistance [494,495]. Iron is essential for the *de novo* production of DNA as a co-factor of ribonucleotide reductase [496]. Therefore, both direct exposure of LTP and PAL can promote higher oxidative damage to cancer cells compared to non-cancerous cells, which has been reported in a variety of cancer types. The cell death mode is either apoptosis or ferroptosis, depending on the cancer type [497]. As an example, NO-dependent oxidative lysosomal damage leads to ferroptosis through autophagic process in mesothelioma cells exposed to PAL [498]. Thus, LTP and PAL are expected to be applied as an additional cancer therapy to the guideline protocols, especially for use in somatic cavities and surgical margins. Currently, several clinical trials are run with the LTP system, including the treatment for cervical intraepithelial neoplasia, recurrent nasopharyngeal carcinoma, surgical margin, rosacea, wound treatment, dental restoration, pterygium and lacrimal gland obstruction (https://clinicaltrials.gov).

In summary, targeting different types of cancer through induction of ferroptosis is a promising therapeutic strategy, and several efforts are currently underway to develop selective GPX4, SLC7A11 and FSP1 inhibitors with enhanced bioavailability. For now, approved anti-cancer drugs with ferroptosis inducing capacities may pave the way for the concept of therapeutic ferroptosis induction in leukemia, as well as pediatric, liver and lung cancer. Additional preclinical studies in cell and mouse models are imperative to enhance our understanding of ferroptosis preventing pathways. Since the discovery of ferroptosis, many investigations have focused on triggering ferroptosis in cancer cells. However, inhibition of ferroptosis by iron chelators represents a potential therapeutic approach as well. As pointed out above, the potential use of ferroptosis as a therapeutic strategy should take into account that inhibition of ferroptosis by administration of iron chelators also mediates antitumor effects and thus represents a potential therapeutic approach. It is yet to be seen which therapeutic approach will prevail for clinical application.

### 4.2 Tissue ischemia/reperfusion

Ischemia/reperfusion (I/R) injury (IRI) is a complex and pervasive condition that occurs when the blood flow to a tissue is interrupted for a certain amount of time (ischemia) and subsequently restored (reperfusion). IRI affects a wide range of organs such as liver, kidney, lung, heart, brain, as well as intestines and occurs frequently upon various disorders notably stroke and myocardial infarction, during solid organ transplantation and surgical interventions. I/R of the entire organism is clinically observed upon resuscitation upon cardiac arrest. Although reperfusion of ischemic tissue is essential for its survival, concomitant reoxygenation may initiate a cascade of damaging events through the generation of ROS, cell death, inflammatory responses as well as possible secondary cell death routines. The formation of mitochondrial ROS upon reperfusion is considered an early event, presumably initiated by succinate accumulation during ischemia, which is rapidly re-oxidized upon reperfusion by succinate dehydrogenase, thereby generating a burst of superoxide by reverse electron transport at mitochondrial complex I [499]. This deleterious event is associated with the generation of superoxide and concomitant production of H_2_O_2_ [499]. Whereas Fenton chemistry give rise to the highly reactive hydroxyl radical, protonation of superoxide yields hydroperoxyl radical, both of which are capable of initiating lipid peroxidation and autooxidation of biological membranes [2]. Recent evidence suggests, that ferroptosis, a pathologically relevant and regulated form of necrotic cell death, driven by excessive iron-dependent lipid peroxidation, is a subsequent potential prime event and key contributor to this multifaceted disease [500]. Most of what is known today about the role of ferroptosis is derived from studies using ferroptosis inhibitors such as the RTAs liproxstatin-1 and ferrostatin-1, which have been shown to protect from IRI in a wide range of tissue contexts [6,34,188,[501], [502], [503], [504], [505]].

#### 4.2.1 Liver

Upon its discovery, liproxstatin-1 was first demonstrated to ameliorate tissue demise inflicted by hepatic IRI, as exemplified by a reduction in necrotic tissue areas and the serum liver damage markers alanine aminotransferase/aspartate transferase [6]. Besides chemically synthesized RTA's, endogenous substances exist that also possess substantial RTA activity, for example the archetype natural RTA, α-tocopherol, as well as the reduced forms of CoQ_10_ and vitamin K. Both, α-tocopherol as well as the vitamin K analog MK-4 have shown protective effects in liver IRI models, by a decrease in necrotic areas and alanine aminotransferase/aspartate transferase values. Further, it was demonstrated that MK-4 as well as α-tocopherol treatment resulted in a reduction in I/R-induced lipid peroxidation, as measured by 4-HNE staining [52,504], as well as a decrease in immune cell infiltration [52]. Increased *Ptgs2* mRNA levels have been previously associated with cell death by ferroptosis *in vitro* [28] and indeed attenuated *Ptgs2* levels have been found in treated livers, although the underlying mechanisms are still unclear [504]. Moreover, pretreatment with deferoxamine, an iron chelator and ferroptosis inhibitor, significantly ameliorated liver IRI [504]. Of note, a ferroptosis independent study indicated that necrotic or regulated necrotic cell death routines and not apoptosis primarily drive cell death upon liver tissue upon IRI, as proven by the presence of terminal deoxynucleotidyl transferase-mediated dUTP nick-end labeling (TUNEL) positivity, absence of active caspase-3 staining and ineffective treatment with caspase inhibitors [506].

#### 4.2.2 Kidney

Akin to the effects seen with liproxstatin-1 in the liver, ferrostatin-1 and its improved analog have been shown to protect from kidney IRI by decreasing kidney tubule injury scores and serum urea and creatinine values [34]; similar observations have later been reported with MK-4 [52]. Furthermore, genetic evidence for the contribution of ferroptosis to kidney damage upon I/R has been provided using mice with a targeted active site selenocysteine to cysteine mutation within the key ferroptosis regulator GPX4 U46C. Since the presence of GPX4 U46C renders cells inherently sensitive to peroxide-induced ferroptosis, *Gpx4 U46C* animals showed a significantly shorter survival time after renal IRI compared to wildtype animals, accompanied by increases in serum creatinine and urea values, tubular damage scores and 4-HNE positivity [73]. Likewise, mice deficient in the gene encoding FSP1, also showed a worse outcome upon kidney I/R [73], indicating that ferroptosis suppressing systems play a contributing role in containing I/R-related tissue damage.

#### 4.2.3 Lung

The lung is the solid organ with the worst clinical outcome and the lowest survival rate after transplantation. Characteristics of ferroptosis have been shown to emerge at early phases of reperfusion in both human and mouse transplanted samples. In human lung tissue, ferroptosis-related signaling pathways leading to iron accumulation and lipid peroxidation were found to be activated. Treatment with liproxstatin-1 during cold ischemia significantly ameliorated IRI by alleviating tissue injury and the inflammatory response triggered after I/R in mice [505]. Shortly after reperfusion of the clamped left lung in rats, ACSL4 and PTGS2 were highly upregulated and GPX4, FTH as well as SLC7A11 were observed to be significantly downregulated. It was further suggested, that ferroptosis driven by I/R may trigger inflammatory responses since pro-inflammatory cytokines, such as TNF-α, IL-1β and IL-6, were found to be elevated after signs of ferroptotoic cell death were noticed [507]. Most importantly, ferrptosis-induction after immune cell infiltration shortly after reperfusion is a key event that is gaining increasing importance. In the chronic obstructive pulmonary disease, pro-inflammatory macrophages recruited to the lung upon cigarette smoke exposure were found to increase ACLS4 expression in alveolar typeII epithelial cells leading to increased lipideperoxidation and cell death resulting in emphysema [508]. Thus, macrophages known to accumulate in the lung after IRI might play a similar role inducing further damage to the lung and resulting in primary graft dysfunction and long-term to lung transplant rejection.

#### 4.2.4 Heart

In the context of IRI in the heart, treatment of transplanted mice with ferrostatin-1 resulted in a marked decrease in cell death, neutrophil infiltration as well as the oxidized PE phospholipid marker HOO-C20:4/C18:0-PE. Immunostaining against active caspase-3 was negligible in transplanted hearts, suggesting alternative modes of cell death [501]. Additionally, using an *in vivo* model of myocardial IRI, ferrostatin-1 was able to reduce the infarct area as well as neutrophil recruitment. Moreover, a long-term analysis after myocardial infarction revealed that a smaller infarct size was correlated with reduced fibrotic events in ferrostatin-1 treated mice [188,501], demonstrating that early intervention can ameliorate secondary complications. In addition, an increase in non heme iron as well as increased *Ptgs2*, *FTL* and *FTH* mRNA levels were shown upon cardiac IRI, indicating that ferroptosis may contribute to the extent of cardiac IRI [188]. Accordingly, treatment of isolated hearts with the iron-chelator deferoxamine provided beneficial effects [184].

#### 4.2.5 Brain

In the transient mouse middle cerebral artery occlusion (MCAO) model, liproxstatin-1 treatment commencing at the time of early reperfusion markedly attenuated MCAO-induced functional deficits, improved the neuroscore and reduced infarct volumes, suggesting a direct involvement of ferroptosis in ischemic stroke [502]. To further explore the translational value of ferroptosis inhibitors, MCAO mice were treated 6 h after reperfusion and indeed delayed liproxstatin-1 treatment still significantly prevented ongoing neuronal damage and reduced infarct volume, albeit to a lesser degree, stressing the benefit of early intervention [502].

#### 4.2.6 Intestines

Interestingly, by using a mouse model of intestinal IRI, it was shown that protein levels of GPX4 were decreased at early timepoints after reperfusion, whereas the expression of PTGS2 was increased and treatment with liproxstatin-1 attenuated the decrease in GPX4 expression and reduced cyclooxygenase-2 expression. Furthermore, liproxstatin-1 treatment prevented lipid peroxidation and reduced serum lactate dehydrogenase, TNF-α, and IL-6 levels. In addition, remote organs such as the lung and liver, were protected from the consequences of intestinal I/R upon liproxstatin-1 treatment as well [503].

The studies described above provide several lines of evidence that ferroptosis is a potential disease-relevant mechanism contributing to I/R-related injuries across various organ settings. Therefore, the development of new treatment paradigms based on ferroptosis inhibition holds great promise to be implemented as new therapeutic option for the management of these conditions.

### 4.3 Inflammation

Recent studies suggest that ferroptosis plays an important role in the development of chronic inflammatory diseases, such as non alcoholic steatohepatitis, chronic kidney disease, inflammation-induced neurodegeneration and cancer (see additional chapters in 4). However, how much of these effects can be traced back to a direct involvement of ferroptosis in the regulation of immune responses remains unclear. Early reports started to investigate the function of ferroptosis in T cells by targeted deletion of GPX4 [86]. While thymic development of T cells deficient in GPX4 remained unaffected, the maintenance and activation of T cells in the periphery was impaired, which resulted in defective immunity against both, viral and parasitic infections [86]. Furthermore, rapid ferroptotic cell death in T cells in the absence of GPX4 could be prevented by delivery of α-tocopherol or dietary vitamin E, which also restored protective anti-viral immunity [86]. Notably, in contrast to the activation of effector CD4^+^ and CD8^+^ T cells, deleting GPX4 in memory T cells impaired the functionality of memory CD4^+^ but not CD8^+^ T cells [86,509]. This suggests that immune cells may have different requirements for buffering lipid peroxidation and ferroptosis. In fact, GPX4 does not appear to be required by all immune cells, as its expression is required for the prevention of lipid peroxidation and ferroptosis in B1 and marginal zone B cells, but not in follicular B2 cells [89].

Importantly, the need for buffering lipid peroxidation and ferroptotic cell death could be essential to promote chronic inflammation, as GPX4 expression is increased in patients suffering from multiples sclerosis [510]. This is in line with a recent study showing that ILC2 mediating chronic airway inflammation rely on potent cell intrinsic lipid buffering systems to prevent lipid peroxidation. Pharmacological inhibition or genetic deletion of the enzyme converting free fatty acids in neutral lipids, diacylglycerol O-acyltransferase 1, ameliorated allergen-driven airway inflammation [511]. Although, the direct effect of ferroptosis was not assessed in this study, the observed increase in lipid peroxidation suggests that targeting anti-ferroptotic pathways could be a promising general approach for the treatment of chronic inflammation. However, while the study of immune cells supports a view that exploiting ferroptotic vulnerabilities could be beneficial, the induction of ferroptosis in the tissue may actually promote chronic inflammation, as recent studies suggest that ferroptosis can also contribute to development and exacerbation of chronic inflammatory diseases.

Such a pro-inflammatory role can be attributed in parts to the release of DAMPs upon ferroptotic cell death, including HMGB1 [512], ATP [366] and lipid oxidation products (e.g. oxidized PL or 4-HNE). These molecules can trigger signaling of pattern recognition receptors expressed by myeloid immune cells such as monocytes, macrophages and DCs, and thereby promote their pro-inflammatory activity. Although DAMP release upon ferroptotic cell death may contribute to the clearance of dying cells, insufficient resolution of DAMPs and other ferroptosis-associated mediators may contribute to development of chronic inflammation and immunopathology. Notably, ferroptotic cell death in a pancreatic *Gpx4* conditional knockout mouse model further promotes tissue injury and inflammation in experimental pancreatitis [513]. Moreover, ferroptosis exacerbates tumor progression in a KRAS-driven pancreatic cancer mouse model via the recruitment and activation of macrophages through a TMEM173/Stimulator of IFN response CGAMP interactor 1-dependent DNA sensor pathway. Consequently, immuno-stimulatory DAMP-release during ferroptotic cell death may further fuel ongoing inflammation and promote progression of inflammatory diseases.

Nevertheless, there is still ongoing debate on the immunoregulatory properties of ferroptosis, as recent evidence for potential anti-inflammatory characteristics of ferroptosis emerge. Despite the ferroptosis-associated release of inflammatory DAMPs and cytokines including C-X-C motif chemokine ligand 1, TNF and IFN-β, ferroptosis has been identified as rather less immunogenic cell death modality compared to other forms of cell death including necroptosis and immunogenic apoptosis [364]. Consequently, ferroptotic cells show weak potential to stimulate DC-mediated CD8^+^ T cell immune responses and fail to generate protective anti-cancer immunity in a vaccination-based approach. This may in part reflect the idea that the immunoregulatory quality of DAMPs released by ferroptotic cells seem to substantially differ from immunogenic forms of cell death. Accordingly, several studies indicate that ferroptotic cell death may selectively result in the release of immune-inhibitory molecules that may negatively regulate inflammation induced by stimulatory DAMPs. In this context, upregulated expression of PTGS2, a key enzyme required to produce the bioactive lipid prostaglandin E2 (PGE_2_), has been noted as a feature of ferroptosis [28]. PGE_2_ is an immunosuppressive factor in cancer and promotes a dysfunctional program in conventional type-1 DCs [514] and NK cells [515], suggesting that PGE_2_ release during ferroptosis might counteract immune cell activation via stimulatory DAMPs. The consequences of PGE_2_ production by ferroptotic cells for disease progression may be very distinct depending on the underlying disease context and cell type affected. While a release of immune-suppressive PGE_2_ might be advantageous for the resolution of inflammation in tissue injury, on the contrary, ferroptosis-related PGE_2_ production in cancer could possibly shut down effective conventional type-1 DCs-mediated anti-cancer immune responses. Interestingly, spontaneous ferroptotic cell death has been proposed as a mechanism by which intratumoral neutrophils [516] exhibit their immune-suppressive function in cancer, which was at least in part mediated by the release of PGE_2_. This suggests a sequential link between ferroptosis and inhibition of immune cell function in cancer and indicates that depending on the disease context, patients might benefit from blockade of PGE_2_ signaling in ferroptotic cells.

Besides PGE_2_, the release of oxidized lipids itself is a prominent factor which negatively impacts on immune cell activation. The accumulation of lipid bodies containing oxidatively truncated lipids has been shown to impair the ability of dendritic cells to cross-present antigens to CD8^+^ T cells [365]. Moreover, CD36-mediated uptake of fatty acids in CD8^+^ T cells reduces their ability to produce pro-inflammatory cytokines including IFN-γ and TNF-α, resulting in reduced anti-tumor effector functions [517]. Therefore, an abundance of oxidized lipids in ferroptotic tissue may additionally exhibit anti-inflammatory properties.

The lipid-rich central nervous system (CNS) is particularly susceptible to radical-mediated lipid peroxidation; thus, neuroinflammation needs to be tightly regulated to prevent ferroptosis. In various neurological and psychiatric disorders CNS infiltration of peripheral immune cells, including T and B cells, and activation of brain-resident microglia [518] can be found and contribute to neuronal stress and damage [519]. A prime example of a neuroinflammatory condition is multiple sclerosis, characterized by inflammatory lesions that harm the CNS, leading to neurodegeneration and clinical impairment [520]. Recent studies point to a prominent role of ferroptosis in neuronal injury during neuroinflammation. Increased iron deposits and oxidized PLs are observed in multiple sclerosis brains [33] and spinal cords [521,522] of experimental autoimmune encephalomyelitis (EAE) mice, the animal model of multiple sclerosis. Moreover, decreased GPX4 and increased ACSL4 levels were noted in neurons of multiple sclerosis brains and spinal cord of EAE mice [358,359,521].

Mechanistically, ferroptosis can be induced by neuroinflammation [523], but at the same time can also foster neuroinflammation [358]. Treating EAE mice with ferroptosis inhibitors halts the initiation of T cell activation and subsequent disease symptoms. This suggests the sequential relationship between upstream ferroptosis activation and downstream T cell activation. Moreover, supernatant from neurons in which ferroptosis was induced results in the activation of T cells with elevated release of IL-2 and IFN-γ [358]. Notably, a viral-mediated knockdown of *Acsl4* in neurons resulted in a dampening of ferroptosis and T cell activation in EAE with subsequent alleviation of disease symptoms [358].

With established neuroinflammation, microglia substantially contribute to ferroptosis induction, as they exhibit a distinct ferroptosis-associated transcriptomic signature in response to iron dysregulation [523]. The absence of microglia reduces neuronal lipid peroxidation and considerably delays neuronal death during ferroptosis *in vitro*. Besides ACSL4, SEC24 homolog B, coat protein complex II component (SEC24B) is involved in regulating microglial ferroptosis [523]. SEC24B manages cargo packaging from the ER to the Golgi apparatus, impacting iron homeostasis in microglia by controlling the labile iron pool. The absence of SEC24B in microglia provides robust protection partly through reduced iron exposure. By contrast, oligodendrocytes provide a line of defense against neuronal ferroptosis by secreting FTH [524]. Oligodendrocytes exhibit high expression of FTH and employ an unconventional secretion pathway that involves extracellular vesicles for its release. Impairing the release of extracellular vesicles or curtailing the expression of FTH in oligodendrocytes results in neural loss and oxidative injury in mice [524].

Consequently, the use of ferroptosis inhibitors not only dampens T cell activation, but also slows disease progression in preclinical models of relapsing-remitting and progressive multiple sclerosis, underscoring ferroptosis as a modifiable and detrimental factor in chronic neuroinflammation [33,522]. However, apart from ongoing developments of mainly RTA-based anti-ferroptotic agents [39], targeting the upstream epigenetic regulation of ferroptosis offers an alternative therapeutic avenue for various pathologies. Of note, neuroinflammation leads to the induction of the histone methyltransferase G9 in neurons [521]. The abundance of the G9a-catalyzed repressive epigenetic mark H3K9me2 in neurons of EAE spinal cord and multiple sclerosis brains results in the epigenetic repression of anti-ferroptotic pathways and thereby destabilizes the cellular redox balance that eventually promotes ferroptosis [521]. Accordingly, administration of an G9a inhibitor as intervention in EAE mice led to the restoration of anti-ferroptotic gene expression, a decrease in inflammation-induced neuronal loss, and an improvement in clinical outcomes.

Together, depending on the underlying disease, disease stage and the specific tissue microenvironment, ferroptosis might either contribute to tissue damage and inflammation or aid in tissue repair and inflammation resolution. In addition, it must also be distinguished whether ferroptosis plays a dominant role in the initiation of inflammation or rather in the perpetuation and exacerbation of ongoing inflammation and tissue damage. Therefore, further studies are required to identify disease-specific ferroptotic mechanisms to develop disease and context-dependent therapeutic regimens.

### 4.4 Neurodegeneration

Traumatic brain injury (TBI) and neurodegenerative diseases such as Alzheimer's disease (AD), Parkinson's disease (PD), motor neuron disease, frontotemporal dementia, and Huntington's disease contribute a major health and economic burden to humanity. The initiating causes of these diseases include complex genetic and environmental lesions that may differ according to disease, but they are unified by iron elevation and neuronal loss, which implicates ferroptosis as a common mechanism of neurodegeneration. Here, we profile the evidence linking ferroptosis to AD, PD, and TBI and associated therapeutic opportunities.

#### 4.4.1 Alzheimer's disease

New anti-amyloid therapies for AD offer modest clinical benefit yet fail to slow neurodegeneration [525]. Iron elevation in AD is associated with faster rate of cognitive decline and neurodegeneration [526,527], which is reminiscent of ferroptosis where iron confers sensitivity to inducers of ferroptosis. The AD brain also displays low GSH [528] and increased oxidative stress markers [529], which are also features consistent with ferroptosis.

The genetics of AD also points to a role for ferroptosis in this disease. Allelic variation in the *APOE* gene confers the greatest risk for sporadic AD, with the *ε*4 allele conferring increased risk, while *ε*2 is protective and *ε*3 is benign. The reasons for this are unclear. The gene product, APOE E is the major cholesterol transporter of the brain, but also signals via APOE receptors. Indeed, APOE E is a potent inhibitor of cysteine-deficiency induced ferroptosis [530]. APOE E inhibits the degradation of ferritin that releases iron during ferroptosis, by activating the Dab1/PI3K/AKT/mTROC1 pathway. All three isoforms have the same potency against ferroptosis, but the *ε*4 isoform may confer risk via orthogonal mechanisms. For example, APOE *ε*4 carriers have lower levels of APOE E [527], iron elevation in AD is greater in *ε*4 carriers [527,530]. In addition, APOE E is suggested to affect selenium uptake via trapping LRP8 in late-onset Alzheimer disease [531].

Presenilins 1 and 2 are a major cause in initiation of familial AD. These two proteins can insert interchangeably into the gamma secretase complex where they act as the catalytic subunit. Gamma secretase cleaves type 1 membrane proteins including the β-amyloid (Aβ) precursor protein, which liberates the Aβ peptide that is the major component of Aβ plaque. The Aβ peptide can be cut to varying lengths, with longer versions (≥42 amino acids) being more hydrophobic because of isoleucine 41 and alanine 42 and prone to aggregate compared to shorter versions (≤40 amino acids). There are more than 200 mutations in the presenilin proteins that cause familial AD, and the prevailing view is that they confer risk by increasing the ratio of longer form-Aβ. Yet it would be surprising if more than 200 mutations all caused this same alteration in production dynamics, since reduced function is the more likely outcome of a mutation than change in function. Indeed, when the mutations have been systematically investigated, the mutations do not consistently alter the Aβ ratio, and more than 90 % of mutations lower Aβ levels [532].

Loss of presenilin function may therefore contribute to the risk for AD. Knockout of presenilin 1/2 - and disease-causing mutations - lower GPX4 in neuronal cultures and increase susceptibility toward ferroptosis [533]. Low levels of GPX4 in these cells may be attributed to low levels of selenium, which is in-turn attributed to low levels of the APOER2, also known as LRP8. APOER2 facilitates selenium uptake in neurons, where it binds to the transporting protein, SELENOP, to permit cellular entry (see chapter 2.2.4). Presenilin-containing gamma-secretase also cleaves a type 1 membrane protein termed NOTCH, and the resultant intracellular fragment termed notch intracellular domain (NIC), activates gene transcription of a variety of genes, including APOER2. Therefore, low functioning of presenilin disables APOER2-dependent selenium uptake that is utilized by GPX4 in the defence of ferroptosis.

The connection of AD genes with ferroptosis risk coupled with iron and oxidative stress changes in the AD brains highlights the potential for ferroptosis as therapeutic target for this disease.

#### 4.4.2 Parkinson's disease

Nigral iron accumulation is a consistent pathological feature of PD and is associated with disease severity, as demonstrated using various techniques such as post-mortem tissue metal analysis, transcranial sonography, and magnetic resonance imaging [534]. Specifically, the significant shift in ferrous iron to ferric iron and increased ferritin levels with reduced GSH content in the substantia nigra [535] may promote susceptibility to ferroptosis if not directly activating the pathway. Interestingly, iron accumulation in PD resembles that in AD, where tau and amyloid precursor protein interact and their impaired functions prevent iron export, causing downstream parkinsonism phenotypes [536]. Reduction of tau results in iron accumulation, age-dependent l-dopa responsive motor deficits, and loss of substantia nigra dopaminergic neurons. Excess iron in the substantia nigra accelerates the spontaneous oxidation of dopamine to form toxic electron-deficient dopamine quinone [537]. Dopamine quinone binds to GPX4 and primes GPX4 for ubiquitination and degradation, and therefore promotes ferroptosis in the substantia nigra [537]. On the other hand, dopaminergic neuron-specific loss of *Gpx4* in mice is well tolerated and fails to cause a loss of this type of neurons in the CNS [538]. The substantia nigra contains both high concentrations of iron and dopamine, which differentiates its biochemical profile from neighbouring regions, such as the ventral tegmental area. Therefore, the interactions between iron and dopamine in the ferroptotic pathway may explain an increased selective vulnerability of dopaminergic neurons in the substantia nigra in PD.

α-Synuclein (encoded by *SNCA*) was recently found to regulate the synthesis of ether-linked PLs and thereby impact the sensitivity of dopaminergic neurons to ferroptosis [539]. *SNCA* triplication, as found in patients, significantly promoted ferroptosis induced by RSL3, associated with increased lipid peroxidation even at baseline levels [539]. Mice overexpressing α-synuclein A53T exhibited parkinsonism phenotypes, which can be attenuated by ferroptosis inhibitors and promoted by ferroptosis activation [537]. Mechanistically, endogenous α-synuclein participates in the PUFA synthesis pathway, in conjunction with ACSL4 [539]. Indeed, ACSL4 was elevated in the substantia nigra of PD and in response to 1-methyl-4-phenyl-1,2,3,6-tetrahydropyridine intoxication in mice, whereas reduction of ACSL4 protects against dopaminergic neuronal loss and motor deficits that are specifically linked with lipid peroxidation [540]. Interestingly, the use of drugs targeting ACSL4, such as the antidiabetic class of compounds known as glitazones, was associated with a reduction in the incidence of PD in a retrospective cohort study [541], providing further support for ACSL4 as a potential therapeutic target of PD. Several genes associated with a higher PD risk have also been identified to impact the susceptibility of ferroptosis. Parkinsonism associated deglycase, also known as DJ-1, a mitochondrial ROS scavenging protein where mutations cause an autosomal recessive form of PD, suppresses ferroptosis by preserving transsulfuration-mediated GSH synthesis, where its reduction results in increased lipid peroxidation that may contribute to neurodegeneration [542].

Iron chelation therapy was, therefore, trialed in PD without co-treatment of l-DOPA in a Phase IIb/III study [543]. While the iron content decreased and atrophy of the putamen was reversed by deferiprone, worse scores in measures of parkinsonism were observed in deferiprone-treated patients compared to placebo after 36 weeks. This result highlights the possibility that iron chelation therapy, while lowering the susceptibility to ferroptosis, also induced functional dopamine depletion evidenced by significantly elevated plasma prolactin levels [543]. It is also conceivable that iron chelation therapy may be more responsive in the early stage of PD, whereas at later stages, dopamine supplementation is necessary to maintain brain functions. Considering that more than 50 % of dopaminergic neurons are usually lost when PD symptoms emerge, it is also possible that the protection offered by iron chelation may require earlier treatments to achieve therapeutic benefit.

#### 4.4.3 Traumatic brain injury and endogenous brain repair

Neurodegenerative diseases, aging, and acute brain injury share common features such as inflammation, elevated oxidative damage, and atrophy predominantly of neurons, followed by a second wave of death in the wider area surround the injured site (penumbra). TBI is one of the most common pathological conditions, caused by mild or severe concussions inflicting brain damage. The first damage is due to the physical trauma, which provokes the disruption of the cytoskeletal organization of the brain tissue and – amongst others - the rupture of the blood-brain barrier. Blood extravasation and lack of oxygen and nutrient perfusion lead to secondary damage including a strong glial reactivity mediating inflammatory processes and excessive oxidative stress. Blood-brain barrier loss also raises the levels of free iron in the brain and leads to increased Fenton chemistry and peroxidation of PUFAs. If intracellular antioxidant defenses fail to restrain lipid peroxidation of cellular membranes, ferroptosis of neurons ensues. A contribution of ferroptosis to neuronal loss upon TBI is further supported by neuroprotection and improved TBI outcomes upon inhibiting ferroptosis. This was demonstrated by the use of the iron chelator deferoxamine [544], upregulation of NRF2 [545], and the application of the ferroptosis inhibitor ferrostatin-1 [546].

Interestingly, ferroptosis is not only limiting neuronal survival, but also when replacing dead neurons using direct neuronal reprogramming. Since neurons are postmitotic and cannot be spontaneously replaced in mammals including humans, the only replacement options are transplantation of exogenous neurons or converting resident glial cells into new neurons [547]. The later approach has the great advantage that the patients’ own cells are converted to new neurons *in vivo*, thereby avoding the use of immunosuppressants. For direct neuronal reporgrammining, transcription factors important for neurodevelopment, e.g. the potent proneural factors neurogenin, neuronal differentiation 1, or achaete-scute family BHLH transcription factor 1, are expressed for instance in astrocytes, which is sufficient to convert a fraction of them to functional neurons, both *in vitro* and *in vivo* [547]. Although early approaches were rather inefficient, the discovery that ferroptosis vulnerability is a major culprit in direct reprogramming afforded the efficient conversion of astrocytes from a few percent using conventional methods to a staggering 90 % of neurons *in vivo* with only one single proneural factor [548]. This breakthrough in direct neuronal reprogramming highlights that cellular metabolism and antioxidant defenses play a major role in the successful conversion of astrocytes into neurons. More recent work showed that a key organelle governing both of these processes, the mitochondria, differ profoundly (by a fifth of the entire proteome) between astrocytes and neurons, both *in vitro* and *in vivo* [549,550], with their conversion being delayed when astrocytes turn into functional neurons. CRISPRa-mediated activation of just a single neuron-enriched mitochondrial protein is already sufficient to improve neuronal conversion [549]. Most strikingly, when mitochondrial antioxidant proteins of the same family, e.g. microsomal GSH S-transferases 1 or 3 were activated during reprogramming, only the one enriched in neurons improved neuronal reprogramming, while the other failed to do so [549]. Thus, it seems there is a high degree of cell-type specific expression of antioxidant proteins that is not yet fully understood. Notably, astrocytes exhibit higher expression levels of certain ferroptosis-suppressor proteins like FSP1, FTH1 and peroxiredoxin 6 compared to neurons [549]. Inversely, neurons show greater abundance of ferroptosis-drivers such as ACSL4, aconitase 1, and IDH1 in comparison to astrocytes [549].

**Fig. 16:**
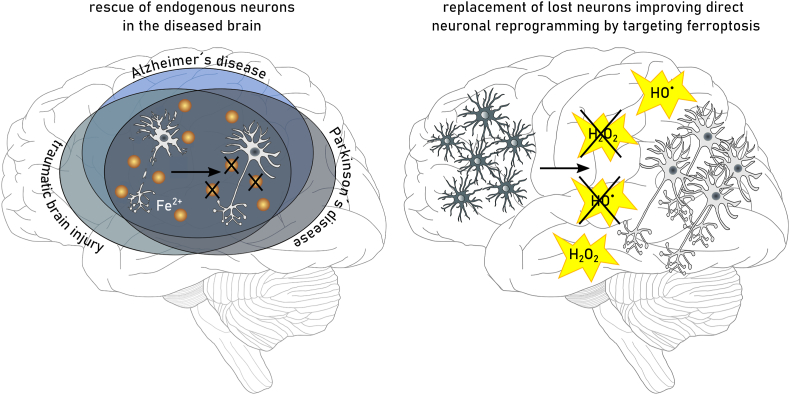
**Schematic representation of ferroptosis impairing the diseased brain, and its potential targeting as a novel approach for neuronal replacement.** Astrocytes: dark grey, neurons: bright grey.

While the exact pathways regulating ferroptosis in direct neuronal reprogramming and neurodegenerative disease remain to be unraveled, controling ferroptosis holds great promise as a drug target for neuronal replacement therapy as well as for preserving existing neurons from succumbing to death in TBI and many other neurodegenerative diseases (Fig. 16)

### 4.5 Kidney injury

Kidney tubules exhibit exquisite sensitivity to IRI (see chapter 4.2) and primary renal tubular epithelial cells are highly vulnerable to ferroptosis. This was first described for isolated renal tubules in 2013 [30] and a year later for IRI in wild type mice [34]. Along similar lines, *Gpx4*^*fl/fl;Rosa26_CreERT2*^ mice [6] succumb to spontaneous tubular necrosis followed by fulminant renal failure within two weeks after whole-body knockout induction by tamoxifen application. The lethal phenotype could not be rescued by co-deletion of *Alox*12/15, but was delayed by liproxstatin-1. In line with these results, a low residual GPX4 activity in *Gpx4*^flox/cys;Rosa26_CreERT2^ mice (exchange of the catalytic selenocysteine to cysteine on the residual allele) was sufficient to prevent spontaneous renal failure after tamoxifen application, whereas *Gpx4*^flox/ser;Rosa26_CreERT2^ mice (lacking residual activity) failed to prevent spontaneous tubular necrosis [72]. Since mice constitutively expressing a GPX4^cys/cys^ variant are not viable when kept on a congenic background [72], these data point to a crucial role for full GPX4 activity during embryogenesis, whereas residual activity might be sufficient to prevent tubular injury under steady-state conditions. Until now, it is unclear whether either genetic deficiency of enzymes of lipid metabolism such as ACSL4 and MBOAT1/2 or overexpression of FSP1 could rescue the lack of renal GPX4 activity; however, rosiglitazone, which as a side effect also inhibits ACSL4, slightly delayed lethality of Tamoxifen-induced *Gpx4*^*fl/fl;Rosa26_CreERT2*^ mice [116]. Finally, other selenocysteine containing enzymes, such as TrxR, may contribute to ferroptosis surveillance after birth, although direct evidence has not yet been established.

Whereas protective effects of lipophilic RTAs in renal IRI have been corroborated by genetic approaches, this type of evidence falls short for most other models of acute kidney injury.

Clinically, acute kidney injury due to myoglobin accumulation in the tubular system caused by rhabdomyolysis is a common phenomenon. Although its pathophysiology is highly suggestive of ferroptosis, so far, only pharmacological approaches demonstrated protective effects in this model [551], while genetic evidence is entirely lacking. In the model of folic acid-induced kidney injury, ferrostatins have been reported to be protective; yet, genetic evidence has simply ruled out necroptotic cell death [552]. Besides, the role of ferroptosis in cisplatin-induced acute kidney injury remains an area of debate. In this model, an overdose of the widely-used chemotherapeutic agent cisplatin is applied intraperitoneally, which induces renal tubular cell death (including caspase-dependent apoptosis) in a highly reproducible manner. Whereas some publications describe protective effects of pharmacological interference with ferroptosis in this model, genetic approaches utilizing *Gpx4*^*flox/cys;Rosa26_CreERT2*^ or *Aifm2*^*−/−*^ mice exhibited no differences in susceptibility compared to wild-type littermates [73].

**Fig. 17:**
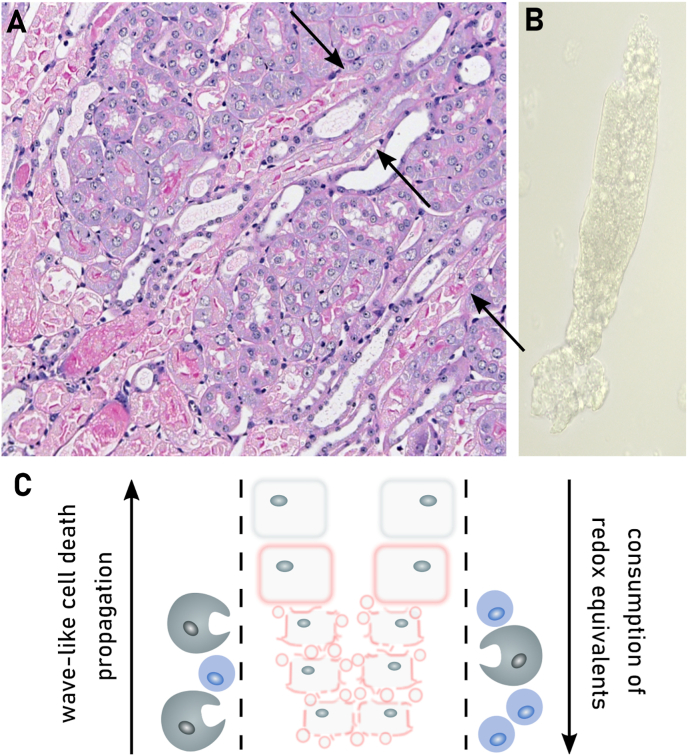
**Wave-like cell death propagation along renal tubules.** A) Following murine renal ischemia/reperfusion injury, typical tubular necroses can be observed. Arrows demark transition zones where ferroptotic cell death is just ongoing. B) A typical granular cast as observed in a patients urine sediment considered as an equivalent of acute tubular necrosis. C) Schematic depiction of acute tubular necrosis, which follows the consumption of redox equivalents. Note the innate immune cells, which are specifically recruited to necrotic areas.

In summary, ferroptosis has been demonstrated to be involved in acute tubular injury induced by various stimuli (Fig. 17), although some of them still require confirmation by genetic means. Outside the renal tubular system, compelling evidence is lacking for a role of ferroptosis in glomerular or renal interstitial or renal vascular diseases. But why are renal tubules more sensitive to ferroptosis than the remainder of the kidney or other solid organs? This question may partially be answered given the unique tendency of ferroptotic cell death to propagate through renal tissue in a wave-like pattern. Such a phenomenon has not been described for other cell death modalities. Ferroptotic cell death propagation was first observed in isolated murine renal tubules perfused with erastin [34], and has later been shown to already occur spontaneously upon manual tubule preparation without requirement of a ferroptosis inducing agent. Outgrowth cultures from freshly isolated murine tubules retain the non-random pattern of cell death propagation, e.g. when treated with RSL3 [73]. Mechanistically, the regulation of cell death propagation remains obscure. Intercellular channels such as gap junctions were discussed to be involved that might facilitate the transfer of intracellular ROS or short-lived lipid hydroperoxides as well as NAD(P)H. Some evidence suggests a preceeding calcium wave before membrane rupture, but this may only reflect the effects of high-molecular weight osmoprotectants PEG 1450 and PEG 3350 on delayed solute and lethal dye diffusion [269]. In any case, the osmoprotectants only slowed, and failed to abrogate ferroptosis propagation in U937 cells, whereas both lipophilic RTAs and iron chelators halted this process. Of note, the movement of the prominent endolysosomal compartment of freshly isolated tubules is abruptly abrogated minutes before membrane rupture. Lipid peroxidation of adjacent membranes of neighbouring cells might additionally function to propagate within membranes and ultimately contribute to membrane rupture. Lastly, beside classical approaches to induce ferroptosis, nanoparticles have been used to induce wave-like ferroptosis propagation [553].

From clinical practice, it is well understood that necrotic kidney tubular debris, referred to as “muddy brown casts”, can be found in the urine upon acute kidney injury involving ischemic events. It is tempting to speculate that these represent remnants of ferroptotic tubules which died by ferroptosis in a wave-of-death pattern. Renal histologies after experimental IRI demonstrate obstructive casts morphologically akin to muddy brown casts, which occur less frequently upon treatment with ferrostatins [34]. Recently, two-photon microscopy of murine kidneys undergoing partial IRI allowed for the first direct observation of cell death propagation during tubular cast formation inside an animal [554]. Although the efficacy of ferroptosis inhibitors was not tested in that study, this experiment may argue against a hypothesis that favors the occurrence of cell death propagation only upon hyperoxia. Another limitation is that only the most proximal segments were monitored due to technical limitations, whereas the most ferroptosis-prone S3 segments of the proximal tubules were not assessed. *In vivo*, additional factors adaptively modulate the sensitivity to ferroptosis, potentially controlling for renal tubular cell death as well. Steroid hormones are a prime example of this, opening up a new avenue on hormonal control of ferroptosis. The artificial glucocorticoid dexamethasone has been shown to inhibit ferroptosis as well as to ameliorate renal IRI [555]. The control of MBOAT1/2 expression by sex hormones (see above) might imply additional hormonal regulatory mechanisms, as initially demonstrated for hormone producing cancers [556], also in renal tubular cells. The most intriguing and entirely unaddressed question deals with the consequences of tubular necrosis. Which factors decide on complete tubular regeneration vs. permanent nephron loss? Clearly, the distinction between the two is downstream of acute tubular necrosis.

## 5 Ferroptosis in non-vertebrate organisms – potential *in vivo* models

### 5.1 Ferroptosis in microorganisms

The main prerequisites of ferroptosis and its regulation are not equally distributed in all kingdoms of life. Whereas iron and thiols can be found in all organisms, PUFAs are not present in membranes of prokaryotes. Instead, the membranes of these organisms and some single cellular eukaryotes such as yeast contain saturated or monounsaturated lipids that are not readily oxidized. However, there are some exceptions: Cyanobacteria contain PUFA enriched thylakoid membranes. After heat shock (HS), *Synechocystis* sp. undergoes a cell death process that can be prevented by canonical ferroptosis inhibitors. The pathway shares many of the essential characteristics of ferroptosis and is characterized by specific lipid peroxidation in the thylakoid membrane [45]. Other microorganisms are able to incoorporate PUFAs. Vibrio strains can incorporate PUFAs into membrane PLs [557], and *Saccharomyces cerevisiae* grown in the presence of linolenic acid show higher lipid peroxidation [558].

There are also organisms lacking GSH. *Trypanosoma brucei*, the eukaryotic single cell parasite causing sleeping sickness in humans, contains trypanothione (N1, N8-bis(glutathionyl)spermidine) instead of GSH, a single mitochondrion, and two non-selenium GSH peroxidase-type enzymes in the cytosol reducing lipid hydroperoxides [559]. Trypanosomes lacking these peroxidases die upon increased lipid peroxidation, especially in the mitochondrial membrane. Different kinds of ferroptotic inhibitors can rescue the parasites [9].

In another unicellular eukaryote, *Schizosaccharomyces pombe*, deletion of a mitochondrial translation factor disturbs iron and redox homoastases leading to higher lipid peroxidation. The resulting cell death was rescued by ferroptosis inhibitors [560]. Moreover, treatment with iron or erastin was suggested to induce microbial ferroptosis in *Pseudomonas aeruginosa* [561], *Staphylococcus aureus* [562,563], and *Vibrio* sp. [563,564]. These observations indicate alternative pathways to induce ferroptotic cell death and render microorganisms potential alternative *in vivo* models to investigate new routes and the evolutionary aspects of ferroptosis.

### 5.2 Ferroptosis in plants

**Fig. 18:**
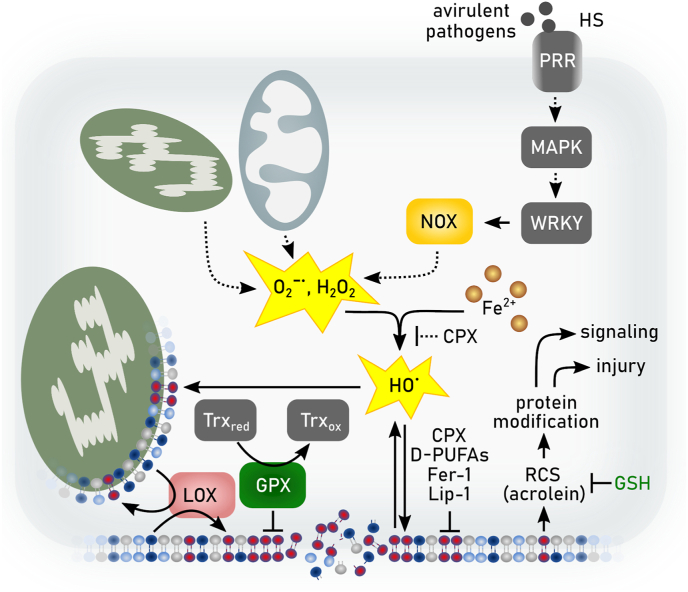
**Proposed model for plant ferroptosis.** Ferroptosis can be induced by avirulent pathogens or by heat stress (HS) via pattern recognition receptors (PRR). After an attempted infection, a MAPK cascade is activated, phosphorylating WRKY transcription factors which in turn induce NOX expression, leading to ROS accumulation. The pathway involves GSH depletion, ROS accumulation and iron-dependent lipid peroxidation. Cytoplasmic ROS accumulation might occur through NADPH oxidase activity (NOX) and by the generation of toxic lipid‐peroxides. Those lipid peroxides could originate from enzymatic or non‐enzymatic processes on membrane poly-unsaturated fatty acids (PUFA-PLs) from chloroplasts and cytoplasmic membrane, involving either lipoxygenases (LOXs) activity or Fenton chemistry. Lipid ROS can also be degraded to reactive carbonyl species (RCS), such as acrolein, which are also related to cell death. Glutathione peroxidases (GPXs) are proposed to act as negative regulators, detoxifying the cell from lipid peroxides. In plants, GPXs uses thioredoxin (Trx) as a main reductant agent, but they might also utilize GSH. Ciclopirox olamine (CPX), D-PUFAs, ferroststin 1 (Fer-1), and liproxstatin-1 (Lip-1) inhibit ferroptosis. RSL3 induces ferroptosis. Dashed lines indicate indirect evidence.

Plant ferroptosis was first described in *Arabidopsis thaliana* roots after HS. The regulated cell death pathway that followed HS displayed all the characteristic features of ferroptosis, such as iron-dependent lipid peroxidation and GSH depletion. Additionally, this form of cell death was prevented by canonical ferroptosis inhibitors like ferrostatin-1 and ciclopirox olamine [565] (Fig. 18). Since then, additional studies in plants have shown the importance of ferroptosis in other processes and plant species, e.g. wheat roots [566] and *Chlamydomonas reinhardtii* [567], especially in plant-pathogen interactions. Furthermore, ferroptosis inhibitors protect *Nicotiana benthamiana* plants from accelerated cell death triggered by a tobacco mosaic virus (24A+UPD strain) [568]. Ferroptosis seems also to be relevant in the interaction between rice and the fungus *Magnaporthe oryzae*, which results in an oxidative iron-dependent cell death that was prevented by treatment with ferroptosis inhibitors [569,570]. In addition, ferroptosis plays a role in the defense mechanism against oomycete infection of soybean plants [571]. Upon infection, typical ferroptotic hallmarks were detected in soybean hypocotyl cells, including decreased GSH levels, accumulation of ferric ions, and lipid peroxidation, which were attenuated by ferroptosis inhibitors. Another study suggested that acrolein, a carbonyl species derived from lipid peroxides, might also have a role in ferroptosis, as acrolein induced cell death can be prevented by ferroptosis inhibitors [572]. Also, treatment with the chiral herbicide (R)-dichlorprop triggers ferroptosis in *Arabidopsis thaliana*. Iron dependent formation of lipid hydroperoxides, and malondialdehyde was observed upon (R)-dichlorprop application [573]. As was observed for plants after HS, (R)-dichlorprop also induced GSH and ascorbic acid depletion and the accumulation of lipid hydroperoxides. As far as is known, plant ferroptosis takes place in response to both abiotic and biotic stresses and there is no evidence that links ferroptosis with development related, regulated cell dead pathways [565].

In plants, ROS accumulation is one of the earliest recognized biochemical events that take place soon after ferroptosis induction. Cytosolic ROS increases shortly after HS in *Arabidopsis* and can be consistently measured from 15 min to 3 h after treatment. Pre-treatment with the canonical NOX inhibitor diphenylene iodonium prevents both cytosolic ROS accumulation and cell death [565]. Additionally, ferroptosis triggered by pathogen infection is also accompanied by a massive burst of ROS and the activation of WRKY transcription factors that subsequently induce NOXs expression [570,571]. Although mitochondrial ROS have been largely associated with plant cell death pathways, there is not clear evidence indicating a role for mitochondria on ROS production during plant ferroptosis [574]. However, chloroplasts might play a role in the oxidative burst that follows HS in plants undergoing ferroptosis. Plants treated with high temperatures die at higher rates when they are exposed to light, suggesting that active chloroplasts are contributing to cell death in leaves, although the basis of this contribution is still a matter of study [565]. In addition, the chloroplast can act as a source of iron required for ferroptosis. In this sense, ferritins, which function as major iron storage molecules in chloroplasts, have been shown to play a crucial role in the regulation of ferroptosis. In rice, the expression of ferritin 2 (OsFER2), positively regulates ferroptotic cell death in the rice - *M. oryza*e interaction and iron released from OsFER2 was proposed to be required for the activation of a MAP kinase cascade that triggers the expression of defense-related genes [570]. On the contrary, OsFER2 knockout plants show reduced ROS production and inhibition of ferroptotic cell death.

As present in animal cells, the accumulation of lipid peroxidation products is also a hallmark of plant ferroptosis. In plants, the inhibition of PUFA peroxidation prevents HS-induced ferroptosis [575]. However, subcellular localization and direct lipid peroxidation target are yet to be identified. Lipid hydroperoxides can be produced either by enzymatic and by non-enzymatic lipid peroxidation. It has been known for many years now that lipid peroxidation mediated by lipoxygenase play a part during the cell death processes triggered by both biotic and abiotic stresses. Interestingly, Christensen et al. [576] showed that the activity of 9- lipoxygenase on linolenic acid produces 10-oxo-11-phytotenoic acid as well as 12- and 14-carbon cyclopente(*a*)nones, which are named “death acids” that regulate the expression of genes related to defense and that promote cytotoxicity. In addition, iron can also bind to PCBPs, which are capable of transferring iron to lipoxygenase implicated in ferroptosis. Supporting a similar role for these proteins in plants, a strong induction of a PCBP protein has described in *Nicotiana benthamiana* during ferroptosis induced by viral infection [568]. Lipid hydroperoxides can also be produced by non-enzymatic routes. Supporting a contribution of Fenton chemistry in plant ferroptosis, accumulation of Fe^3+^ and peroxides was found associated to cell death sites in resistant rice after the infection with an avirulent strain of *M. oryzae* [569].

Lipid peroxidation could persist and diffuse trough lipid bilayers, or can be degraded to aldehydes (e.g., acrolein) or hydroxy acids, which are also reactive molecules known as reactive carbonyl species (RCS). RCS have been largely related to plant cell death pathways. Specifically, BY-2 tobacco cells treated with acrolein undergo a cell death process that can be prevented by co-treatment with ferroptosis inhibitors and GSH [572]. Because RCS are relatively stable molecules in comparison to ROS, they have been proposed as suitable signals modulating different aspects of plant physiology, including cell death. Thus, RCS appear as suitable signal molecules that might help to propagate the ferroptotic signal between cells, as hydroperoxides do in tumor cells [45].

### 5.3 Non-mammalian *in vivo* models to study ferroptosis - *Drosophila melanogaster*

Still little is known about the organismic effects of ferroptosis due to the lack of suitable and well-characterized animal models. The fruitfly *Drosophila melanogaster* would constitute such a model owing to its short lifespan, well-characterized genome, genetic manipulability, and conservation of core cell death pathways [577], but only very few and limited studies have yet tried to elicit ferroptosis in the fly. Wang et al. reported that neural overexpression of mitochondrial ferritin increases the survival of flies treated with erastin, but whether erastin indeed results in ferroptosis and how mitochondrial ferritin interferes with it was not further clarified [185]. Others tested the effect of ferroptosis inducers (erastin and ferric ammonium citrate) and inhibitors (N-acetylcysteine and deferiprone) in flies overexpressing the human protein α-synuclein, a synaptic protein implicated in Parkinson's Disease (see chapter 4.4). Although flies do not harbour α-synuclein, forced overexpression in dopaminergic neurons results in reduced lifespan, impaired locomotion, and loss of dopaminergic neurons. Here, additional induction of ferroptosis aggravated the pathological phenotype, while inhibition ameliorated most of these symptoms [578]. Again, no downstream effects of ferroptosis induction were studied. While these results indicate a potential role of ferroptosis in *Drosophila*, they lack a comprehensive analysis of ferroptotic hallmarks. To establish the fruit fly as an additional *in vivo* model, the effect of pharmacological and genetic induction of ferroptosis on the recognized ferroptotic hallmarks — GSH levels, lipid peroxidation, and redox state of cellular iron — need to be studied in more detail.

## 6 Methods to reliably detect ferroptotic cell death

### 6.1 Mass spectrometry

Ferroptosis is induced by the occurrence of (phospho)lipid hydroperoxides resulting from defects in cellular redox homeostasis. Therefore, the analysis of redox-regulating biomolecular status is essential for understanding the precise mechanisms of ferroptosis in different biological contexts. This understanding has been widely achieved through mass spectrometry-based analytical methods [[50], [51], [52], [53],^230^,^232^]. Antioxidant molecules often exist in both reduced and oxidized forms, requiring additional efforts to prevent further oxidation or interconversion during sample preparation and instrumental analysis. Moreover, due to the diverse chemical nature of redox-active molecules, various chromatographic separation strategies, such as normal-phase liquid chromatography (NP-LC), reverse-phase liquid chromatography (RP-LC), or gas chromatography, need to be implemented prior to mass spectrometry analysis to achieve the necessary specificity among the complex matrices of biological samples. Here, we describe the analytical methods that have been employed to determine the redox status of molecules, shedding light on the understanding of ferroptosis execution. Additionally, we explore advanced mass spectrometry MS)-based analytical techniques that have substantially contribute to our current understanding of ferroptosis.

Cystine deprivation induces ferroptosis by depleting cellular GSH, which exacerbates oxidative stress and lipid peroxidation [5,232]. This highlights the significance of cysteine metabolism in understanding the basic principles of ferroptosis. Both cysteine and its downstream metabolite GSH contain sulfhydryl (-SH) residues that are oxidized to cystine and GSSG during sample preparation, respectively, preventing the analysis of their intact oxidized and reduced forms before extraction. The –SH residues can be preserved through derivatization with an alkylating reagent, e.g. N-ethylmaleimide, prior to instrumental analysis [232]. The alkylated cysteine and GSH, along with their oxidized forms cystine and GSSG, can be readily determined using NP-LC-MS [232]. Recent studies have revealed that hydropersulfides also play a role in preventing ferroptosis by capturing radicals and engaging in autolytic regeneration mechanisms. The sulfur-containing metabolites are first derivatized using another alkylating agent, i.e. monobromobimane, and analyzed through NP-LC-MS [230].

CoQ_10_ and vitamin K scavenge (phosphor)lipid peroxyl radicals to inhibit the lipid peroxidation chain reaction and ferroptosis [[50], [51], [52]]. FSP1 reduces both CoQ_10_ and vitamin K, producing their hydroquinone products, CoQ_10_H_2_ and vitamin KH_2_, respectively. While hydrophobic CoQ_10_ and CoQ_10_H_2_ are well detectable by RP-LC-MS, CoQ_10_H_2_ undergoes fast oxidation to CoQ_10_ after extraction from biological samples. Therefore, current analysis methods rely on antioxidants in the extraction solvent with higher reduction potential than CoQ_10_H_2_ or to protect CoQ_10_H_2_ against oxidation, such as butylated hydroxytoluene [50]. Meanwhile, Mishima et al. determined a function of FSP1 in producing vitamin KH_2_ from vitamin K, employing flow injection MS with the advantage in preventing vitamin KH_2_ oxidation due to its fast instrumental analysis. Vitamin KH_2_ further oxidizes to vitamin K epoxide via γ-glutamyl carboxylase, catalyzing the carboxylation of coagulation factors. The accumulation of vitamin K-epoxide from vitamin K in biological samples can be measured using gas chromatography-MS [52]. Future methods that enable simultaneous determination of vitamin K, vitamin KH2, and vitamin K-epoxide will provide a better understanding of the Mishima cycle status in biological samples. BH4 is another redox-active compound that directly protects against lipid peroxidation, and that can be regenerated by DHFR. Similar to CoQ_10_ analysis, ascorbic acid and dithiothreitol are included in the extraction solvent as antioxidants to maintain BH4 in its reduced state, followed by NP-LC-MS analysis [53].

The ratios of NADPH to NADP^+^ and NADH to NAD^+^ play a role in regulating redox homeostasis by acting as cofactors for ferroptosis-preventing antioxidant enzymes, including FSP1, DHFR, and other antioxidant proteins. Interconversion of these NAD redox cofactors during extraction can prevent accurate measurements of their reduced and oxidized forms. Lu et al. discovered that adding 0.1 M formic acid to the extraction solvent minimizes this interconversion. Due to their hydrophilic chemical properties, NAD redox cofactors are well-suited for analysis using RP-LC-MS with ion-pairing reagents and NP-LC-MS [579].

In addition to targeted measurements of redox metabolites, LC-MS-based global metabolomics has been employed to uncover novel metabolic pathways involved in ferroptosis regulation. NP-LC-MS-based global metabolomics approaches have revealed that BH4 is the most significantly accumulated metabolite in ferroptosis-sensitive cancer cell lines compared to ferroptosis-resistant cell lines [53]. Similarly, the accumulation of gamma-glutamyl-amino acids has been observed in cystine-deprived cancer cells, hinting at a non-canonical role of GCLC in preventing glutamate accumulation-mediated oxidative stress in cystine-deprived cells [232]. On the other hands, stable isotope-labeled metabolite tracing approaches have proven valuable in measuring precise metabolic pathway activity in ferroptosis regulation. Tracing with ^13^C-labeled serine can evaluate cysteine metabolic pathway activity, including transsulfuration-based cysteine synthesis and GSH synthesis pathways [232]. Similarly, ^13^C-labeled glucose tracing can measure CoQ_10_ synthesis activity [93]. Furthermore, ^13^C-labeled linoleic acid tracing provides insights into the activities of the fatty acid elongase and desaturate pathway, an active inducer of ferroptosis [114].

Nano-flow RP-LC-MS enables the analysis of thousands of proteins in a few hours. Beyond metabolite analysis, various proteomics approaches have been employed to study ferroptosis. Global proteomics analysis has led to the discovery of NCOA4's role in inducing ferroptosis through increased intracellular ion accumulation [580]. Chemoproteomics approaches have been developed to study comprehensive lipid-derived electrophile-mediated protein carbonylation during ferroptosis [581]. Further, targeted redox-proteomics approaches have revealed the precise mechanism of irreversible cysteine oxidation in the cysteine homolog of GPX4, emphasizing the indispensable role of selenium in the active site of GPX4 to sustain its full enzymatic activity [72]. Similarly, redox-proteomics platforms that measure the redox status of various proteins may provide valuable insights for a better understanding of ferroptosis in the future.

In conclusion, MS-based analytical methods have been applied to determine various biomolecules regulating redox homeostasis, offering insights into the mechanisms of ferroptosis. Integrating these analytical platforms to gain a comprehensive view of the redox status of antioxidant molecules, as well as the metabolome and proteome, will provide unique opportunities to conduct more precise evaluations of the relevance of ferroptosis in biological systems.

### 6.2 Lipidomics

The process of lipid peroxidation is central to the execution of the ferroptotic death program, and the general principle of two contributing and disarrayed metabolic pathways – (i) Fe-dependent reactions leading to the production of readily cleavable lipid hydroperoxides and (ii) the insufficiency of their reduction to stable lipid alcohols by thiol-driven metabolic mechanisms utilizing GPX4 – have been firmly established [6,74]. Initiators of lipid oxidation in ferroptosis are either Fenton chemistry and free radical driven lipid peroxidation [582] or Fe-containing oxygenases, particularly lipoxygenases 15 and 12 [110,115,120,139]. In both cases, during this stage the abstraction of a bis-allylic H-atom form the oxidizable PUFAs takes place. With regards to lipid peroxidation, one has to keep in mind that this free radical process is principally different from the traditional biochemical reactions in which enzymes utilize a limited number of substrates and generate a finite number of products. On the contrary, when triggered, lipid peroxidation will propagate over available PUFA substrates and result in a great variety of oxidized species unconstrained in terms of modification position and its stereochemistry. Simply speaking, the spectrum of lipid peroxidation products in a given biological system will be defined by its initial lipid composition (e.g., extent of PUFA chains in membrane lipids serving as lipid peroxidation substates) and redox environment (e.g., presence of transition metal ions such as Fe^2+^ and RTAs such as α-tocopherol). Although the specific reactions and their interactions driving pro-ferroptotic metabolism remain far from being clear, it is apparent that oxidized lipids serve as the executioners of this cell death modality. Thus, detection, structural annotation and quantification of oxidized lipids in pro-ferroptotic conditions is of utmost interest for the scientific community. Commonly employed techniques such as thiobarbituric acid assay for a terminal lipid peroxidation product (e.g. malonyldialdehyde), lipophilic oxidation sensitive probes such as C11-BODIPY581/591 probe and LiperFluo are indicative of the engagement of the ferroptotic process and easy to implement. However, they are insufficient for the full characterization of diversified individual pro-ferroptotic products responsible for the execution of death program itself and accompanying bio-signaling functions – e.g., immunosuppression, proliferation, differentiation [583]. Thus, MS-based methods are required to support in-depth characterization of oxidized (phospho)lipids.

Indeed, with the use of MS-based lipidomics, it has been firmly established that polyunsaturated glycerophospholipids (PUFA-PLs) and their hydroperoxy-derivatives represent the preferred substrates and products of the ferroptotic peroxidation process, respectively. More specifically, two types of PEs with arachidonoyl (20:4) and adrenoyl (22:4) residues have been identified as the major lipid peroxidation substrates generating several hydroperoxy-products in mouse embryonic cells undergoing ferroptosis [115]. Subsequent work demonstrated that the list of PUFA-substrates and products is much broader and includes, depending on cellular context, genetic and nutritional factors, a significantly more diversified PUFA-PLs such as PC, phosphatidylserine, phosphatidylinositol, cardiolipin, and bis(monoacylglycero)phosphate [1,584]. Importantly, however, the high selectivity towards di-acyl-PUFA-PE and plasmalogen-PE seems to be retained in the majority of cases of ferroptosis-associated lipid peroxidation sensitive to most commonly used inhibitors of ferroptosis, ferrostatin-1 and liproxstatin-1 [120,585]. That is not surprising, considering cell intrinsic nature of ferroptotic cell death and asymmetry of plasma membrane with its inner, cytoplasmic leaflet enriched in PUFA-PE and plasmalogen-PE, whereas outer leaflet represented by more saturated species of PC and sphingomyelins [586]. However, much less is known about the distribution of oxidized lipids in subcellular organelles, such as mitochondria and ER. Thus, despite certain advances in identification of oxidized lipids using conventional untagged or targeted lipidomics workflows, more holistic approach accompanied by detailed molecular annotation of oxidized lipids in complex biological samples is required. Detection and characterization of oxidized lipids in complex biological samples remains challenging due to their low natural abundance and extreme structural variability [587]. To simplify the detection of peroxidation products, enzymatic hydrolysis of oxidized PLs by phospholipase A2 to release oxygenated fatty acid may be used to reduce the number of the peroxidized analytes. This “simplification”, however, eliminates the “parenthood” of the peroxidized PUFA such that no information on the PL-origin of oxidized fatty acids can be obtained. Identification and quantitative assessments of oxidized PLs can be significantly improved by choosing the right techniques for sample preparation. Solid phase extraction has been successfully used for fractionation, concentration, and identification of PLs [588], and their oxidatively-modified metabolites [584]. Combination of Solid phase extraction with reverse chromatography and targeted selected ion monitoring was successfully applied to detect and structurally characterize oxidized PLs in a number of cases, for example melanoma cells *in vitro* and *in vivo* [584]. Such targeted assays are required to address low abundant species. On the other hand, only untargeted detection can provide high coverage of oxidized lipids in a given biological context. Contemporary high-resolution MS permits to separate and estimate identity/structure of lipids with very close *m/z* ratios (with ∼1 ppm accuracy). Combined with tandem MS experiments, it permits detection, identification and quantitative characterization of oxidized phospholipid species in cells and tissues. Recently, an analytical workflow has been developed which allows to address abovementioned challenges by combining *in silico* prediction of oxidized lipidome by LPPtiger2 software and LC-MS analysis [589]. LPPtiger2 is an open-source computational tool for the prediction and annotation of modified lipids from LC-MS/MS experiments. Using molecular networks covering oxidation of 10 PUFAs, LPPtiger2 can predict a set of oxidized lipids using a given lipidome as a substrate. This unique algorithm enables to address the challenge of unknown diversity of oxidized lipids, as the composition of the initial cell/tissue specific lipidome will define the composition of its oxidized lipidome. Next, semi-targeted analysis using optimized LC-MS/MS setup tuned to sensitive and specific detection of oxidized lipids is used with the predicted set of oxidized lipids. By using optimized reverse-phase chromatography on C30 columns, not only the separation of different classes of unoxidized and oxidized lipids, but also the resolution of isomeric species can be achieved, including positional isomers (e.g., 15-OOH vs 5-OOH arachidonoyl containing lipids). Using preferential ionization modes and triggering MS/MS on lipid class adducts (e.g., sodiated adducts for oxidized triacylglycerols and cholesterol esters vs deprotonated forms of oxidized PLs) improves MS detection and MS/MS based structural annotation. Importantly, the fragmentation rules for different types of oxidative modifications covering modification type and its positions were formulated and implemented. This workflow was successfully applied in a number of ferroptotic studies including cells and animal models [52,164], and allowed to detect a rich spectrum of oxidized PC and PE lipids, including hydroperoxy, mono – and dihydroxy, keto derivatives as well as oxidatively-truncated species.

Oxidatively-truncated PL species provide an interesting example of the secondary reactions occurring under pro-ferroptotic conditions. For example, peroxidized (phospho)lipids containing carbonyl moieties can attack lysines to form Schiff bases. Furthermore, oxidatively-truncated PLs with α,β-unsaturated moieties are susceptible to nucleophilic attack by cysteines, histidine or lysines to form Michael adducts [590]. Nucleophilic moieties of PLs can also represent possible targets for interactions. Given the preferred peroxidation of PE in ferroptosis, a recently developed technique employed an antibiotic, duramycin, capable of high affinity binding ethanolamine group, to strongly enrich and identify the PE-lipoxidated proteins [591]. The assumption has been that oxidatively-truncated PUFA-PE formed during ferroptosis would covalently label nucleophilic sites in the proteins. This protocol has been applied to several cell lines challenged with a pro-ferroptotic agent and several PE-lipoxidated proteins have been detected and identified, including those present in the plasma membrane. However, whether PE-lipoxidated proteins are involved in the execution of the ferroptotic program or simply the result, remains unknown.

With advances in new analytical tools, the analysis of oxidized lipids has undergone constant development. Introduction of largely orthogonal to MS gas phase based separation of ionized lipids using ion mass spectrometry (IMS) provides new opportunities in resolving structural diversity of oxidized lipidomes. When implemented in LC-MS workflows, LC-IMS-MS methods provide an opportunity for deeper coverage and annotation. Recently, successful application of drift tube ion mobility for the analysis of oxidized lipids, including both free and PL-esterified oxidized PUFAs, has been demonstrated [592]. Furthermore, although LC-MS lipidomics established the distinct dependence of ferroptosis on PL oxidation, this approach falls short of determining which cells and subcellular compartments are involved in the initiation and subsequent stages of PL peroxidation. This information can be obtained by emerging technologies of MS imaging. Several MS imaging protocols have been developed including matrix-assisted laser desorption/ionization (MALDI), laser ablation electrospray ionization, desorption electrospray ionization, etc. These tools afford imaging of multiple biomolecules including abundant lipid species at spatial resolution of 5–200 μm [592]. A laser spot size of about 1 μm is technically possible for some MALDI instruments [593], but gathering sufficient molecular ions for imaging or performing tandem MS within a ∼1 μm is currently not feasible. Promising results with improved spatial resolution (∼1 μm) and sensitivity have been obtained by the use of secondary IMS (SIMS) coupled to high energy gas cluster ion beams (GCIB) [594]. GCIB-SIMS coupled to H_2_O clusters has enabled imaging of low abundance oxidized PE and significant changes in essential redox metabolites such as GSH associated with the execution of ferroptosis in cardiomyocytes and neurons [594]. Importantly, GCIB-SIMS imaging can be utilized in a multi-omics paradigm by additional profiling of cell-type specific lanthanide-conjugated antibodies on the same tissue section using C_60_-SIMS thus permitting identification of different types of cells undergoing ferroptosis [595].

It is also important to note, that not only the detection of oxidized lipids but also the profiling of general lipidome remodeling provides important information on the lipid metabolism and its plasticity under pro-ferroptotic stress. Thus, a number of classical lipidomic studies were performed and demonstrated significant alterations in lipid metabolism upon ferroptosis induction. Among major detected trends, a reduction in the abundance of PUFA-containing PLs, including PE, PC and phosphatidylserine lipids, were detected in several studies [120,412]. Multiple studies further highlighted the significance of lipidome unsaturation in ferroptotic cell death, by demonstrating that supplementation of cell culture medium with PUFAs (e.g., arachidonic and docosahexaenoic) sensitized cells to ferroptosis, whereas MUFA (e.g., oleic acid) supplementation attenuated ferroptosis sensitivity [111]. Thus, the degree of lipidome unsaturation can serve as a proxy for ferroptosis sensitivity in different cell types and populations. For instance, an elegant study on different populations of human and murine immune cells demonstrates a low level of unsaturation in myeloid versus lymphoid cells, and this was experimentally confirmed by the differences in their susceptibility to ferroptotic cell death [596]. Although a decrease in PUFA-PLs observed in several models of ferroptosis is usually connected with their susceptibility to oxidation, recently several studies highlighted the importance of acyl chains trafficking from membrane PLs to neutral lipids in lipid droplets as an adaptive mechanism to prevent extensive lipid peroxidation in a pro-oxidative environment. Lipid droplet accumulation was detected as a hallmark of ferroptosis in several studies, and accumulation of PUFA-rich triglycerides and cholesterol esters was demonstrated [597]. Thus, in-depth investigations of lipidome remodeling under pro-ferroptotic conditions is capable of providing multiple clues in metabolic adaptation but needs further developments in LC-MS based lipidomics and chemical biology, for instance application of biorthogonal techniques (e.g., click chemistry) approaches to track remodeling of acyl chains in complex lipidomes [598]. Two most recent studies discovered unusually high peroxidation rates of low-abundance doubly-PUFA phospholpids in ferroptotically dying cells thus making decreased levels of these newly discovered substrates a promising additional biomarker of ferroptotic PL peroxidation [106,599].

### 6.3 Biochemistry

#### 6.3.1 Fluorescent probes

Ferroptosis has three main biochemical features: (1) peroxidation of PUFA-containing PLs; (2) impaired antioxidant defence and repair systems for clearing phospholipid peroxides (both covered in this chapter); and (3) availability of redox-reactive iron (see chapter 6.3.3) [600]. Chemical probes typically assay just one of these aspects, so multiple assays are needed to corroborate that ferroptosis is indeed the underlying cell death mechanism (all three features). Assays must be well-controlled since the chemical reliability of currently available probes is limited. Finally, even well-controlled experiments must be interpreted cautiously: since probes typically react with a broader range of species than trade names or assay kits suggest; and since probes' reaction rates differ greatly for even similar species (e.g. various types of oxygen-centered radicals), let alone distantly-related ones (e.g. carbon-, nitrogen- or sulfur-centered radicals; or two-electron oxidants e.g. H_2_O_2_).

**Fig. 19:**
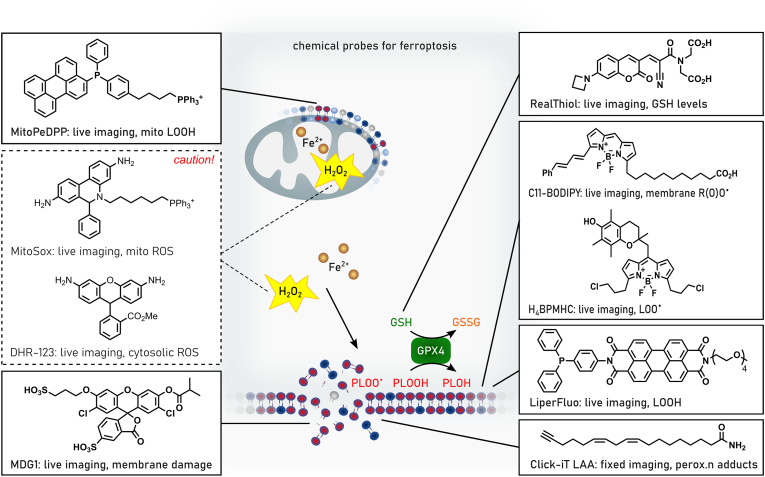
**Chemical probes for detecting ferroptosis.** These probes can report on phospholipid peroxidation (C11-BODIPY, H_4_BPMHC, LiperFluo; including in mitochondria via MitoPeDPP); loss of plasma membrane integrity (MDG1); and depletion of antioxidant defenses e.g. GSH levels (RealThiol). Fluorescent “dihydro" probes claimed to report ROS levels (MitoSox, DHR-123) should only be used with caution.

(1) ***Phospholipid Peroxidation:*** Lipid peroxidation can be followed longitudinally in live cells by probes such as C11-BODIPY 581/591 or H_4_BPMHC, that react with radicals in the membrane (detection in parallel to lipid peroxidation); or probes e.g. LiperFluo that react with lipid hydroperoxides (Fig. 19) [601]. (i) Polyenes like C11-BODIPY 581/591 can be more susceptible to oxygen-centered radicals than natural, skipped polyene PUFAs. Thus, their fluorescence reports on radicals in the membrane, but not the actual degree of lipid oxidation [602]. (ii) α-tocopherol analogues like H_4_BPMHC react with lipid peroxyl radicals, and are best understood as real-time reporters of the depletion rate of native lipophilic antioxidants [603]. (iii) Triarylphosphines like LiperFluo (a successor to 1,3-bis(diphenylphosphino)propane) react with lipid peroxides. Their fluorescence thus reports on lipid damage, but not directly on the progress of ferroptosis, since these probes cannot distinguish between different peroxides that contribute to ferroptosis to varying degrees. Such compounds have also been modified to localize them at the inner mitochondrial membrane in respiring cells, from where they can probe the same reactions with mitochondrial specificity (polyenes like MitoPerOx and MitoCLox, or triarylphosphine MitoPeDPP; Fig. 19).

Lipid peroxidation can also be assessed at endpoint in fixed or lysed cells to better study the speciation of membrane damage. (iv) MUFA/PUFA peroxidation ultimately yields end-products including aldehydes that can react with nucleophilic side chains to form protein adducts. Click-iT LAA is a diene probe that is as sensitive to peroxidation as the most abundant mammalian PUFA linoleic acid, but also contains an alkyne that allows quantifying or localising its protein adducts, as a readout for the degree or spatial localization of lipid peroxidation (Fig. 19). (v) Importantly, lipidomics can give an unbiased, probe-free quantification of lipid peroxides and their reaction products (see chapter 6.2).

(2) ***Antioxidant defence and repair:*** Lipid peroxide antioxidant and membrane repair capacity can be assessed as a proxy readout for ferroptosis, but they are also lowered in non-ferroptotic cell death, so the following should be interpreted cautiously [604]. (vi) GPX4 activity can best be assessed in cell and tissue lysates, monitoring the reduction of e.g. phosphatidylcholine peroxide or 7α/β-cholesterol peroxide either directly by chromatography, or by proxy through monitoring NADPH consumption in a GSH/GSH reductase coupled reaction [115]. (vii) Recent methods to assess GSH levels and dynamics in live cells with reversible reaction-based thiol sensors, e.g. the organelle-targeted sensor TRaQ-G [605], or pan-cellular sensor RealThiol, seem to solve several nonspecificity problems of old assays using monochlorobimane or Ellmann's reagent DTNB. Lipophilic antioxidants are covered in point (ii) above. (viii) Lipid peroxidation and downstream processes ultimately cause loss of membrane integrity and membrane rupture. While not specific to ferroptotic cell death, dyes that only enter rupturing cells, such as propidium iodide, ethidium bromide and trypan blue, are widely used, although recent nontoxic low-background probes such as MDG1 are enabling longitudinal assays in live cells and embryos [606].

##### 6.3.1.1 Chemical probes that should not be used

Lipid (per)oxidation involves highly reactive, and often non-specific and/or poorly defined molecular species. Unfortunately, many probes and assays are also nonspecific, poorly-defined, and regardless of vendors' claims or literature usage, they should be banned from publications due to their inaccuracy, cross-reactivity, and persistent misinterpretation. These include the thiobarbituric acid assay in lysates, claimed to detect the lipid peroxidation product malondialdehyde (use chromatography instead); and dihydrofluoresceins e.g. DCFH/DCFH-DA, claimed to quantify ROS (avoid in all settings [607]; it is likely that similar “dihydro" probes e.g. dihydroethidium, its mitochondrial version MitoSOX, and dihydrorhodamines like DHR-123, are similarly unfit for purpose) (Fig. 19) [608].

In sum, the diverse aspects of ferroptosis can be assayed using a variety of chemical probes. Older probes are actively curated, improved, and characterised in greater depth; and the toolset continues to grow to address unmet needs. By (a) prioritising the use of high-quality chemical probes, (b) interpreting assay results in terms of (bio)chemically defined species, and (c) using orthogonal control assays to interpret cellular responses rigorously (e.g. distinguishing ferroptosis vs necrosis, etc), biochemists and biologists can increasingly rely on chemical probes to drive high-quality basic and translational research within this dynamic field.

#### 6.3.2 Activity assay, immunohistochemistry and antibodies

Given the absence of an established single molecular marker for unequivocally detecting ferroptosis thus far, ferroptosis can be considered when the observed cell death satisfies the following criteria: evidence of cell death functionally coupled with lipid peroxidation, which is validated by rescuing cell death using ferroptosis-specific inhibitors and exclusion of other forms of cell death. Therefore, demonstrating ferroptosis still requires a multifaceted approach involving a combination of techniques and methods (Table 1).

**Table 1:** Methods for detecting ferroptosis.

**1. Detection of cell death**
Cell viability assay
Dead cell staining
LDH release
TUNEL staining

**2. Indicators of lipid peroxidation**
Fluorescent lipid peroxidation probe (e.g., C11-Bodipy 591/581)
Mass spectrometry-based (oxi)lipidomics
Byproduct of lipid peroxidation (e.g., 4-HNE and MDA)

**3. Evidence of ferroptotic cell death**
Rescuing effect by ferroptosis inhibitors (considering that majority are RTAs)
Exclusion of other cell death forms

*LDH, Lactate dehydrogenase; TUNEL, terminal deoxynucleotidyl transferase dUTP nick end labeling; 4-HNE, 4-hydroxy-2-nonenal; MDA, malondialdehyde.

##### 6.3.2.1 Detection of cell death

*In vitro* assessments of cell viability based on metabolic activity (e.g., WST-8 and resazurin assay) or cellular ATP contents are commonly used for evaluating the number of viable cells. However, when implementing such approaches, it is important to consider factors such as proliferation rates and cellular metabolic state, which can influence the assay outcome alongside viable cell number. Staining with dyes that mark cells with compromised membrane permeability are a good surrogate of cell death (e.g., propidium iodide), analogously the measurement of lactate dehydrogenase released from lysed cells offers the representation of the fraction of dying cells. *In vivo* experiments often employ TUNEL staining, detecting DNA double-strand breaks, to identify cell death. While TUNEL is considered a bona fide marker for apoptosis, it is known that TUNEL can also give positive results in other regulated cell death forms, including ferroptosis [364]. To rule out the involvement of other known cell death modalities, it is useful to verify that other cell death-specific markers (e.g., cleaved caspase-3 for apoptosis or phospho-mixed lineage kinase domain like pseudokinase for necroptosis) are not detectable and also that the findings of cell death remain unaffected by other cell death specific inhibitors (e.g., pan-caspase inhibitors to prevent apoptosis, necrostatin-1s for necroptosis). Though some of these approaches are not practical to be implemented *in vivo* due to the lack of metabolic stability of some of these cell death inhibitors.

##### 6.3.2.2 Indirect ferroptosis markers

In addition to fluorescent lipid peroxidation probes, such as C11-Bodipy 581/591, and MS-based (oxi)lipidomics approaches, detection of lipid peroxidation byproducts are also used as markers of lipid peroxidation. 4-HNE, an α,β-unsaturated aldehyde that is derived from peroxidized products of ω-6 unsaturated fatty acids, such as linoleic acid and arachidonic acid, is an important example [609]. Immunohistochemistry with anti-4-HNE antibodies to detect 4-HNE-modified proteins serves as a surrogate of lipid peroxidation and has the big advantage of being suitable for use on formalin-fixed paraffin-embedded sections [610,611] provided that important positive and negative controls are carried out to permit firm conclusions to be drawn. Increased levels of other by-products, such as malondialdehyde and hexanoyl-lysine adduct, also indirectly support the evidence of lipid peroxidation [31,612]. However, relying solely on evidence of these by-products, including 4-HNE, as indicators of ferroptosis is insufficient due to their potential lack of specificity [583], because they can also yield “positive results” in non-ferroptotic cells due to general oxidative stress [613]. This is particularly problematic for cells lacking obvious morphological nuclear changes.

Elevated staining intensity with anti-TfR1 antibodies has been recently proposed as a potential marker for cells undergoing ferroptosis [170]. Immunofluorescence with TfR1 antibodies, including the 3F3-FMA clone, demonstrates increased labeling in total and membrane-localized components when staining ferroptotic cultured cells. Upregulation of *Ptgs2* and *CHAC1* has been reported as gene expression signatures of ferroptosis [28,29]. Nonetheless, it is crucial to note that these changes in gene expression can be also found under non-ferroptotic conditions. Thus, a careful consideration of the specificity of these gene and protein signatures is necessary when using them as indirect ferroptosis markers.

##### 6.3.2.3 Rescuing effect by ferroptosis inhibitors

In the case of predominantly ferroptotic cell death, treatment with ferroptosis-specific inhibitors should effectively prevent the demise (see chapter 3.7). While ferrostatin-1 is effective for *in vitro* ferroptosis prevention, it is barely suitable for *in vivo* use because of its instability in plasma [614]. Thus, more *in vivo* stable ferroptosis inhibitors, such as liproxstatin-1 and next generation ferrostatins, are preferred for animal studies. Evaluating ferroptosis in clinical samples is challenging, including the difficulty of obtaining optimal sample collection for (oxi)lipidomics studies and the inability to assess the impact of ferroptosis inhibitor intervention unless when performed in a clinical trial.

#### 6.3.3 State-of-the-art detection methods for iron and selenium: relevance in the context of ferroptosis

Accurate detection and quantification of iron and selenium play a crucial role in understanding their fundamental functions in biological processes and disease development [615]. In this section, we discuss cutting-edge methodologies for detecting Fe and Se, including fluorescence indicators and MS-based techniques. Fluorescence indicators such as calcein, IP-1, and Rhonox-1 offer highly sensitive and specific monitoring of LIPs in cells and tissues. MS techniques, such as conventional inductively coupled plasma mass spectrometry (ICP-MS), capillary electrophoresis-inductively coupled plasma mass spectrometry (CE-ICP-MS), and high-performance liquid chromatography (HPLC), provide precise quantification, speciation analysis, and comprehensive elemental profiling. These methodologies are particularly relevant in the context of ferroptosis.

Fluorescence probes can be categorized as “turn-off" or “turn-on" probes based on their fluorescence response to iron ions. Turn-off probes, including calcein, calcein-AM, and Phen Green SK diacetate, exhibit reduced fluorescence upon binding to iron ions. While widely used in research, these probes have limitations such as susceptibility to false-positive responses and potential interference from other metal ions such as Ni^2+^, Cu^2+^, and Co^2+^. In contrast, turn-on probes like RhoNox-1 and IP-1 show enhanced fluorescence signal upon binding to labile iron, enabling real-time monitoring and quantification of labile iron levels. Fluorescence probes, both turn-off and turn-on, serve as valuable tools for detecting and quantifying iron ions in biological systems, providing high spatial resolution [616,617]. However, careful consideration of their limitations and optimization of experimental conditions are necessary, particularly when working with complex biofluid samples, to ensure accurate and reliable results.

Detecting biological Se species, specifically selenocysteine, poses challenges due to limited selective reactions and interference from other thiols with similar properties. Existing assays, such as Probe 1 using 3′-(2,4-dinitrobenzenesulfonyl)-2′,7′-dimethylfluorescein, rely on specific pH conditions (pH 5.8) to effectively discriminate between selenocysteines and thiols. However, these conditions may not align with the typical pH range found in biological environments (around pH 7.4) [618]. Developing selective detection methods and biocompatible probes capable of overcoming interference and operating under physiological conditions remain ongoing challenges in the field of biological Se species detection.

ICP-MS has been widely recognized for its exceptional capability in measuring Fe and Se in biological and clinical samples, such as plasma and cerebrospinal fluid, for over two decades [619]. It offers high sensitivity and linearity, covering a broad concentration range. Simultaneous multi-element analysis makes ICP-MS suitable for high-throughput studies and comprehensive elemental profiling. Recent advances, including dynamic reaction cell technology combined with multi-quad-MS, have further improved precision and accuracy, enabling the measurement of previously difficult-to-determine isotopes. Various advanced sample introduction systems have been developed to enhance the information density of samples in conjunction with sensitive ICP-MS detection [620].

Size-exclusion chromatography also plays a significant role in analyzing iron and selenium species in biofluids. It is a powerful separation technique based on size and molecular weight. When coupled with suitable detection methods like ICP-MS, size-exclusion chromatography becomes a valuable tool for separating, identifying, and quantifying different forms of iron and selenium. Size-exclusion chromatography provides insights into the distribution of elements to large or small proteins and low-molecular-weight compounds, contributing to our understanding of their roles in biological processes and disease mechanisms. However, size-exclusion chromatography analysis of biofluids may face challenges related to matrix effects, requiring proper sample preparation techniques to improve selectivity, accuracy, and species stability [621].

HPLC is a versatile technique used for the separation, identification, and quantification of diverse compounds, including Fe and Se species in biofluids. It offers various separation mechanisms, high resolving power, and compatibility with various detection methods, such as UV–Vis spectroscopy or ICP-MS, facilitating the sensitive detection and characterization of different forms of Fe and Se. HPLC coupled with ICP-MS is particularly useful for speciation analysis, providing information about the distribution of different iron and selenium species in biofluids. However, HPLC analysis of biofluids can be challenging due to matrix effects. Adopted sample preparation techniques can be necessary to improve selectivity and accuracy of speciation analysis while guaranteeing species stability [622].

CE-ICP-MS combines capillary electrophoresis (CE) with ICP-MS, providing enhanced speciation analysis capabilities for Fe and Se in biofluids. This powerful technique enables the separation and quantification of various metal species based on their charge-to-mass ratio. With high separation efficiency and selectivity, CE-ICP-MS allows for the identification and quantification of different chemical forms of metals, minimizing the risk of unwanted species changes. In the analysis of biofluids, CE-ICP-MS plays a crucial role by providing valuable information about the speciation and bioavailability of Fe and Se species, shedding light on their roles in cellular processes and disease mechanisms. However, to ensure accurate quantification of target species in complex biofluid matrices, optimized separation conditions and appropriate sample preparation strategies are necessary to minimize matrix effects [623,624].

In conclusion, the discussed detection methods, including fluorescence indicators, ICP-MS, CE-ICP-MS, HPLC, and size-exclusion chromatography, offer complementary and valuable tools for the quantification, speciation analysis, and characterization of Fe and Se in the context of ferroptosis. Each method has its benefits and limitations when applied to the analysis of biofluids such as plasma and cerebrospinal fluid. Fluorescence indicators provide real-time information on LIPs but are limited to qualitative or semi-quantitative analysis. ICP-MS allows for accurate quantification and simultaneous multi-element analysis but requires careful sample preparation to address matrix effects without providing information on speciation. CE-ICP-MS provides enhanced speciation analysis capabilities for Fe and Se but may require optimization for accurate quantification in complex biofluid matrices. HPLC coupled with appropriate detection methods enables the sensitive detection and characterization of different forms of Fe and Se. Size-exclusion chromatography facilitates the separation and identification of Fe and Se species based on their size and molecular weight. Researchers should consider the benefits and limitations of these methods to select the most suitable approach for their specific research needs, thereby advancing our understanding of ferroptosis and its implications in various pathological conditions.

## 7 Conclusion and future considerations

This comprehensive state-of-the-art review describes the diverse and fascinating facets of ferroptosis, ranging from basic mechanisms, metabolic processes, signaling cues and their role in disease pathogenesis to recommendations for the proper use of specific tools and technologies. Although we have made considerable progress in our understanding of many of the key aspects of ferroptosis in recent years, numerous open questions remain. For example, is there a physiological role for ferroptosis for tissue homeostasis akin to apoptosis and some other forms of regulated necrosis? Or, is it induced only under pathological conditions? The latter could be beneficial when considering pharmacological approaches to trigger or inhibit ferroptosis in cancer or degenerative diseases, respectively, as it suggests the potential for a wide therapeutic window. Likewise, which types of cancer will benefit most from ferroptosis-based therapies? Is inhibition of tumor growth alone sufficient and do ferroptotic cancer cells trigger a robust immunogenic response such as occurring in response to necroptotic cell death? Will ferroptosis-based therapies overcome the clinical problem of inefficient treatment of standard therapy-resistant so-called persister cancer cells and metastatic cancer cells? Are there additional, pharmacologically amenable targets besides GPX4, FSP1, SLC7A11 and GCLC that can be harnessed by metabolically stable ferroptosis activators/inhibitors? Do we already have the most promising next-generation ferroptosis inhibitors at hand to be tested in clinical proof-of-concept studies, such as ischemia/reperfusion injury following organ transplantation, ischemic stroke and cardiac infarction? Will large biotech and big pharma companies ever give up their reservations about redox modulatory compounds – and RTAs, in particular – as potential drugs, despite the fact that they have no protein target and instead target cell membrane-bound radical intermediates? How much more evidence is required to convince stakeholders that the poor performance of “antioxidants” used in pre-ferroptosis era clinical trials was due to poor choices and a general lack of awareness regarding the precise role of RTAs as cell death suppressors? Are there potent ferroptosis inhibitors with a different mechanism-of-action than RTAs? What impact do ferroptotic cells have on the activation of adaptive and innate immune cells, and are there organ-specific differences and/or crosstalk with other forms of cell death? Equally important is the question of upstream disease-causing mechanisms, e.g. in neurodegenerative diseases and ischemia/reperfusion events. Finally, robust biomarkers and pharmacodynamic markers must be uncovered and validated to facilitate the design and conduct of clinical trials. The foregoing should make clear that there are still many challenges that lay ahead of us. We encourage each of established academics and industrial researchers – and newcomers, in particular – to join us in meeting these challenges, which we passionately believe will lead to promising new treatments for a wide variety of degenerative diseases.

## Declaration of gererative AI and AI-assisted technologies in the writing process

This work was prepared without help of AI.

## CRediT authorship contribution statement

**Carsten Berndt:** Writing – review & editing, Writing – original draft, Conceptualization, chapters 3.1, 3.3, 5. **Hamed Alborzinia:** Writing – review & editing, Writing – original draft, chapter 4.1. **Vera Skafar Amen:** Writing – review & editing, Writing – original draft, chapter 2.2.4. **Scott Ayton:** Writing – review & editing, Writing – original draft, chapter 4.4. **Uladzimir Barayeu:** Writing – review & editing, Writing – original draft, chapter 2.2.6. **Alexander Bartelt:** Writing – review & editing, Writing – original draft, chapter 3.5. **Hülya Bayir:** Writing – review & editing, Writing – original draft, chapter 6.2. **Christina M. Bebber:** Writing – review & editing, Writing – original draft, chapter 4.1. **Kivanc Birsoy:** Writing – review & editing, Writing – original draft, chapter 2.2.7. **Jan P. Böttcher:** Writing – review & editing, Writing – original draft, chapter 4.3. **Simone Brabletz:** Writing – review & editing, Writing – original draft, chapter 2.2.1. **Thomas Brabletz:** Writing – review & editing, Writing – original draft, chapter 2.2.1. **Ashley R. Brown:** Writing – review & editing, Writing – original draft, chapter 3.6. **Bernhard Brüne:** Writing – review & editing, Writing – original draft, chapter 3.4. **Giorgia Bulli:** Writing – review & editing, Writing – original draft, chapter 4.4. **Alix Bruneau:** Writing – review & editing, Writing – original draft, chapter 3.4. **Quan Chen:** Writing – review & editing, Writing – original draft, chapter 2.2.9. **Gina M. DeNicola:** Writing – review & editing, Writing – original draft, chapter 2.2.5. **Tobias P. Dick:** Writing – review & editing, Writing – original draft, chapter 2.2.6. **Ayelén Distéfano:** Writing – review & editing, Writing – original draft, chapter 5. **Scott J. Dixon:** Writing – review & editing, Writing – original draft, chapters 1.1, 2.1.1. **Jan B. Engler:** Writing – review & editing, Writing – original draft, chapter 4.3. **Julia Esser-von Bieren:** Writing – review & editing, Writing – original draft, chapter 3.4. **Maria Fedorova:** Writing – review & editing, Writing – original draft, chapter 6.2. **José Pedro Friedmann Angeli:** Writing – review & editing, Writing – original draft, chapters 2.2.4, 6.3.2. **Manuel A. Friese:** Writing – review & editing, Writing – original draft, chapter 4.3. **Dominic C. Fuhrmann:** Writing – review & editing, Writing – original draft, chapter 3.4. **Ana J. García-Sáez:** Writing – review & editing, Writing – original draft, chapter 2.2.8. **Karolina Garbowicz:** Writing – review & editing, Writing – original draft, chapter 4.1. **Magdalena Götz:** Writing – review & editing, Writing – original draft, chapter 4.4. **Wei Gu:** Writing – review & editing, Writing – original draft, chapter 4.1. **Linda Hammerich:** Writing – review & editing, Writing – original draft, chapter 3.4. **Behrouz Hassannia:** Writing – review & editing, Writing – original draft, chapter 1.2. **Xuejun Jiang:** Writing – review & editing, Writing – original draft, chapter 2.2.9. **Aicha Jeridi:** Writing – review & editing, Writing – original draft, chapter 4.2. **Yun Pyo Kang:** Writing – review & editing, Writing – original draft, chapter 6.1. **Valerian E. Kagan:** Writing – review & editing, Writing – original draft, chapter 6.2. **David B. Konrad:** Writing – review & editing, Writing – original draft, chapter 3.6. **Stefan Kotschi:** Writing – review & editing, Writing – original draft, chapter 3.5. **Peng Lei:** Writing – review & editing, Writing – original draft, chapter 4.4. **Marlène Le Tertre:** Writing – review & editing, Writing – original draft, chapter 2.2.3. **Sima Lev:** Writing – review & editing, Writing – original draft, chapter 3.5. **Deguang Liang:** Writing – review & editing, Writing – original draft, chapter 2.2.9. **Andreas Linkermann:** Writing – review & editing, Writing – original draft, chapter 4.5. **Carolin Lohr:** Writing – review & editing, Writing – original draft, chapter 4.1. **Svenja Lorenz:** Writing – review & editing, Writing – original draft, chapter 2.1.1. **Tom Luedde:** Writing – review & editing, Writing – original draft, chapter 4.1. **Axel Methner:** Writing – review & editing, Writing – original draft, chapter 5. **Bernhard Michalke:** Writing – review & editing, Writing – original draft, chapter 6.3.3. **Anna V. Milton:** Writing – review & editing, Writing – original draft, chapter 3.6. **Junxia Min:** Writing – review & editing, Writing – original draft, chapter 2.2.3. **Eikan Mishima:** Writing – review & editing, Writing – original draft, chapters 2.2.2, 6.3.2. **Sebastian Müller:** Writing – review & editing, Writing – original draft, chapter 2.2.3. **Hozumi Motohashi:** Writing – review & editing, Writing – original draft, chapter 3.2. **Martina U. Muckenthaler:** Writing – review & editing, Writing – original draft, chapter 2.2.3. **Shohei Murakami:** Writing – review & editing, Writing – original draft, chapter 3.2. **James A. Olzmann:** Writing – review & editing, Writing – original draft, chapter 2.1.2. **Gabriela Pagnussat:** Writing – review & editing, Writing – original draft, chapter 5. **Zijan Pan:** Writing – review & editing, Writing – original draft, chapter 3.4. **Thales Papagiannakopoulos:** Writing – review & editing, Writing – original draft, chapter 3.2. **Lohans Pedrera Puentes:** Writing – review & editing, Writing – original draft, chapter 2.2.8. **Derek A. Pratt:** Writing – review & editing, Writing – original draft, chapters 2.2.1, 3.7. **Bettina Proneth:** Writing – review & editing, Writing – original draft, chapter 4.2. **Lukas Ramsauer:** Writing – review & editing, Writing – original draft, chapter 4.3. **Raphael Rodriguez:** Writing – review & editing, Writing – original draft, chapter 2.2.3. **Yoshiro Saito:** Writing – review & editing, Writing – original draft, chapter 2.2.4. **Felix Schmidt:** Writing – review & editing, Writing – original draft, chapter 5. **Carina Schmitt:** Writing – review & editing, Writing – original draft, chapter 6.3.1. **Almut Schulze:** Writing – review & editing, Writing – original draft, chapter 2.2.1. **Annemarie Schwab:** Writing – review & editing, Writing – original draft, chapter 2.2.1. **Anna Schwantes:** Writing – review & editing, Writing – original draft, chapter 3.4. **Mariluz Soula:** Writing – review & editing, Writing – original draft, chapter 2.2.7. **Benedikt Spitzlberger:** Writing – review & editing, Writing – original draft, chapter 3.4. **Brent R. Stockwell:** Writing – review & editing, Writing – original draft, chapters 1.1, 3.6, 3.7. **Leonie Thewes:** Writing – review & editing, Writing – original draft, chapter 3.3. **Oliver Thorn-Seshold:** Writing – review & editing, Writing – original draft, chapter 6.3.1. **Shinya Toyokuni:** Writing – review & editing, Writing – original draft, chapter 4.1. **Wulf Tonnus:** Writing – review & editing, Writing – original draft, chapter 4.5. **Andreas Trumpp:** Writing – review & editing, Writing – original draft, chapter 4.1. **Peter Vandenabeele:** Writing – review & editing, Writing – original draft, chapter 1.2. **Tom Vanden Berghe:** Writing – review & editing, Writing – original draft, chapter 1.2. **Vivek Venkataramani:** Writing – review & editing, Writing – original draft, chapter 6.3.3. **Felix C.E. Vogel:** Writing – review & editing, Writing – original draft, chapter 2.2.1. **Silvia von Karstedt:** Writing – review & editing, Writing – original draft, chapter 4.1. **Fudi Wang:** Writing – review & editing, Writing – original draft, chapter 2.2.3. **Frank Westermann:** Writing – review & editing, Writing – original draft, chapter 4.1. **Chantal Wientjens:** Writing – review & editing, Writing – original draft, chapter 4.3. **Christoph Wilhelm:** Writing – review & editing, Writing – original draft, chapter 4.3. **Michele Wölk:** Writing – review & editing, Writing – original draft, chapter 6.2. **Katherine Wu:** Writing – review & editing, Writing – original draft, chapter 3.2. **Xin Yang:** Writing – review & editing, Writing – original draft, chapter 4.1. **Fan Yu:** Writing – review & editing, Writing – original draft, chapter 2.2.9. **Yilong Zou:** Writing – review & editing, Writing – original draft, chapter 3.4. **Marcus Conrad:** Writing – review & editing, Writing – original draft, Conceptualization, chapters 1.1, 2.1.1, 3.7.

Chapter 1.1: **Scott J. Dixon, Brent R. Stockwell, Marcus Conrad**; 1.2: **Behrouz Hassannia, Peter Vandenabeele, Tom Vanden Berghe**; 2.1.1: **Svenja Lorenz, Marcus Conrad**; 2.1.2: **James M. Olzmann**; 2.2.1: **Simone Brabletz, Thomas Brabletz, Scott J. Dixon, Almut Schulze, Annemarie Schwab, Derek A. Pratt, Felix C. E. Vogel**; 2.2.2: **Eikan Mishima**; 2.2.3: **Marlène Le Tertre, Junxia Min, Sebastian Müller, Martina U. Muckenthaler, Raphael Rodriguez, Fudi Wang**; 2.2.4: **Vera Skafar Amen, José Pedro Friedmann Angeli, Yoshiro Saito**; 2.2.5: **Gina M. DeNicola**; 2.2.6: **Uladzimir Barayeu, Tobias P. Dick**; 2.2.7: **Kivanc Birsoy, Mariluz Soula**; 2.2.8: **Ana J. García-Sáez, Lohans Pedrera Puentes**; 2.2.9: **Quan Chen, Xuejun Jiang, Deguang Liang, Fan Yu**; 3.1: **Carsten Berndt**; 3.2: **Hozumi Motohashi, Shohei Murakami, Thales Papagiannakopoulos, Katherine Wu**; 3.3: **Carsten Berndt, Leonie Thewes**; 3.4: **Alix Bruneau, Bernhard Brüne, Julia Esser-von Bieren, Dominic C. Fuhrmann, Linda Hammerich, Zijan Pan, Anna Schwantes, Benedikt Spitzlberger, Yilong Zou**; 3.5: **Alexander Bartelt, Stefan Kotschi, Sima Lev**; 3.6: **Ashley R. Brown, David B. Konrad, Anna V. Milton, Brent R. Stockwell**; 3.7: **Derek A. Pratt, Brent R. Stockwell, Marcus Conrad**; 4.1: **Hamed Alborzinia, Christina M. Bebber, Karolina Garbowicz, Wei Gu, Carolin Lohr, Tom Luedde, Andreas Trumpp, Shinya Toyokuni, Silvia von Karstedt, Frank Westermann, Xin Yang**; 4.2: **Aicha Jeridi, Bettina Proneth**; 4.3: **Jan P. Böttcher, Jan B. Engler, Manuel A. Friese, Lukas Ramsauer, Chantal Wientjens, Christoph Wilhelm**; 4.4: **Scott Ayton, Giorgia Bulli, Magdalena Götz, Peng Lei**; 4.5: **Andreas Linkermann, Wulf Tonnus**; 5: **Carsten Berndt, Ayelén Distéfano, Axel Methner, Gabriela Pagnussat, Felix Schmidt**; 6.1: **Yun Pyo Kang**; 6.2: **Hülya Bayir, Maria Fedorova, Valerian E. Kagan, Michelle Wölk**; 6.3.1: **Carina Schmitt, Oliver Thorn-Seshold**; 6.3.2: **José Pedro Friedmann Angeli, Eikan Mishima**: 6.3.3: **Bernhard Michalke, Vivek Venkataramani**

## Declaration of competing interest

The authors declare the following financial interests/personal relationships which may be considered as potential competing interests:

SJD is a co-founder of Prothegen, Inc. and holds patents related to ferroptosis. XJ is an inventor on patents related to autophagy and cell death and holds equity of and consults for Exarta Therapeutics and Lime Therapeutics. JAO is a member of the scientific advisory board for Vicinitas Therapeutics and has patent applications related to ferroptosis. 10.13039/501100017484TP reports grants from Dracen Pharmaceuticals, Kymera Therapeutics, 10.13039/100002491Bristol-Myers Squibb, 10.13039/100016471Agios Pharmaceuticals, personal fees from Vividion Therapeutics, 10.13039/501100006004Tohoku University, and personal fees from Faeth Therapeutics outside the submitted work; in addition, 10.13039/501100017484TP has a patent for US-20210361603-A1 pending and a patent for US-20210085763-A1 pending. BRS is an inventor on patents and patent applications involving ferroptosis, holds equity in and serves as a consultant to Exarta Therapeutics, and ProJenX Inc, holds equity in Sonata Therapeutics, and serves as a consultant to Weatherwax Biotechnologies Corporation and Akin Gump Strauss Hauer & Feld LLP. TVB holds patents related to ferroptosis inhibitors. CWil is a consultant for Odyssey Therapeutics and Orphagen Pharmaceuticals. YZ is a consultant for Keen Therapeutics. BP and MC hold patents for some of the compounds described herein, and is co-founder and shareholder of ROSCUE Therapeutics.

## Data Availability

No data was used for the research described in the article.
